# Endothelial von Hippel-Lindau gene deletion causes abnormal blood and lymphatic vasculature through ectopic activation of the HIF-CXCR4 axis

**DOI:** 10.1242/dev.204519

**Published:** 2026-05-18

**Authors:** Wenling Li, Koh Nakayama, Ryo Sato, Rina Shimada, Yoshiaki Kubota, Yoh-suke Mukouyama

**Affiliations:** ^1^ Laboratory of Stem Cell and Neuro-Vascular Biology, Cell and Development Biology Center, National Heart, Lung, and Blood Institute, National Institutes of Health, Bethesda, MD 20892, USA; ^2^ Department of Pharmacology, School of Medicine, Asahikawa Medical University, Asahikawa, Hokkaido 078-8510, Japan; ^3^ Department of Organ Anatomy, Graduate School of Medicine, Tohoku University, Aoba-ku, Sendai, Miyagi 980-8575, Japan; ^4^ Department of Anatomy, Keio University School of Medicine, Shinanomachi, Shinjuku-ku, Tokyo 160-8582, Japan

**Keywords:** VHL, HIF, CXCR4, Angiogenesis, Vascular development

## Abstract

The von Hippel-Lindau (VHL) protein regulates cellular oxygen sensing by degrading hypoxia-inducible factors (HIFs) under normoxic conditions. *VHL* mutations show highly vascularized tumor across various organs due to HIF activation and upregulation of HIF-target genes such as *VEGF* in non-endothelial cells (ECs), influencing neighboring ECs and triggering abnormal angiogenesis. Whether *VHL* mutations in ECs also contribute to abnormal angiogenesis remains unclear. To address this question, we utilized a well-characterized skin vasculature model that encompasses the processes of vascular patterning and arterial/venous development, to investigate vascular development in mice with an EC-specific *Vhl* deletion. The mutants exhibited abnormal vascular network formation and embryonic lethal. Mechanistically, the *Vhl* deletion led to ectopic expression of the chemokine receptor CXCR4 through HIF stabilization in ECs. Treatment with AMD3100, a CXCR4 antagonist, partially restored vascular abnormalities caused by *Vhl* deletion in ECs. Additionally, publicly available single-cell RNA-sequencing data from ECs of individuals with VHL syndrome supports our findings, indicating that *VHL* mutations in ECs contribute to abnormal angiogenesis through ectopic activation of the HIF-CXCR4 signaling axis.

## Teaser

**Summary:** An endothelial cell-specific *Vhl* mutation in a well-characterized skin vasculature model reveals that *Vhl* mutations in endothelial cells contribute to abnormal angiogenesis through ectopic activation of the HIF-CXCR4 signaling axis.

## INTRODUCTION

The von Hippel-Lindau (VHL) protein plays a crucial role in cellular responses to oxygen availability, particularly through its interactions with hypoxia-inducible factors (HIFs). Under normoxic conditions, the VHL protein acts as an E3 ubiquitin ligase, targeting HIFs for proteasomal degradation, thus maintaining cellular homeostasis. However, in hypoxic environments, HIF stabilization occurs, leading to the transcriptional activation of hypoxia-inducible genes involved in angiogenesis, glycolysis and erythropoiesis (Kim and Kaelin, 2003; de Rojas-P et al., 2021). VHL syndrome is an autosomal dominant disorder characterized by germline mutations in the *VHL* gene that impair the degradation of HIFs. This leads to the constitutive activation of HIFs, driving HIF-mediated transcriptional programs in tumor cells, independently of oxygen levels. One of the hallmarks of VHL syndrome is the development of highly vascularized tumors, driven by dysregulated angiogenesis. Among the key players in angiogenesis regulation in tumor cells is vascular endothelial growth factor (VEGF) and its receptors, which affect the neighboring endothelial cells (ECs) and induce tumor abnormal angiogenesis (Wizigmann-Voos et al., 1995; Siemeister et al., 1996; Chappell et al., 2019). But the impact of the VHL-HIF oxygen-sensing pathway in ECs on angiogenesis remains unknown.

Chemokine receptor 4 (CXCR4), a G-protein-coupled receptor, is a hypoxia-responsive gene and has diverse functions in mediating cell migration, proliferation and survival in various physiological processes. CXCR4 binds to its ligand, CXCL12, and activates downstream signaling pathways to regulate immune cell trafficking, stem cell homing and tissue regeneration (Doring et al., 2014; Moll and Ransohoff, 2010; Bianchi and Mezzapelle, 2020). CXCL12-CXCR4 signaling is also known to control organ- and tissue-specific blood vessel patterning (Cavallero et al., 2015; Ivins et al., 2015; Li et al., 2013, 2021; Tachibana et al., 1998). It has been reported that VHL negatively regulates CXCR4 expression through HIFs *in vitro* (Staller et al., 2003), and that HIF-1α can directly regulate CXCR4 expression, promoting tumor cell migration, invasion and angiogenesis under hypoxic conditions (Liu et al., 2006; Schioppa et al., 2003; Wu et al., 2022). Interestingly, the expression of CXCL12 and CXCR4 were upregulated in both tumor cells and ECs in individuals with VHL syndrome (Liang et al., 2007; Zagzag et al., 2005).

Previous studies on embryonic angiogenesis involving EC-specific deletion of *Vhl* using EC-specific *Tie2-Cre* driver mice are limited due to embryonic lethality caused by impaired vascular formation in the mutant placental labyrinth (Tang et al., 2006). To better understand the cell-autonomous role of VHL in ECs, we first generated a tamoxifen inducible EC-specific deletion of *Vhl* gene by crossing *Vhl-flox* mice with EC-specific *Cad5-BAC-Cre^ERT2^* driver mice. Second, to study the intricate processes of angiogenesis, we employed the developing skin vasculature as a model system, as it is known to have a highly stereotypic and recognizable vascular branching pattern (Bautch and Mukouyama, 2022; Li et al., 2013; Mukouyama et al., 2002). By embryonic day (E) 13.5, the capillary network extends throughout the skin, and by E15.5, it undergoes remodeling to establish a branched, hierarchical vascular structure (Bautch and Mukouyama, 2022; Li et al., 2013; Mukouyama et al., 2002). This inducible inactivation of *Vhl* in ECs allows us to circumvent early embryo lethality, as previously reported (Tang et al., 2006). Our data indicate that EC-specific *Vhl* deletion results in abnormal and immature vascular phenotypes in the developing skin vasculature model, attributed to ectopic CXCR4 expression via HIF stabilization. Remarkably, these phenotypes resemble those observed in mutants with EC-specific *Cxcr4* overexpression (Li et al., 2021). Furthermore, treatment with the CXCR4 antagonist AMD3100 partially rescues the vascular defects in EC-specific *Vhl* mutant embryos. Consistent with our findings in embryonic angiogenesis, analysis of publicly available single-cell RNA-Seq data from ECs of individuals with clear cell renal cell carcinoma (ccRCC) reveals elevated *CXCR4* expression in ECs from ccRCC tumors harboring *VHL* loss-of-function mutations, compared to those in ccRCC tumors without *VHL* mutations and benign adjacent kidney tissue. Collectively, these data suggest that *VHL* deletions in ECs promote increased CXCR4 expression through constitutive HIF stabilization, leading to abnormal vascular network formation.

## RESULTS

### EC-specific *Vhl* deletion results in hemorrhage-like phenotypes

**Fig. 1.**
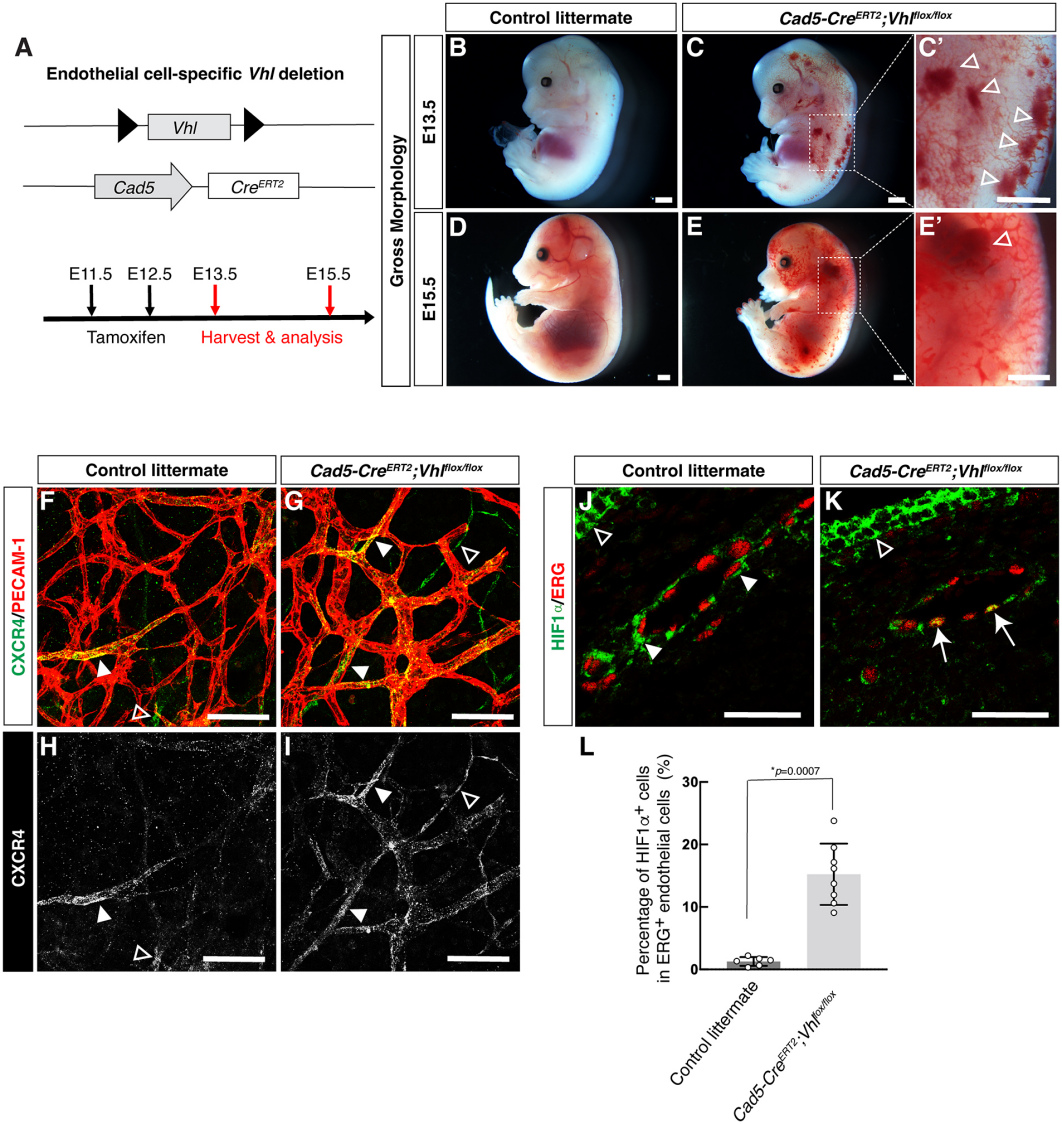
**Ectopic CXCR4 expression and HIF1α stabilization in the skin vasculature of endothelial cell-specific *Vhl* deletion mutant embryos.** (A) Diagram illustrating the generation of endothelial cell (EC)-specific *Vhl* deletion mice by crossing *Vhl*-floxed mice with tamoxifen-inducible EC-specific *Cre* driver, *Cad5-BAC-Cre^ERT2^* mice. The Cre-mediated excision activity was achieved by administering 2 mg tamoxifen by intraperitoneal injection at E11.5-E12.5, and embryos were harvested at E13.5 or E15.5 for analysis. (B-E) Gross morphology of control littermates (B,D) and *Cad5-BAC-Cre^ERT2^;Vhl^flox/flox^* embryos (C,E) collected from the same litter and photographed at the same magnification for each time point (E13.5 and E15.5). A hemorrhage-like phenotype (C′,E′, open arrowheads) was found in *Cad5-BAC-Cre^ERT2^; Vhl^flox/flox^* embryos. The areas outlined in C and E are magnified in C′ and E′, respectively. Scale bars: 1 mm. (F-I) Whole-mount immunohistochemical analysis of limb skin from E13.5 control littermates (F,H) and *Cad5-BAC-Cre^ERT2^;Vhl^flox/flox^* embryos (G,I) with antibodies to the pan-EC marker PECAM1 (F,G, red) and CXCR4 (F,G, green; H,I, white). White arrowheads indicate representative CXCR4-expressing ECs within the capillary network. Open arrowheads indicate CXCR4-expressing peripheral nerves. Scale bars: 100 µm. (J,K) Section immunohistochemical analysis of the dermal vasculature in limb skin from E13.5 control littermates and *Cad5-BAC-Cre^ERT2^;Vhl^flox/flox^* embryos with antibodies to HIF1α (green) and the EC nuclei marker ERG (red). Arrows indicate the representative colocalization of HIF1α and ERG in the nuclei of ECs (K). White arrowheads indicate HIF1α in the cytoplasm of ECs. Open arrowheads indicate HIF1α in the cytoplasm of epidermis cells. White dotted lines indicate vessel lumens. Scale bars: 50 µm. (L) Quantification of the percentage of ERG^+^ ECs showing colocalization of HIF1α and ERG in EC nuclei in the skin of control littermates (*n*=6) and *Cad5-BAC-Cre^ERT2^;Vhl^flox/flox^* mutants (*n*=8). Data are mean±s.d. A significance threshold of *P<*0.05 was considered to indicate a statistically significant difference. According to the nonparametric Mann–Whitney test, there is a statistically significant increase (*P=0.0007) in the colocalization of HIF1α and ERG in EC nuclei in the skin of *Cad5-BAC-Cre^ERT2^;Vhl^flox/flox^* mutants compared to control littermates.

To delete *Vhl* in ECs, we generated an inducible EC-specific *Vhl* conditional knockout mice by crossing *Vhl-flox* mice with EC-specific *Cad5-BAC-Cre^ERT2^* driver mice. We opted to induce *Vhl* deletion in *Cad5-BAC-Cre^ERT2^; Vhl^flox/flox^* embryos through a 2 mg tamoxifen administration at E11.5 and E12.5, targeting the processes of dermal vascular development from primitive capillary network formation to subsequent hierarchical vascular network formation (Fig. 1A). *Cad5-BAC-Cre^ERT2^;Vhl^flox/flox^* mutants exhibited hemorrhage-like phenotypes compared to control littermates at E13.5 and E15.5 (Fig. 1B versus C, D versus E; C′ and E′, open arrowheads). Additionally, the mutants developed mild but significant edema at E16.5 (Fig. S1) and died around E17.5. Such hemorrhage-like phenotypes are consistent with the observations from previous studies involving EC-specific *Vhl* deletion in mouse embryos (Tang et al., 2006) and postnatal mouse retina (Wang et al., 2018).

### EC-specific *Vhl* deletion dysregulates CXCR4 expression through HIF activation

We hypothesized that abnormal vascular network formation in *Cad5-BAC-Cre^ERT2^;Vhl^flox/flox^* mutant embryos results from the constitutive stabilization of hypoxia-inducible factors (HIFs), which leads to the uncontrolled activation of HIF-target genes. To test this, we performed fluorescence-activated cell sorting (FACS) to isolate ECs (CD45^−^/Ter119^−^/PECAM1^+^) from the skin of both E13.5 *Cad5-BAC-Cre^ERT2^;Vhl^flox/flox^* mutant embryos and control littermates, followed by quantitative reverse transcription PCR (RT-qPCR) analysis of mRNA expression for HIF-target genes related to vascular development. After confirming that the mRNA level of *Vhl* gene was significantly decreased in the skin ECs of *Cad5-BAC-Cre^ERT2^;Vhl^flox/flox^* mutant embryos compared to control littermates (Fig. S2A), we first examined the expression of previously reported HIF-target metabolic genes, which play an important role in vascular development (Bierhansl et al., 2017) and are also involved in cardiovascular disease (Ullah and Wu, 2021). These genes include phosphofructo-2-kinase/fructose-2,6-biphosphatase 3 (*Pfkfb3*), aldolase A (*Aldoa*), glucose tranporter1 [*Glut1*, also known as solute carrier family 2 (facilitated glucose transporter), member 1 (*Slc2a1*)], hexokinase 2 (*Hk2*), pyruvate dehydrogenase kinase (*Pdk1*), phosphoglycerate kinase 1 (*Pgk1*) and lactate dehydrogenase A (*Ldha*) (Ullah and Wu, 2021; Du et al., 2021). No significant alterations in the expression of these metabolic genes in ECs were observed between *Cad5-BAC-Cre^ERT2^;Vhl^flox/flox^* mutants and their control littermates (Fig. S2B). We further examined the expression of previously reported HIF-target angiogenic genes, including angiopoietin 1 (*Angpt1*) (Park et al., 2016), angiopoietin 2 (*Angpt2*) (Simon et al., 2008), *Cxcr4* (Schioppa et al., 2003), endoglin (*Eng*) (Sanchez-Elsner et al., 2002), endothelin 1 (*Edn1*) (Yamashita et al., 2001), *Notch1* (Gustafsson et al., 2005), *Pdgfb* (Schito et al., 2012) and *Vegfa* (Forsythe et al., 1996). Notably, the expression of *Cxcr4* and *Vegfa* was significantly increased in ECs from *Cad5-BAC-Cre^ERT2^;Vhl^flox/flox^* mutant embryos compared to their control littermates (Fig. S2C). We then conducted whole-mount immunohistochemistry staining on limb skin from E13.5 *Cad5-BAC-Cre^ERT2^;Vhl^flox/flox^* mutant embryos and their control littermates to examine CXCR4 and VEGFA protein expression in the skin vasculature. As expected from our previous studies (Li et al., 2013), a subset of ECs in the skin vasculature of E13.5 control embryos express CXCR4 (Fig. 1F,H, arrowheads). In contrast, a greater number of CXCR4-expessing ECs was observed in the skin vasculature of E13.5 *Cad5-BAC-Cre^ERT2^;Vhl^flox/flox^* mutant embryos (Fig. 1F versus G, H versus I, white arrowheads), with no changes in the total number of ECs in that region (Fig. S3). Interestingly, despite the significant increase in *Vegfa* mRNA expression in the mutant ECs, we did not detect any difference in VEGFA protein expression in the skin vasculature between E13.5 *Cad5-BAC-Cre^ERT2^;Vhl^flox/flox^* mutant embryos and their control littermates (Fig. S4A-D, arrows; E). These findings led us to focus on the cell-autonomous role of the VHL-HIF-CXCR4 axis in ECs during vascular network formation.

Mechanistically, VHL loss leads to the stabilization and nuclear localization of HIFs, which transcriptionally activate the expression of HIF-target genes. To assess the impact of *Vhl* deletion on HIF nuclear localization in ECs during vascular network formation, we conducted immunostaining of limb skin from E13.5 *Cad5-BAC-Cre^ERT2^;Vhl^flox/flox^* mutant embryos and their control littermates using antibodies to HIF1α (Fig. 1J,K, green) and the EC nucleus marker ERG (Fig. 1J,K, red). In control littermates, HIF1α and ERG showed minimal colocalization in EC nuclei (Fig. 1J, white arrowheads; L). In contrast, a significant increase in HIF1α-ERG colocalization was observed in EC nuclei in *Cad5-BAC-Cre^ERT2^;Vhl^flox/flox^* mutant embryos (Fig. 1K, arrows; L). We should note that, due to technical issues, we were only able to capture ECs that displayed a strong and significant signal in the HIF1α antibody staining of EC-specific *Vhl* deletion mutants. Therefore, we acknowledge that we may not have captured all ECs expressing nuclear HIF1α in these mutants.

**Fig. 2.**
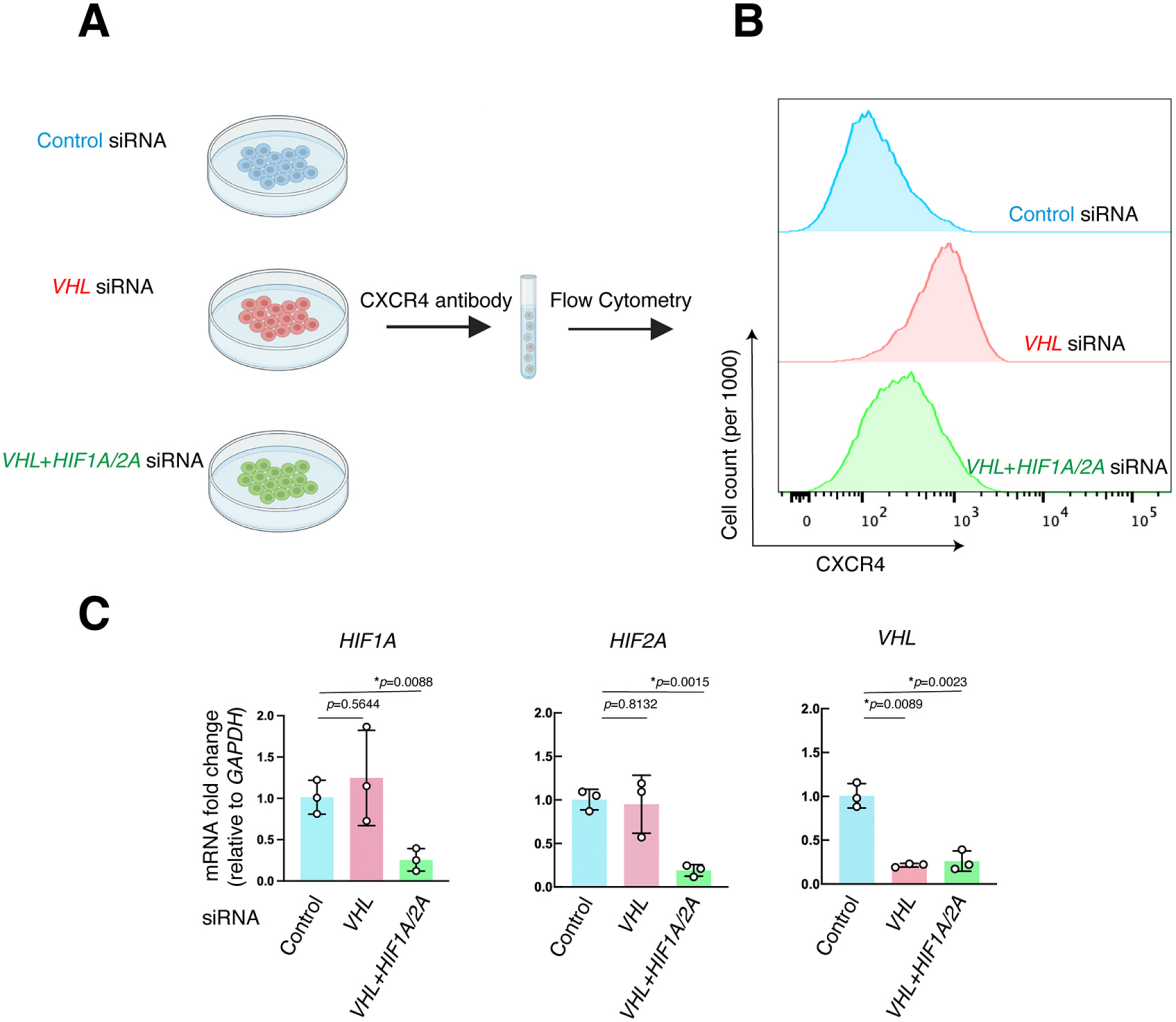
***VHL* knockdown leads to CXCR4 induction through HIF1/2 in cultured ECs.** (A) Schematic illustration of knockdown experiments in human umbilical vein endothelial cells (HUVECs) treated with control siRNA (blue), *VHL* siRNA (red) and *VHL/HIF1A/HIF2A* siRNA (green). Created in BioRender by Li, W., 2026. https://BioRender.com/amh6xre. This figure was sublicensed under CC-BY 4.0 terms. (B) Representative flow cytometry data analyzing CXCR4 expression in HUVECs treated with control siRNA (blue), *VHL* siRNA (red) and *VHL/HIF1A/HIF2A* siRNA (green). (C) The expression of *HIF1A*, *HIF2A* and *VHL* in HUVECs treated with control siRNA (blue), *VHL* siRNA (red) and *VHL/HIF1A/HIF2A* siRNA (green) was assessed by RT-qPCR (*n*=3). Data are mean±s.d. Statistical *P* values were determined using the nonparametric Mann–Whitney test with a significance threshold of *P<*0.05. A statistically significant reduction in relative mRNA expression levels was observed compared to HUVECs treated with control siRNA. *HIF1A* and *HIF2A* expression levels were significantly reduced in HUVECs treated with *VHL/HIF1A/HIF2A* siRNA (*P=0.0088 for *HIF1A* and *P=0.0015 for *HIF2A*)*. VHL* expression was also significantly reduced in HUVECs treated with *VHL* siRNA and *VHL/HIF1A/HIF2A* siRNA (*P*=0.0089 for *VHL* siRNA and *P=0.0023 for *VHL/HIF1A/HIF2A* siRNA).

To further assess whether VHL loss leads to the upregulation of CXCR4 expression through HIFs, we used cultured human umbilical vein endothelial cells (HUVECs) and compared CXCR4 protein expression between control, *VHL* knockdown and *VHL/HIF1A/HIF2A* triple-knockdown HUVECs (Fig. 2A-C). Consistent with our *in vivo* findings (Fig. 1F-I), *VHL* knockdown HUVECs exhibited increased CXCR4 expression, compared to control siRNA-treated HUVECs (Fig. 2A,B). In contrast, *VHL/HIF1A/HIF2A* triple-knockdown HUVECs showed a significant reduction of CXCR4 expression compared to *VHL* knockdown HUVECs (Fig. 2A,B), suggesting that HIFs mediate the upregulation of CXCR4 expression in *VHL*-deficient ECs.

### EC-specific *Vhl* deletion mutants exhibit abnormal vascular network formation nearly identical to those observed in EC-specific *Cxcr4* overexpression mutants

To assess the impact of the VHL-HIF-CXCR4 axis in ECs during vascular network formation, we conducted side-by-side comparison between EC-specific *Vhl* deletion mutants and EC-specific *Cxcr4* overexpression mutants. We have previously generated an inducible EC-specific *Cxcr4* conditional overexpression mice by crossing *ROSA-LSL-Cxcr4-hGFP* mice with EC-specific *Cad5-BAC-Cre^ERT2^* driver mice (Li et al., 2021). We opted to induce CXCR4 expression in *Cad5-BAC-Cre^ERT2^;ROSA-LSL-Cxcr4-hGFP* embryos through a 2 mg tamoxifen administration at E10.5, E11.5 and E12.5 (Fig. S5A). *Cad5-BAC-Cre^ERT2^;ROSA-LSL-Cxcr4-hGFP* embryos exhibited hemorrhage-like phenotypes compared to control littermates at E13.5 and E15.5 (Fig. S5B versus C, D versus E; C′ and E′, open arrowheads). Additionally, the mutants developed significant edema at E15.5 and died around E17.5. These data suggest EC-specific *Vhl* deletion mutants (Fig. 1B-E′) exhibit nearly identical phenotypes to those of EC-specific *Cxcr4* overexpression mutants (Fig. S5B-E′).

**Fig. 3.**
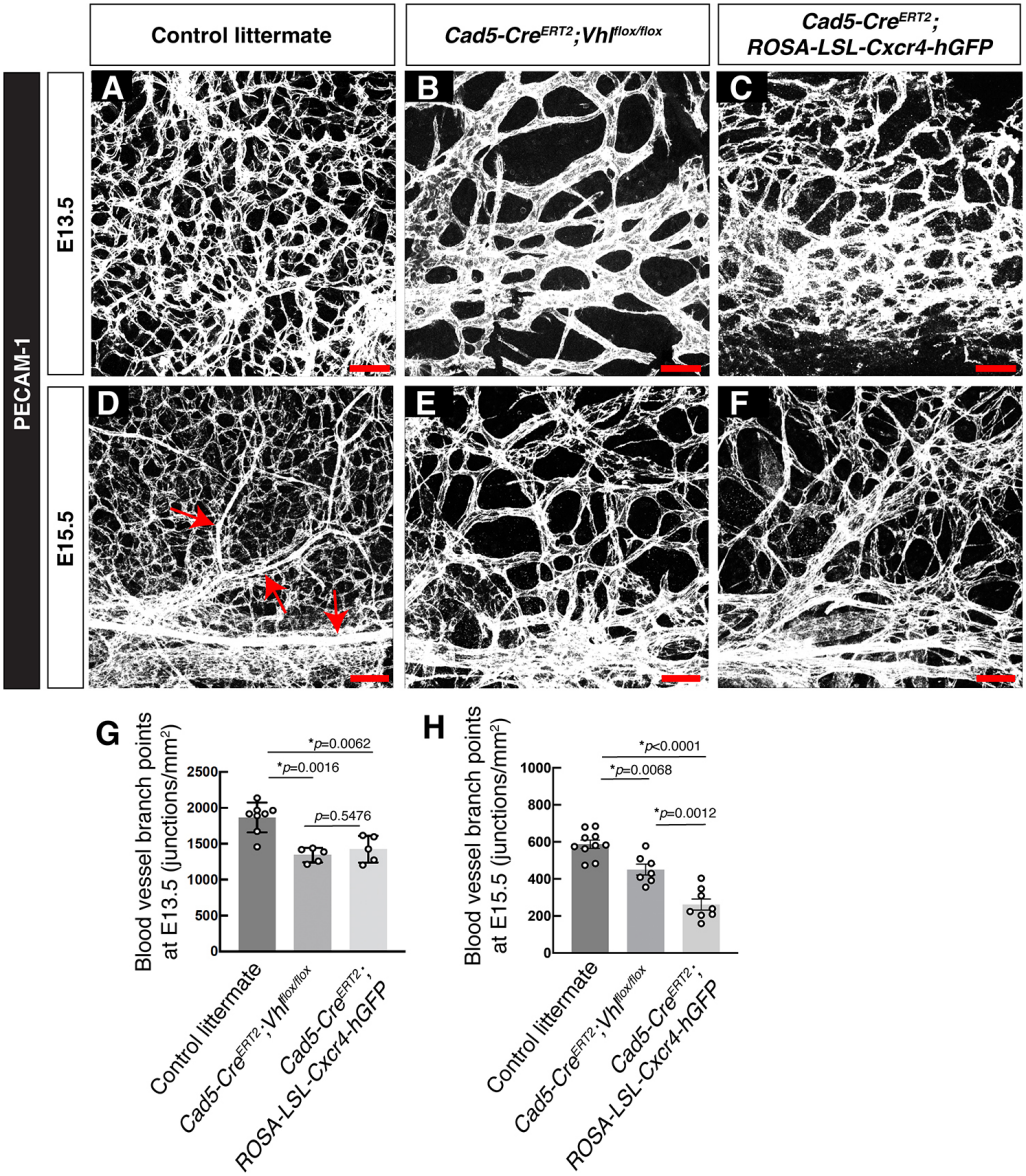
**Abnormal vascular network formation in the skin of EC-specific *Vhl* deletion and EC-specific *Cxcr4* overexpression mutants.** (A-F) Whole-mount immunohistochemical analysis of limb skin from E13.5 (A-C) and E15.5 (D-F) in control littermates (A,D), *Cad5-BAC-Cre^ERT2^;Vhl^flox/flox^* (B,E) and *Cad5-BAC-Cre^ERT2^;ROSA-LSL-Cxcr4-hGFP* (C,F) embryos with antibody to PECAM1. Arrows indicate representative remodeled blood vessels. Scale bars: 100 µm. (G) Quantification of PECAM1^+^ blood vessel branch points (junctions per mm^2^) by AngioTool at E13.5 (*n*=8 for control littermates, *n*=5 for both *Cad5-BAC-Cre^ERT2^;Vhl^flox/flox^* and *Cad5-BAC-Cre^ERT2^;ROSA-LSL-Cxcr4-hGFP* embryos). Data are mean±s.d. Statistical *P* values were determined using the nonparametric Mann–Whitney test and a significance threshold of *P<*0.05 was considered to indicate a statistically significant reduction in vascular branch points compared to control littermates (*P=0.0016 for *Cad5-BAC-Cre^ERT2^;Vhl^flox/flox^* and *P=0.0062 for *Cad5-BAC-Cre^ERT2^;ROSA-LSL-Cxcr4-hGFP* embryos). There is no significant difference between *Cad5-BAC-Cre^ERT2^;Vhl^flox/flox^* and *Cad5-BAC-Cre^ERT2^;ROSA-LSL-Cxcr4-hGFP* embryos. (H) Quantification of PECAM1^+^ blood vessel branch points (junctions per mm^2^) by AngioTool at E15.5 (*n*=10 for control littermates, *n*=7 for *Cad5-BAC-Cre^ERT2^;Vhl^flox/flox^* and *n*=8 for *Cad5-BAC-Cre^ERT2^;ROSA-LSL-Cxcr4-hGFP*). Data are mean±s.d. Statistical *P* values were determined using the nonparametric Mann–Whitney test and a significance threshold of *P<*0.05 was considered to indicate a statistically significant reduction in vascular branch points compared to control littermates (*P=0.0068 for *Cad5-BAC-Cre^ERT2^;Vhl^flox/flox^* and *P<0.0001 for *Cad5-BAC-Cre^ERT2^;ROSA-LSL-Cxcr4-hGFP* embryos). There were significant differences in the branching points between *Cad5-BAC-Cre^ERT2^;Vhl^flox/flox^* and *Cad5-BAC-Cre^ERT2^;ROSA-LSL-Cxcr4-hGFP* embryos.

We then investigated vascular network formation in EC-specific *Vhl* deletion mutants and EC-specific *Cxcr4* overexpression mutants using whole-mount staining of limb skin for the pan-endothelial cell marker PECAM1. In control littermates at E13.5, a honeycomb-shaped capillary network was observed (Fig. 3A). In contrast, both *Cad5-BAC-Cre^ERT2^;Vhl^flox/flox^* mutants and *Cad5-BAC-Cre^ERT2^;ROSA-LSL-Cxcr4-hGFP* mutants exhibited enlarged capillaries with reduced branching complexity (Fig. 3A versus B, A versus C; G). By E15.5, the capillary network in control littermates had remodeled into a branched hierarchical vascular network (Fig. 3D: remodeled blood vessels indicated by arrows). Notably, both *Cad5-BAC-Cre^ERT2^;Vhl^flox/flox^* mutants and *Cad5-BAC-Cre^ERT2^;ROSA-LSL-Cxcr4-hGFP* mutants showed a disorganized vascular network with enlarged capillaries, as well as reduced branching complexity (Fig. 3D versus E, D versus F; H). It has been reported that vessel pruning, i.e. the regression of selected vascular branches, significantly influences the formation of vascular network (Fonseca et al., 2020). Vessel pruning is characterized by the loss of ECs and the retention of type IV collagen (Col IV)-positive empty sleeves (Wen et al., 2023; Fonseca et al., 2020; Franco et al., 2015). To determine whether the abundant capillaries and the reduced vascular branching complexity (Fig. 3G,H) in the skin vasculature of both *Cad5-BAC-Cre^ERT2^;Vhl^flox/flox^* mutants and *Cad5-BAC-Cre^ERT2^;ROSA-LSL-Cxcr4-hGFP* mutants were caused by increased vessel pruning, we examined the Col IV^+^/PECAM1^−^ pruning vessels in the skin capillaries at E15.5 (Fig. S6A-C″, arrows). Our observations revealed a significant increase in vessel pruning events in both *Cad5-BAC-Cre^ERT2^;Vhl^flox/flox^* mutants and *Cad5-BAC-Cre^ERT2^;ROSA-LSL-Cxcr4-hGFP* mutants compared to control littermates at E15.5 (Fig. S6D).

Our previous studies have shown that in developing skin, small- and mid-diameter remodeled blood vessels co-branch with sensory nerves (Bautch and Mukouyama, 2022; Li et al., 2013; Mukouyama et al., 2002). Given that there were no apparent changes in the branching pattern of Tuj1^+^ nerves in both *Cad5-BAC-Cre^ERT2^;Vhl^flox/flox^* mutants and *Cad5-BAC-Cre^ERT2^;ROSA-LSL-Cxcr4-hGFP* mutants compared to control littermates at E15.5, the nerve-vessel co-branching was severely disrupted in these mutants (Fig. S7A versus B and C). These data suggest that both EC-specific *Vhl* deletion mutants and EC-specific *Cxcr4* overexpression mutants lead to similar abnormalities in vascular network formation and nerve-vessel co-branching.

### EC-specific *Vhl* deletion mutants and EC-specific *Cxcr4* overexpression mutants exhibit similar abnormalities in arterial and venous development

**Fig. 4.**
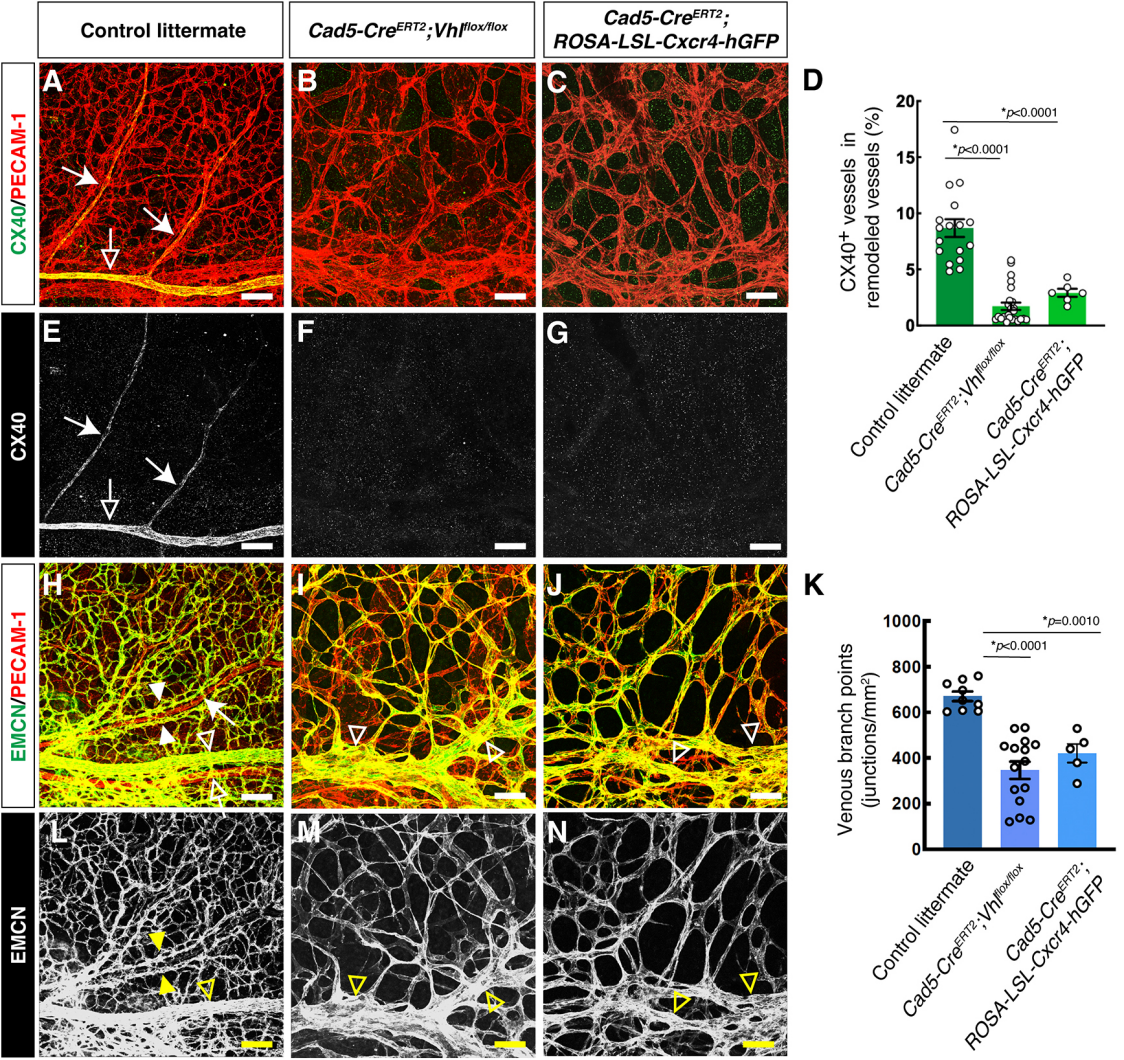
**Defective arterial and venous development in the skin of EC-specific *Vhl* deletion and EC-specific *Cxcr4* overexpression mutants.** (A-C,E-G) Whole-mount immunohistochemical analysis of limb skin with antibodies to the arterial EC marker connexin 40 (CX40, A-C, green; E-G, white; open arrows indicate remodeled arteries and white arrows indicate remodeled arterial branches) together with PECAM1 (A-C, red) in control littermates (A,E), *Cad5-BAC-Cre^ERT2^;Vhl^flox/flox^* (B,F) and *Cad5-BAC-Cre^ERT2^;ROSA-LSL-Cxcr4-hGFP* (C,G) embryos at E15.5. CX40^+^ remodeled arteries and arterial branches were nearly abolished in the skin vasculature of *Cad5-BAC-Cre^ERT2^;Vhl^flox/flox^* and *Cad5-BAC-Cre^ERT2^;ROSA-LSL-Cxcr4-hGFP* embryos (B,C,F,G). Scale bars: 100 µm. (D) Quantification of the percentage of CX40^+^ vessels in remodeled PECAM1^+^ blood vessels (*n*=17 for control, *n*=25 for *Cad5-BAC-Cre^ERT2^;Vhl^flox/flox^* and *n*=6 for *Cad5-BAC-Cre^ERT2^;ROSA-LSL-Cxcr4-hGFP*). Data are mean±s.d. Statistical *P* values were determined using the nonparametric Mann–Whitney test and a significance threshold of *P<*0.05 was considered to indicate a statistically significant reduction in arterial branches compared to control littermates (*P<0.0001 for *Cad5-BAC-Cre^ERT2^;Vhl^flox/flox^* and *P<0.0001 for *Cad5-BAC-Cre^ERT2^;ROSA-LSL-Cxcr4-hGFP* embryos). (H-J,L-N) Whole-mount immunohistochemical analysis of limb skin with antibodies to the venous and capillaries marker endomucin (EMCN, H-J, green; L-N, white; open arrowheads indicate remodeled veins and white or yellow arrowheads indicate remodeled venous branches) together with PECAM1 (red) in control littermates (H,L), *Cad5-BAC-Cre^ERT2^;Vhl^flox/flox^* (I,M) and *Cad5-BAC-Cre^ERT2^;ROSA-LSL-Cxcr4-hGFP* (J,N) embryos at E15.5. (K) Quantification of EMCN^+^ venous and capillary branch points (junctions per mm^2^) by AngioTool (*n*=9 for control, *n*=15 for *Cad5-BAC-Cre^ERT2^;Vhl^flox/flox^* and *n*=5 for *Cad5-BAC-Cre^ERT2^;ROSA-LSL-Cxcr4-hGFP*). Data are mean±s.d. Statistical *P* values were determined using the nonparametric Mann–Whitney test and a significance threshold of *P<*0.05 was considered to indicate a statistically significant reduction in venous and capillary branch points compared to control littermates (*P<0.0001 for *Cad5-BAC-Cre^ERT2^;Vhl^flox/flox^* and *P=0.0010 for *Cad5-BAC-Cre^ERT2^;ROSA-LSL-Cxcr4-hGFP* embryos).

We next investigated arterial and venous development in the skin vasculature of EC-specific *Vhl* deletion mutants and EC-specific *Cxcr4* overexpression mutants. To assess this, we examined the expression of the arterial marker connexin 40 (CX40), which is restricted to remodeled arteries and arterial branches (Fig. 4A and E: remodeled artery indicated by open arrows, remodeled arterial branches indicated by arrows) (Li et al., 2013; Mukouyama et al., 2005, 2002). CX40^+^ arterial branching was nearly abolished in both *Cad5-BAC-Cre^ERT2^;Vhl^flox/flox^* mutants and *Cad5-BAC-Cre^ERT2^;ROSA-LSL-Cxcr4-hGFP* mutants (Fig. 4A versus B, A versus C, E versus F, E versus G; D). We also examined the expression of the venous and capillary marker endomucin (EMCN), indicating the formation of remodeled veins and venous branches along with remodeled arteries and arterial branches, respectively: one arterial branch appeared to run alongside two venous branches (Fig. 4H,L, remodeled veins indicated by open arrowheads, remodeled venous branches indicated by arrowheads and remodeled arterial branch indicated by an arrow) (Li and Mukouyama, 2013; Kidoya et al., 2015). In both mutants, EMCN^+^ veins were presented (Fig. 4I and J, open arrowheads). However, EMCN^+^ venous branches showed disorganization and reduced complexity, along with a lack of arterial and venous co-branching (Fig. 4H versus I, H versus J, L versus M, L versus N; K). These data suggest that both EC-specific *Vhl* deletion mutants and EC-specific *Cxcr4* overexpression mutants lead to similar abnormalities in arterial and venous development during vascular network formation.

Vascular smooth muscle cell (VSMC) coverage in remodeled blood vessels is crucial for the formation of a highly branched vascular network and for maintaining vessel wall integrity (Bautch and Mukouyama, 2022; Li et al., 2013; Mukouyama et al., 2002). In control littermates at E15.5, αSMA^+^ VSMC coverage was observed in remodeled arteries and arterial branches (Fig. S8A,E: remodeled artery indicated by open arrows, remodeled arterial branches indicated by arrows). VSMC coverage was also observed in large-diameter veins (Fig. S8A,E, open arrowheads), although venous branches had limited VSMC coverage at this developmental stage (Fig. S8A,E, arrowheads). However, given that arterial branching was nearly abolished (Fig. 4A-G), few or no VSMC-covered arterial branches were observed in both mutants (Fig. S8A versus B, A versus C, E versus F, E versus G; D). Notably, increased VSMC coverage was observed in venous branches in both mutants (Fig. S8A versus B, A versus C, E versus F, E versus G; arrowheads; D). Additionally, aberrant VSMC coverage in enlarged capillaries was more pronounced in *Cad5-BAC-Cre^ERT2^;Vhl^flox/flox^* mutants than in *Cad5-BAC-Cre^ERT2^;ROSA-LSL-Cxcr4-hGFP* mutants (Fig. S8B versus C, F versus G), suggesting that EC-derived signals may induce abnormal VSMC differentiation and behavior in *Cad5-BAC-Cre^ERT2^;Vhl^flox/flox^* mutants. Consistent with this, conditional *Vhl* mutations lead to increased and abnormal VSMC coverage in postnatal retinal vasculature (Arreola et al., 2018).

Our previous studies have demonstrated that coronary vascular development is impaired in EC-specific *Cxcr4* overexpression mutants (Li et al., 2021). In this study, we further investigated whether ectopic CXCR4 expression in EC-specific *Vhl* deletion mutants also impacts coronary vascular development. Our whole-mount immunohistochemical analysis of the heart ventricle at E15.5 revealed that, in control littermates, the subepicardium of the dorsal ventricular surface displayed a highly branched EMCN^+^/PECAM1^+^ venous network, including three large-diameter venous branches in the dorsal surface (Fig. S9A and C, arrowheads). In contrast, the myocardium of the ventral ventricular wall displayed branched EMCN^−^/PECAM1^+^ capillaries along with large-diameter arterial branches (Fig. S9E and G, arrows). However, in *Cad5-BAC-Cre^ERT2^;Vhl^flox/flox^* mutants, the remodeled large-diameter venous and arterial coronary vasculature were almost undetectable (Fig. S9A versus B, C versus D, E versus F, G versus H). Combined, these findings suggest that EC-specific *Vhl* deletion leads to significant disruptions in both dermal and coronary vasculature.

### EC-specific *Vhl* deletion mutants and EC-specific *Cxcr4* overexpression mutants exhibit blood-filled lymphatic vascular phenotypes

**Fig. 5.**
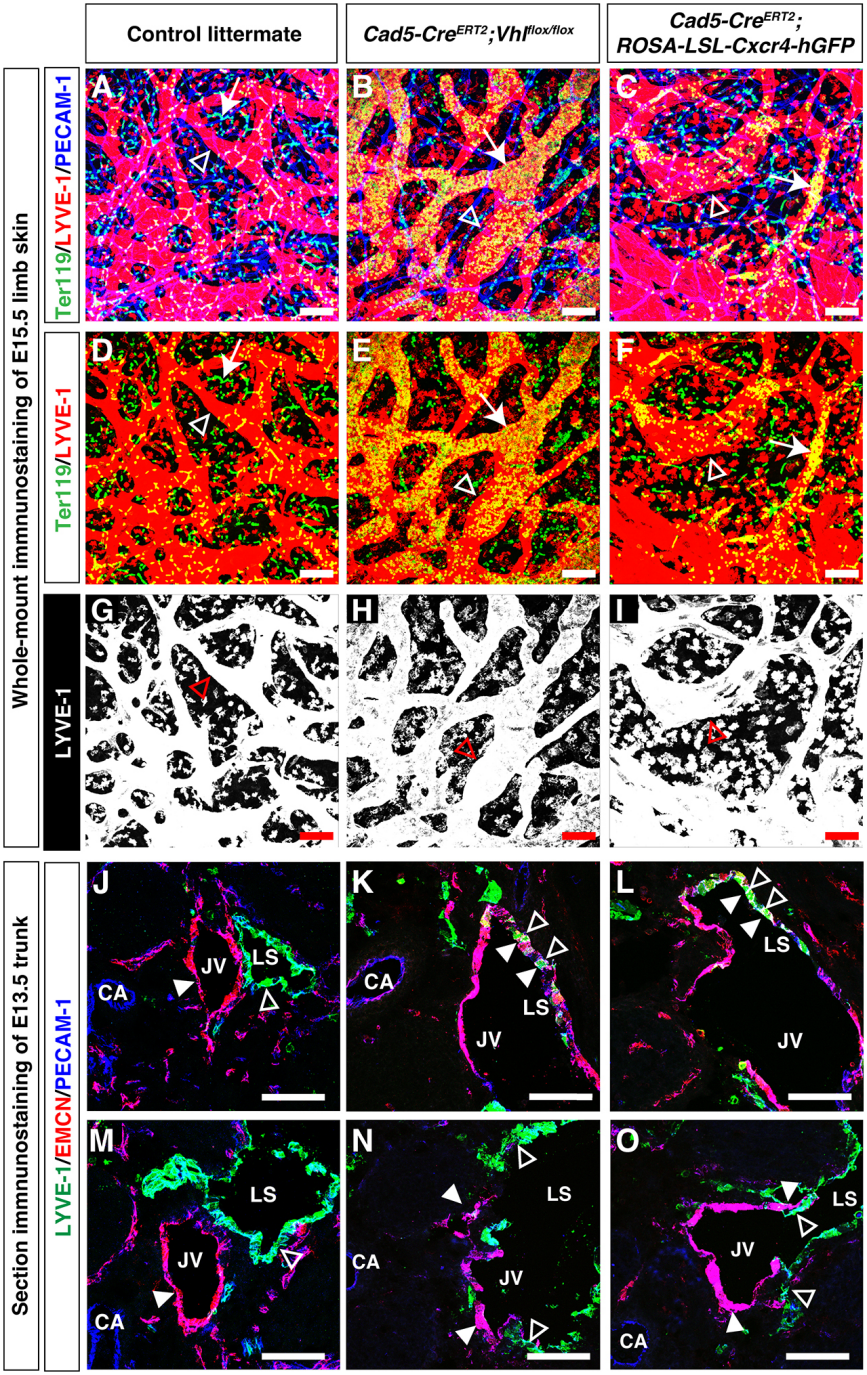
**Blood-filled lymphatic vasculature phenotype and incomplete separation of lymph sac from jugular veins in EC-specific *Vhl* deletion and EC-specific *Cxcr4* overexpression mutants.** (A-I) Whole-mount immunohistochemical analysis of limb skin with antibodies to the erythrocyte marker Ter119 (A-F, green) and lymphatic EC marker LYVE1 (A-F, red; G-I, white) together with PECAM1 (A-C, blue) in control littermates (A,D,G),*Cad5-BAC-Cre^ERT2^;Vhl^flox/flox^* (B,E,H) and *Cad5-BAC-Cre^ERT2^;ROSA-LSL-Cxcr4-hGFP* (C,F,I) embryos at E15.5. Open arrowheads indicate representative blood-filled LYVE1^+^ (PECAM1^weak^) lymphatic vessels. The LYVE1^+^/PECAM1^−^ round-shaped cells are tissue-resident macrophages. Ter119^+^ erythrocytes (arrows) were observed in PECAM1^+^ (LYVE1^−^) blood vessels, but not in LYVE1^+^ lymphatic vessels, in the control littermate (A,D,G). In contrast, *Cad5-BAC-Cre^ERT2^;Vhl^flox/flox^* (B,E) and *Cad5-BAC-Cre^ERT2^;ROSA-LSL-Cxcr4-hGFP* (C,F) mutants showed an accumulation of Ter119^+^ erythrocytes in LYVE1^+^ lymphatic vessels. Scale bars: 100 µm. (J-N) Section immunohistochemical analysis of E13.5 trunk of control littermates (J,M), *Cad5-BAC-Cre^ERT2^;Vhl^flox/flox^* (K,N) and *Cad5-BAC-Cre^ERT2^;ROSA-LSL-Cxcr4-hGFP* (L,O) embryos with antibodies to LYVE1 (green), EMCN (red) and PECAM1 (blue). White arrowheads indicate representative EMCN^+^/LYVE1^−^ jugular veins; open arrowheads indicate LYVE1^+^/EMCN^−^ lymph sacs in control littermates (J,M). In contrast, LYVE1^+^/EMCN^+^ vessels were observed in the lymph sac of *Cad5-BAC-Cre^ERT2^;Vhl^flox/flox^* (K) and *Cad5-BAC-Cre^ERT2^;ROSA-LSL-Cxcr4-hGFP* embryos (L). Additionally, EMCN^+^/LYVE1^−^ jugular veins and LYVE1^+^/EMCN^−^ lymph sac appeared fused in these mutants (N,O). Scale bars: 100 µm. LS, lymph sac; JV, jugular vein; CA, carotid artery.

Our previous studies have demonstrated that the hemorrhage-like phenotype and severe edema in the skin of *Cad5-BAC-Cre^ERT2^;ROSA-LSL-Cxcr4-hGFP* embryos result from blood-filled lymphatic vessels containing Ter119^+^ erythrocytes (Li et al., 2021). Overexpression of *Cxcr4* in ECs, but not lymphatic ECs (LECs), disrupted the separation of lymph sacs from jugular veins during development, leading to blood-filled lymphatic phenotypes. Thus, these phenotypes are not directly caused by *Cxcr4* overexpression in LECs (Li et al., 2021). Similarly, blood-filled LYVE1^+^ lymphatic vessels containing Ter119^+^ erythrocytes were observed in the skin of *Cad5-BAC-Cre^ERT2^;Vhl^flox/flox^* mutants and *Cad5-BAC-Cre^ERT2^;ROSA-LSL-Cxcr4-hGFP* mutants (Fig. 5A-I; A versus B, A versus C, D versus E, D versus F, open arrowheads). To determine whether *Vhl* deletion in LECs leads to a blood-filled lymphatic phenotype, we generated LEC-specific *Vhl* conditional knockout mice by crossing *Vhl-flox* mice with LEC-specific *Prox-1-Cre* driver mice, which delete the *Vhl* gene specifically in LECs. No visible abnormalities in the gross morphology and embryonic size were found in *Prox-1-Cre;Vhl^flox/flox^* mutants compared to control littermates at E15.5 (Fig. S10A,B). Furthermore, whole-mount immunohistochemical analysis revealed no Ter119^+^ erythrocytes in podoplanin (PDPN)^+^ lymphatic vessels in the skin of *Prox-1-Cre;Vhl^flox/flox^* mutants (Fig. S10C-H). These findings suggest that the defects in blood-filled lymphatic vasculature observed in EC-specific *Vhl* deletion mutants are not the primary defects.

In the trunk, EMCN^+^/LYVE1^+^/PECAM1^+^ ECs were present in incomplete separation of LYVE1^+^ lymph sacs from EMCN^+^ jugular veins (Fig. 5J versus K, J versus L; jugular vein indicated by arrowheads, lymph sac indicated by open arrowheads), while the fused lymph sac-jugular vein vessels were also observed in both *Cad5-BAC-Cre^ERT2^;Vhl^flox/flox^* mutants and *Cad5-BAC-Cre^ERT2^;ROSA-LSL-Cxcr4-hGFP* mutants (Fig. 5M versus N, M versus O: jugular vein indicated by arrowheads, lymph sac indicated by open arrowheads). Blood-lymphatic vascular separation is regulated by PDPN on LECs and platelet-dependent activation via the C-type lectin-like receptor 2 (CLEC2) (Bertozzi et al., 2010a,b; Mahamud et al., 2023; Uhrin et al., 2010). Thus, we examined the PDPN expression and platelet accumulations in EC-specific *Vhl* deletion mutants and EC-specific *Cxcr4* overexpression mutants. Our results revealed weak PDPN expression in LYVE1^+^ lymphatic endothelial cells in both *Cad5-BAC-Cre^ERT2^; Vhl^flox/flox^* mutants and *Cad5-BAC-Cre^ERT2^;ROSA-LSL-Cxcr4-hGFP* mutants compared to control littermates (Fig. S11A versus B and C, D versus E and F, G versus H and I, J versus K and L, open arrowheads). We also found that CD41^+^ platelet accumulation was found in the EMCN^+^ jugular veins in the control littermates, whereas there were significant fewer or no CD41^+^ platelets in EMCN^+^ jugular veins in both *Cad5-BAC-Cre^ERT2^;Vhl^flox/flox^* mutants and *Cad5-BAC-Cre^ERT2^;ROSA-LSL-Cxcr4-hGFP* mutants (Fig. S11J versus K and L, arrows). These findings suggest that the impaired blood-lymph separation in these mutants may be due to disrupted interactions between platelets and LECs.

It is important to note that the formation of the lymphovenous valves (LVVs) between the lymph sac and the cardinal vein prevents the backflow of blood into lymphatic vessels (Balint and Jakus, 2021; Mahamud et al., 2023). However, our results indicate no significant differences in the structure and number of LYVE1^+^/EMCN^−^/PECAM1^+^ LVVs among control littermates, *Cad5-BAC-Cre^ERT2^;Vhl^flox/flox^* mutants and *Cad5-BAC-Cre^ERT2^;ROSA-LSL-Cxcr4-hGFP* mutants (Fig. S12A versus B and C; E versus F and G; I versus J and K). Taken together, these findings suggest that blood-filled lymphatic phenotypes result from a failure of lymph sac separation from jugular vein, rather than LVV malformation, in both EC-specific *Vhl* deletion mutants and EC-specific *Cxcr4* overexpression mutants.

### The CXCR4 antagonist AMD3100 partially rescues vascular abnormalities in EC-specific *Vhl* deletion mutants

**Fig. 6.**
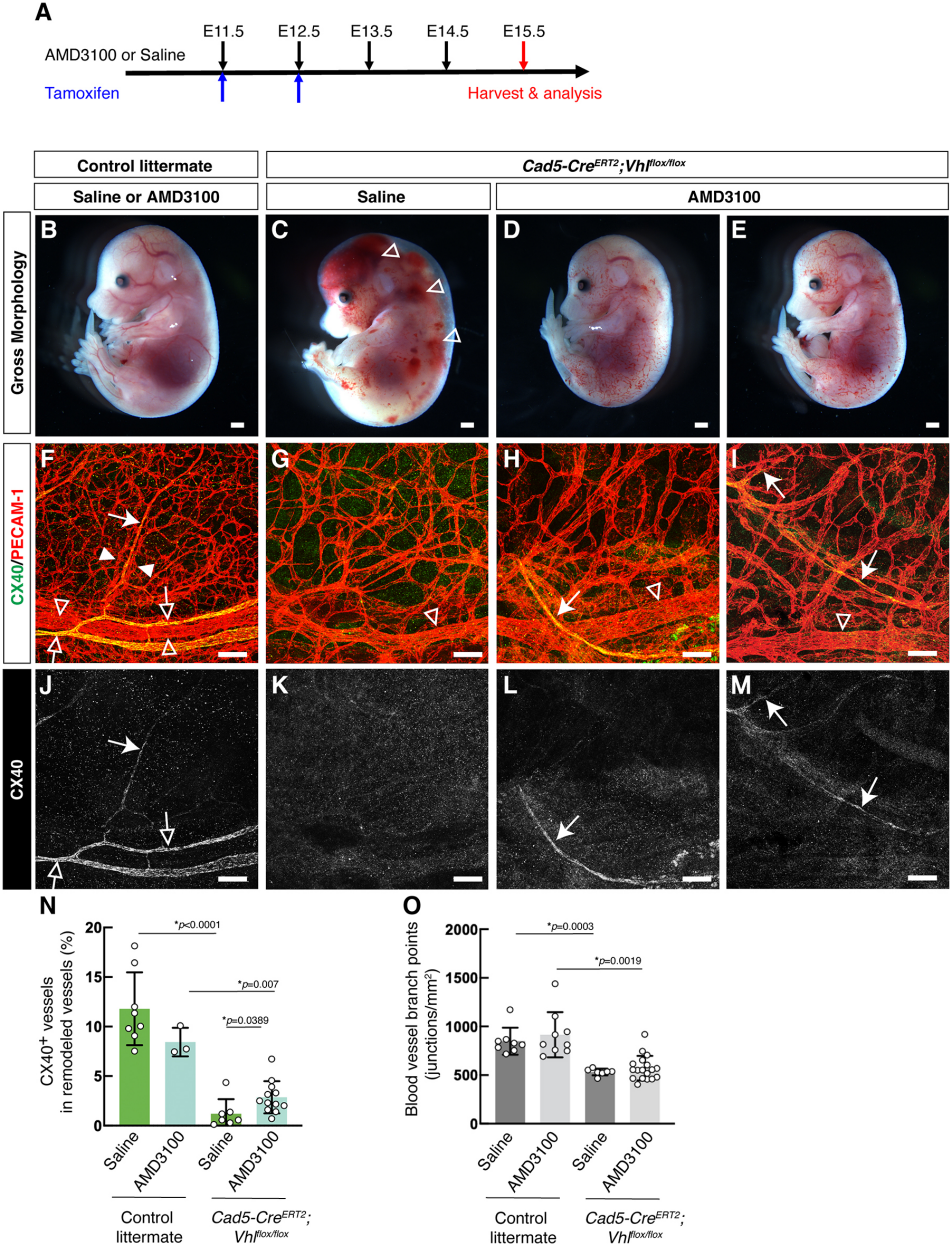
**The CXCR4 antagonist AMD3100 partially rescues vascular abnormalities in EC-specific *Vhl* deletion mutants.** (A) Diagram illustrating saline or AMD3100 administration to the EC-specific *Vhl* deletion embryos. Black arrows indicate time points for subcutaneous (SubQ) administration of saline (150 µl) or 40 mg/kg AMD3100 (150 µl) in pregnant mice. Blue arrows indicate the time points for intraperitoneal (IP) administration of 2 mg tamoxifen into pregnant mice. Red arrows indicate the time point for embryo harvesting and analysis. (B-E) Gross morphology of control littermate embryos at E15.5 from pregnant mice treated with either saline or AMD3100 (B) and *Cad5-BAC-Cre^ERT2^;Vhl^flox/flox^* embryos at E15.5 from pregnant mice treated with saline (C) or AMD3100 (D,E). Scale bars: 1 mm. Open arrowheads in C indicate a hemorrhage-like phenotype. (F-M) Whole-mount immunohistochemical analysis of limb skin with antibodies to CX40 (F-I, green; J-M, white; open arrows indicate remodeled arteries and white arrows indicate remodeled arterial branches) together with PECAM1 (F-I, red; open arrowheads indicate remodeled veins and white arrowheads indicate remodeled venous branches) in control littermate embryos at E15.5 from pregnant mice treated with saline or AMD3100 (F,J) and *Cad5-BAC-Cre^ERT2^;Vhl^flox/flox^* embryos at E15.5 from pregnant mice treated with saline (G,K) or AMD3100 (H,I,L,M). CX40^+^ remodeled arteries and arterial branches were nearly abolished in the skin vasculature of *Cad5-BAC-Cre^ERT2^;Vhl^flox/flox^* embryos from pregnant mice treated with saline (G,K), while some CX40^+^ arterial branches were observed in those treated with AMD3100 (H,I,L,M; arrows). Scale bars: 100 µm. (N) Quantification of the percentage of CX40^+^ vessels in remodeled PECAM1^+^ blood vessels (*n*=8 for control littermate embryos from pregnant mice treated with saline, *n*=3 for control littermate embryos treated with AMD3100, *n*=7 for *Cad5-BAC-Cre^ERT2^;Vhl^flox/flox^* embryos treated with saline and *n*=12 for *Cad5-BAC-Cre^ERT2^;Vhl^flox/flox^* embryos treated with AMD3100). Data are mean±s.d. Statistical *P* values were determined using the nonparametric Mann–Whitney test, and a significance threshold of *P<*0.05 was considered to indicate a statistically significant difference. The AMD3100 treatment significantly increased the percentage of CX40^+^ arterial branches (*P=0.0389) compared to the saline treatment in the skin vasculature of *Cad5-BAC-Cre^ERT2^;Vhl^flox/flox^* embryos. (O) Quantification of PECAM1^+^ blood vessel branch points (junctions per mm^2^) by AngioTool at E15.5 (*n*=8 for control littermate embryos from pregnant mice treated with saline, *n*=9 for control littermate embryos treated with AMD3100, *n*=7 for *Cad5-BAC-Cre^ERT2^;Vhl^flox/flox^* embryos treated with saline and *n*=18 for *Cad5-BAC-Cre^ERT2^;Vhl^flox/flox^* embryos treated with AMD3100). Data are mean±s.d. Statistical *P* values were determined using the nonparametric Mann–Whitney test and a significance threshold of *P<*0.05 was considered to indicate a statistically significant difference. PECAM1^+^ blood vessels branchpoints were not fully restored (*P=0.0019) in the skin vasculature of *Cad5-BAC-Cre^ERT2^;Vhl^flox/flox^* embryos treated with AMD3100 treatment compared to control littermates treated with AMD3100.

We aimed to determine whether the vascular abnormalities in EC-specific *Vhl* deletion mutants are directly caused by aberrant activation of CXCL12-CXCR4 signaling. Given that EC-specific *Cxcr4* deletion mutants exhibit similar vascular abnormalities to EC-specific *Cxcr4* overexpression mutants (Li et al., 2013, 2021), suggesting a critical window of appropriate CXCL12-CXCR4 signaling for proper vascular development, conducting an epistasis experiment by comparing vascular phenotypes between EC-specific *Vhl* deletion mutants and EC-specific *Vhl* and *Cxcr4* double-deletion mutants would be challenging. Therefore, we partially inactivated CXCL12-CXCR4 signaling by administrating the CXCR4 antagonist AMD3100 into pregnant mice subcutaneously (Fig. 6A). No significant morphological changes were observed in E15.5 control littermate embryos from pregnant mice treated with either saline or AMD3100 (Fig. 6B). In contrast, a hemorrhage-like phenotype and severe edema were observed in the skin of *Cad5-BAC-Cre^ERT2^;Vhl^flox/flox^* mutant embryos from pregnant mice treated with saline (Fig. 6C, open arrowheads). Notably, this hemorrhage-like phenotype and edema appeared to be alleviated in the skin of *Cad5-BAC-Cre^ERT2^;Vhl^flox/flox^* mutant embryos from pregnant mice treated with AMD3100 (Fig. 6C versus D, C versus E, open arrowheads). This improvement may be due to a reduction in the number of blood-filled lymphatic vessels in the skin of *Cad5-BAC-Cre^ERT2^;Vhl^flox/flox^* mutant embryos in pregnant mice following AMD3100 treatment (Fig. S13B versus C, E versus F, open arrowheads).

We next examined the effect of the CXCR4 antagonist AMD3100 on vascular network formation. No significant changes in arterial and venous development were observed in E15.5 control littermate embryos from pregnant mice treated with either saline or AMD3100 (Fig. 6F and J: arteries indicated by open arrows and arterial branch indicated by arrows; veins indicated by open arrowheads and venous branches indicated by arrowheads). In contrast, a lack of CX40^+^ arterial branching and abnormal venous development were observed in the skin of *Cad5-BAC-Cre^ERT2^;Vhl^flox/flox^* mutant embryos from pregnant mice treated with saline (Fig. 6G and K). Notably, CX40^+^ arterial branches, despite their abnormal branching, were observed in *Cad5-BAC-Cre^ERT2^;Vhl^flox/flox^* mutant embryos from pregnant mice treated with AMD3100 (Fig. 6G versus H, G versus I, K versus L, K versus M, arrows; N). Although the large-diameter vein in *Cad5-BAC-Cre^ERT2^;Vhl^flox/flox^* mutant embryos appeared to take on a more regular shape following AMD3100 treatment, the disorganized and reduced complexity of venous branches did not seem to be restored (Fig. 6G versus H, G versus I; O). Overall, AMD3100 treatment partially rescues vascular abnormalities in EC-specific *Vhl* mutants, including impaired arterial development and the presence of blood-filled lymphatics.

### *CXCR4* expression is upregulated in the tumor vasculature of *VHL*-related clear cell renal cell carcinomas

**Fig. 7.**
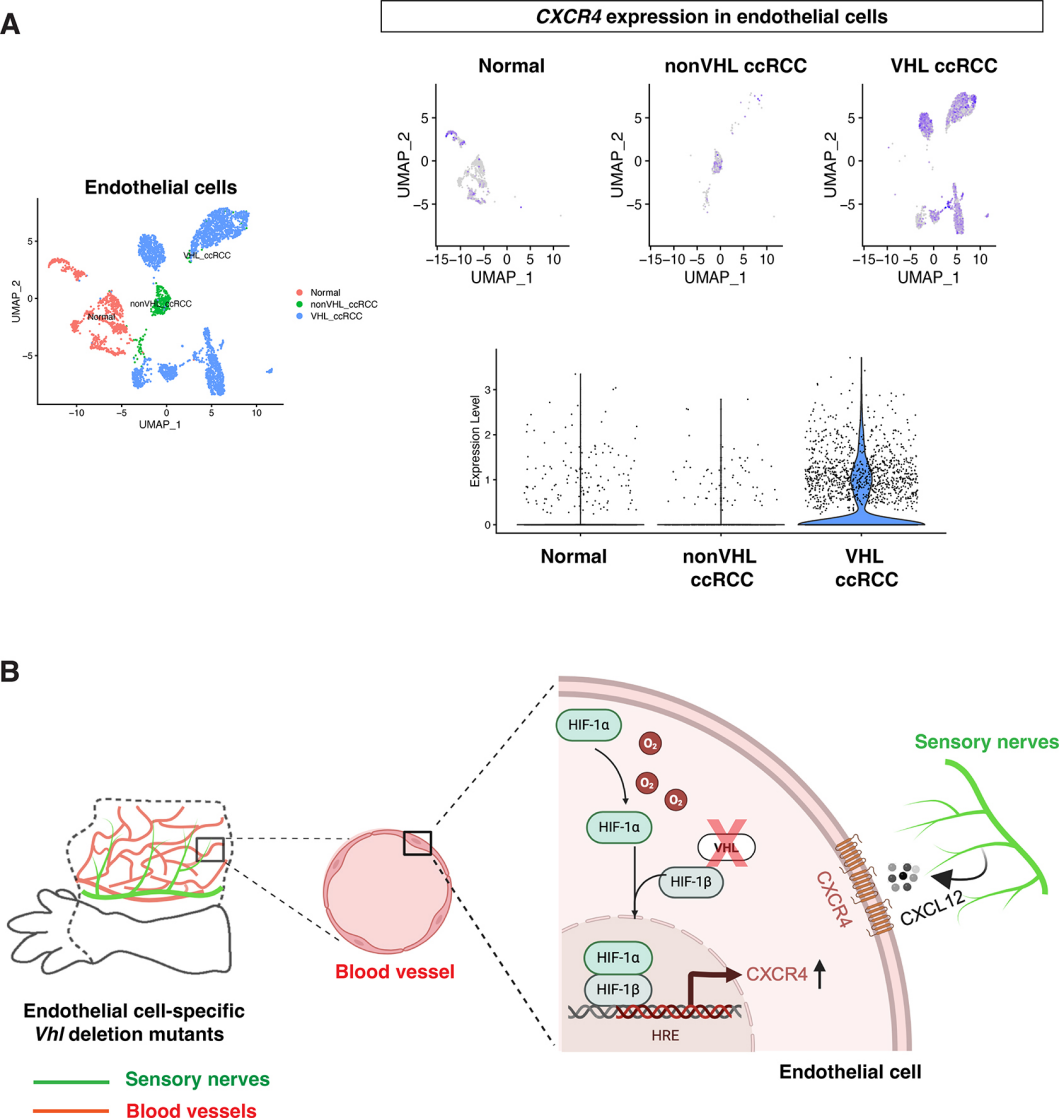
**Vascular *CXCR4* expression in individuals with VHL-related tumors.** (A) Left: UMAP plot of scRNA-seq data representing endothelial cell clusters in individuals from normal cortex/medulla, clear cell renal cell carcinoma (ccRCC) tumors without *VHL* mutations, and ccRCC tumors with *VHL* mutations. Right: UMAP plots and violin plots showing *CXCR4* expression in endothelial cells of individuals from normal cortex/medulla, ccRCC tumors without *VHL* mutations and ccRCC tumors with *VHL* mutations. There is increased *CXCR4* expression in ECs of ccRCC tumors with *VHL* mutations compared to ECs from other tumor or normal tissues. (B) Schematic illustration of endothelial CXCR4 upregulation in the skin vasculature of EC-specific *VHL* deletion mutants. *VHL* deletion leads to the constitutive stabilization of HIFs, resulting in ectopic upregulation of CXCR4 in ECs. In the developing skin vasculature model, sensory nerve-derived CXCL12 normally recruits CXCR4^+^ ECs to form arterial branches aligned with nerves during the remodeling of the capillary network into a branched, hierarchical vascular network (Bautch and Mukouyama, 2022; Li et al., 2013). However, the ectopic upregulation of CXCR4 in ECs leads to vascular abnormalities, driven by dysregulated CXCL12-CXCR4 signaling in EC-specific *Vhl* deletion mutants, as we previously observed in EC-specific *Cxcr4* overexpression mutants (Li et al., 2021). HRE, hypoxia-response element. Created in BioRender by Li, W., 2026. https://BioRender.com/ziz728n. This figure was sublicensed under CC-BY 4.0 terms.

VHL syndrome is characterized by highly vascularized tumor driven by dysregulated angiogenesis. To extend our findings to VHL syndrome in humans, we investigated *CXCR4* expression in VHL tumor vasculature. Previous studies show high levels of *CXCR4* transcripts in the VHL retinal hemangioblastomas (Liang et al., 2007; Zagzag et al., 2005). To further examine *CXCR4* expression in ECs, we utilized publicly available single-cell RNA-seq datasets from human clear cell renal cell carcinoma (ccRCC) subtypes and benign kidney tissue (Zhang et al., 2021). We extracted ECs from three datasets – benign adjacent kidney tissue (normal cortex/Medulla), ccRCC without *VHL* mutations and ccRCC with *VHL* mutations – and integrated them for analysis. Our results showed upregulation of *CXCR4* expression in ECs of ccRCC with *VHL* mutations compared to ECs of normal cortex/Medulla or ccRCC without *VHL* mutations (Fig. 7A, UMAP and violin plots). Additionally, *VEGFA* expression was upregulated in ECs of ccRCC with *VHL* mutations compared to ECs of normal cortex/medulla or ccRCC without *VHL* mutations (Fig. S14, UMAP and violin plots). These findings suggest that *VHL* mutations influence both *CXCR4* and *VEGF* expression in ccRCC ECs, contributing to abnormal angiogenesis.

## DISCUSSION

In this study, we investigated the cell-autonomous role of VHL in ECs using a conditional *Vhl* deletion during angiogenesis. In the developing skin vasculature model, EC-specific *Vhl* deletion mutants exhibited abnormal vascular network formation, including impaired arterial and venous development, as well as the formation of blood-filled lymphatics. Mechanistically, EC-specific *Vhl* deletion resulted in ectopic upregulation of CXCR4. Expression of *Cxcr4* is activated by HIFs, which are stabilized in the absence of VHL (Fig. 7B). In the skin vasculature, sensory nerve-derived CXCL12 normally recruits CXCR4^+^ ECs to form arterial branches aligned with nerves during the remodeling of the capillary network into a branched, hierarchical vascular network (Bautch and Mukouyama, 2022; Li et al., 2013). However, the ectopic upregulation of CXCR4 in ECs leads to vascular abnormalities, driven by dysregulated CXCL12-CXCR4 signaling (Li et al., 2021). Consistent with this, both EC-specific *Vhl* deletion mutants and EC-specific *Cxcr4* overexpression mutants displayed similar vascular abnormalities in arterial and venous development during vascular network formation. Moreover, the CXCR4 antagonist AMD3100 treatment partially rescues these vascular abnormalities in EC-specific *Vhl* deletion mutants. Taken together, our findings suggest that EC-specific *Vhl* deletion mutation causes vascular abnormalities through ectopic activation of HIF-CXCR4 signaling axis (Fig. 7B).

This study focused on the phenotypic and mechanistic analysis of the HIF-CXCR4 signaling axis in ECs during vascular development. We began by conducting RT-qPCR analysis of mRNA expression for HIF-target genes related to vascular development (Fig. S2). We included both previously reported HIF-target metabolic genes and angiogenic genes in our analysis; however, none of the metabolic genes showed significant enhancement and only a few angiogenic genes (*Adm*, *Cxcr4* and *Vegfa*) were upregulated in ECs from the embryonic skins of EC-specific *Vhl* deletion mutant mouse embryos. In our *in vitro* model using HUVECs, significant changes in the expression of *ALDOA*, *GLUT1* (*SLC2A1*) and *HK2* within the metabolic gene set were observed with *VHL* knockdown (Figs S15 and S16). Nonetheless, only *CXCR4* and *VEGFA* within the angiogenic gene set were upregulated in these cells. A comprehensive gene expression profiling and comparison between control littermates and EC-specific *VHL* deletion mutants could provide valuable insights into the landscape of HIF-target metabolic and angiogenic genes in ECs during vascular development.

We conducted further investigation to determine whether VHL loss leads to the upregulation of CXCR4 expression at the protein level through HIFs. Since the VHL ubiquitin ligase is responsible for the oxygen-dependent ubiquitylation and degradation of both HIF1A and HIF2A proteins, we found that *VHL* knockdown HUVECs resulted in increased CXCR4 expression compared to control siRNA-treated HUVECs, and *VHL/HIF1A/HIF2A* triple-knockdown HUVECs showed a reduction of CXCR4 expression compared to *VHL* knockdown HUVECs (Fig. S15). On the other hand, HIF1B, the β subunit of HIF1, is constitutively expressed and not regulated by the VHL ubiquitin ligase. HIF1B forms heterodimers with either HIF1A or HIF2A. Consequently, *VHL/HIF1A/HIF1B* triple-knockdown HUVECs also showed a reduction of *CXCR4* expression compared to *VHL* knockdown HUVECs (Fig. S16). Interestingly, *VHL/HIF1A/HIF1B* triple-knockdown HUVECs also showed a reduction of *VEGFA* expression compared to *VHL* knockdown HUVECs, while *VHL/HIF1A/HIF2A* triple-knockdown HUVECs did not show this effect (Figs S14 and S15). *In vivo* epistasis mouse experiments comparing EC-specific *Vhl* deletion mutants with EC-specific *Vhl/Hif1a/Hif2a* triple-deletion mutants or EC-specific *Vhl/Hif1a/Hif1b* triple-deletion mutants would provide compelling mechanistic evidence that EC-specific *Vhl* deletion mutation causes the ectopic activation of HIF-CXCR4 and HIF-VEGFA signaling axis.

As CXCL12-CXCR4 signaling is crucial for sensory nerve-mediated arterial branching in the developing skin vasculature, it also plays a key role in coronary branching. Cardiomyocytes expressing CXCL12 guide a subset of CXCR4^+^ endocardium-derived ECs to form arteries (D'Amato et al., 2022). However, an unanswered question is how does the excess CXCL12-CXCR4 signaling impair proper arterial branching? Our previous *in vitro* and *in vivo* studies demonstrated that while VEGFA-VEGFR2/NRP1 signaling is essential for arterial differentiation, CXCL12-CXCR4 signaling primarily regulates arterial morphogenesis, rather than differentiation (Li et al., 2013). CXCR4^+^ ECs in the capillary network appear to be pre-specified arterial ECs, with sensory nerve-derived CXCL12 recruiting these CXCR4^+^ ECs and sensory nerve-derived VEGFA promoting arterial differentiation. Since both EC-specific *Vhl* deletion mutants and EC-specific *Cxcr4* overexpression mutants show CXCR4 expression in most capillary ECs, this ectopic expression of CXCR4 may disrupt the selective recruitment of arterial ECs, resulting in impaired nerve-vessel alignment and arterial morphogenesis.

Venous branches develop in alignment with arterial branches in the developing skin vasculature, a process regulated by arterial EC-derived factor apelin, which directs venous branching via its receptor APJ on venous ECs (Kidoya et al., 2015). In the absence of arterial branches, venous branching is impaired, although VSMC-covered large-diameter veins can still form. Furthermore, since venous ECs do not naturally express CXCR4, ectopic CXCR4 expression in these cells may disrupt proper venous network formation, resulting in immature vasculature.

VEGFA is expressed by capillary ECs in the developing skin vasculature. Interestingly, in EC-specific *Vhl* deletion mutants, there is a significant increase in *Vegfa* mRNA expression, but no corresponding change in VEGFA protein levels in capillary ECs. This observation may suggest a potential post-translational regulation of VEGFA restores protein levels and autocrine signaling in these mutants. Previous studies in EC-specific deletion of *Vegfa* demonstrated that autocrine VEGFA is important for maintaining vascular integrity and cellular viability (Domigan et al., 2015; Lee et al., 2007). However, the mechanisms that control VEGFA expression in ECs remain unclear. Additionally, it is still uncertain whether the upregulation of VEGFA by ECs affects their properties in VHL-related tumor vasculature, indicating a need for further investigation.

Both LEC-specific *Vhl* deletion mutants (Fig. S10) and LEC-specific *Cxcr4* overexpression mutants (Li et al., 2021) exhibit normal lymphatic vascular development and proper blood-lymphatic separation, suggesting that the lymphatic abnormalities observed in EC-specific *Vhl* deletion mutants and EC-specific *Cxcr4* overexpression mutants are likely caused by secondary defects resulting from the ectopic activation of HIF-CXCR4 signaling axis in ECs. Interestingly, while impaired blood-lymphatic separation was observed, more anterior to the LVVs, in these mutants (the data are presented on p. 17 in the peer review history), the LVVs appear normal. These findings suggest that in these mutants, there is a distinct contribution of ECs with ectopically activated HIF-CXCR4 signaling to LVV formation and blood-lymphatic separation.

Consistent with our findings, both *VEGFA* and *CXCR4* expression were upregulated in ECs from individuals with VHL-related ccRCC. Since VHL regulates HIF-responsive pro-angiogenic genes such as *VEGFA* and *CXCR4*, and the response to VEGFA inhibitors can vary in VHL-related ccRCC, a combined therapeutic approach targeting both VEGFA and CXCR4 could be more effective for individuals with VHL syndrome, as preclinical experiments suggest that the combination of VEGFA and CXCR4 inhibitors can help prevent the growth of certain tumor, such as gliomas (Barone et al., 2014). Similarly, HIF2α inhibitors for targeting the HIF-VEGFA and HIF-CXCR4 axis have shown efficacy in reducing tumor vascularity and slowing disease progression in VHL-related ccRCC (Choueiri and Kaelin, 2020). Further research into cell type-specific mechanisms within the oxygen-sensing pathway may lead to significant discoveries of potential new targets for individuals with VHL-related tumors.

## MATERIALS AND METHODS

### Mice

All animal procedures were approved by the National Heart, Lung, and Blood Institute (NHLBI) Animal Care and Use Committee in accordance with NIH research guidelines for the care and use of laboratory animals. The following mice were used in this study: *Vhl^flox/flox^* mice (Haase et al., 2001; the Jackson Laboratory, 012933), *Rosa26-loxP-STOP-loxP-Cxcr4-hGFP* mice (Li et al., 2021), *Cad5-BAC-Cre^ERT2^* mice (Okabe et al., 2014) and *Prox1-BAC-Cre* mice [generated by Gene Expression Nervous System Atlas (GENSAT) Project at Rockefeller University and were commercially available from MMRRC#036644-UCD]. The *Cre*-mediated excision was induced by administrating tamoxifen (Millipore-Sigma, T-5648) by intraperitoneal injection. In mice rescue experiments, saline or AMD3100 (Selleckchem, S3013) were injected subcutaneously every day from E11.5 to E14.5 and analyzed at E15.5.

### Cell culture

Human umbilical vein endothelial cells (HUVECs, Lonza, C2519A) were used in culture study. Gene knockdown experiments were achieved by transfecting predesigned gene targeting mission esiRNAs (Millipore-Sigma, Mission siRNA Universal Negative control, SIC001; *HIF1A*, EHU151981; *HIF1B*, EHU083391; *HIF2A*, EHU008751; *VHL*, EHU074571) into HUVECs using X-tremeGENE kit (Roche, 04476093001) following by the manufacturer's protocol. After 48-72 h transfection, cells underwent RT-qPCR or flow cytometry to examine knockdown efficiency or CXCR4 expression, respectively.

### RNA isolation and reverse transcription-quantitative PCR

Total RNA was extract from the cultured HUVECs by RNeasy mini kit (Qiagen, 74104) according to the manufacturer's instructions. Genomic DNA was eliminated with DNase I (ThermoFisher Scientific, AM2222) and RNase inhibitor (NEB, M0307S) treatment at 37°C for 20 min. cDNA was reverse-transcribed from total RNA using ReverTra Ace qPCR RT Master Mix (Toyobo, FSQ-201S). Reverse transcription-quantitative PCR (RT-qPCR) was performed with Thunderbird Next SYBR qPCR Mix (Toyobo, QPX-201) on LightCycler 96 (Roche) equipment. The knockdown efficiency of the *HIF1A*, *HIF1B*, *HIF2A* and *VHL* genes was assessed by comparing the fold changes in relative mRNA levels between *VHL* knockdown, *VHL/HIF1A/1B* or *VHL/HIF1A/2A* triple-knockdown and control knockdown in HUVECs, normalized to human *GAPDH.* The relative mRNA levels of HIF-target angiogenic genes in EC-specific *VHL* mutant ECs compared to control littermate ECs were presented as fold changes relative to controls, normalized to mouse *Actb*. Primer sequences used for RT-qPCR are in Table S1.

### Fluorescence-activated cell sorting

Forelimb skins were peeled off from E13.5 embryos of control littermates or *Cad-BAC-Cre^ERT2^;Vhl^flox/flox^* mice in HBSS (Gibco, 14025) and dissociated by digestion with 0.1% type I collagenase (Worthington, LS004194), 0.3% Dispase (Gibco,17105-041), 0.05% deoxyribonuclease type 1 (DNase 1; Millipore-Sigma,D4527) and 5% fetal bovine serum (FBS, Hyclone,SH3007) in L15 medium (ThermoFisher Scientific, 21083) at 37°C for 1 h. The dissociated cells were filtered through 100 μm filters and washed with fluorescence-activated cell sorting (FACS) buffer (1% BSA, Millipore-Sigma, A-9418; 0.1 M HEPES, ThermoFisher Scientific, 21083-027; 1x Pen-Strep, Gibco, 15140; and 0.025% DNase I, in L15 medium). Cells were then incubated with FITC-conjugated anti-PECAM1 antibody (BD Biosciences, 553372, 1:50) to detect ECs. To eliminate erythrocytes and myeloid cells, the dissociated skin cells were incubated with APC-conjugated anti-Ter119 (eBiosciences, 17-5921-83, 1:100) and anti-CD45 (eBiosciences, 17-0451-83, 1:100) together with FITC-conjugated anti-PECAM1 antibody for 30 min on ice. Cell viability was assessed using 7-aminoactinomycin D (7AAD; ThermoFisher Scientific, A1310). The CD45^−^Ter119^−^/PECAM1^+^ endothelial cells were sorted using BD FACSAria-IIu cell sorter with BDFACSDiva software (BD Biosciences).

### Flow cytometry analysis

Transfected HUVECs were harvested with 0.25% trypsin-EDTA (Gibco, 25200-056) and flow cytometry was carried out following a modified procedure from that previously reported (Mukouyama et al., 2002). Briefly, the harvested HUVECs were filtered through 100 μm filters and washed with FACS buffer. Cells were then incubated with PE-conjugated rat anti-human CD184 (BD Biosciences, 561734, 1:50) for 30 min on ice to detect human CXCR4 expression. Cell viability was assessed using 7AAD. Flow cytometry analysis was performed with a FACS Aria II SORP instrument (BD Biosciences).

### Whole-mount immunohistochemistry

Staining was performed essentially as described previously (Li and Mukouyama, 2011). In brief, embryonic forelimb skins and hearts were collected and fixed by 4% paraformaldehyde at 4°C overnight. After being washed in PBS the next day, the samples were incubated with primary antibodies in PBS-Triton X-100 solution (Millipore-Sigma, T9284; 0.2%) with 10% heat inactivated goat serum (ThermoFisher Scientific, 16210-072) buffer at 4°C overnight. The primary antibodies used were rabbit anti-CXCR4 antibody (Biotrend, CXC-4000-RM-500; 1:10) to detect CXCR4; Armenian hamster anti-CD31 (Millipore-Sigma, MAB1398Z; 1:200) to detect ECs; rabbit anti-ERG (Abcam, ab92513, 1:500) to detect endothelial cell nuclei; rabbit anti-Cx40 (Alpha DiagnosticInternational, CX40-A; 1:200) as an arterial endothelial cell marker; rat anti-endomucin antibody (EMCN, Santa Cruz Biotechnology, sc-65495; 1:500) as a venous endothelial cell marker; rabbit anti-LYVE1 antibody (Abcam, ab14917; 1:200), rat anti-LYVE1 antibody (MBL, D225-3 1:200) and Syrian Hamster anti-Podopanin antibody (PDPN; Biolegend, 127410, 1:100) to detect lymphatic endothelial cells; rabbit anti-Collagen Type IV antibody (Col IV, Bio-Rad, 2150-1470, 1:400) to detect cell basal membrane to define vascular pruning; mouse anti-neuron specific class III β-tubulin antibody (Tuj1, Biolegend, 801202, 1:500); rat anti-Ter119-FITC antibody (ThermoFisher Scientific, 11-5921-82; 1:250) to detect erythrocytes; Cy3-conjugated anti-αSMA antibody (clone1A4, Millipore-Sigma, C6198;1:500) to detect smooth muscle cells; and goat anti-VEGF antibody (R&D, AF-493; 1:20) to detect VEGF expression. For immunofluorescence detection, either Cy3-, Alexa-488-, Alexa-568- or Alexa-647-conjugated secondary antibodies (Jackson ImmunoResearch or ThermoFisher Scientific, 1:250, 1 h at room temperature) were used. Details of the secondary antibodies used for the staining are provided in Table S2. All stained samples were mounted with ProLong Gold Antifade Mounting solution (ThermoFisher Scientific, P36934). All confocal microscopy was carried out on a Leica TCS SP5 confocal microscope. Quantification of the percentage of CX40^+^ vessels area, VSMC coverage and blood vessel branch points were analyzed with AngioTool (Zudaire et al., 2011). The unit was calculated with corrected total cell fluorescence (CTCF) as the mean fluorescence value after subtracting the background signals. Number of embryos or biological samples analyzed is indicated by *n* in the figure legends.

### Paraffin section immunohistochemistry

Embryos were fixed with 10% neutral buffered formalin (NBF, Millipore-Sigma, HT501128) for at least 24 h at room temperature; 5 μm thick paraffin wax-embedded sections were rehydrated and antigens were retrieved by incubation in target retrieval solution (DAKO, S1699) for 15 min followed by permeabilization in 0.5% Triton X-100/PBS for 10 min at room temperature. Sections were blocked with 10% goat serum in PBS-T (0.2% Triton X-100) and incubated with rabbit anti-mouse HIF1α antibody (Novus Biologicals, NB100-479; 1:100) overnight at 4°C. For immunofluorescent detection of HIF1α staining, sections were stained with goat anti-rabbit HRP 2nd antibody (PerkinElmer, NEF812001EA; 1:250) for 2 h at room temperature the following day, then incubated with opal 520 reagent (AKOYA, op-001001;1:200) for 15 min at room temperature. For co-staining nuclear of ECs, HIF1α antibody-stained sections were incubated with rabbit anti-mouse ERG antibody overnight at 4°C by following the antigen retrieval process steps above. For immunofluorescent detection of ERG staining, goat anti-rabbit Alexa-594-conjugated secondary antibody (Jackson ImmunoResearch, 111-587-003;1:250) was used. All confocal microscopy was carried out on a Leica TCS SP5 confocal microscope.

### Frozen section immunohistochemistry

Staining was performed essentially as described previously (Li et al., 2013). Embryos were fixed with 4% paraformaldehyde/PBS at 4°C overnight, sunk in 30% sucrose/PBS at 4°C and then embedded in OCT compound. Embryos were cryosectioned at 12 μm and collected on pre-cleaned slides. Staining was performed using Armenian hamster anti-CD31 antibody to detect ECs; rabbit anti-LYVE1 antibody, rat anti-LYVE1 antibody and Syrian Hamster anti-Podopanin antibody (PDPN; Biolegend, 127410, 1:100) to detect LECs; rat anti-CD41 (BD pharmingen, 561850, 1:100) to detect platelets; and rat anti-Ter119-FITC antibody to detect erythrocytes. For immunofluorescent detection, either Alexa- 488-, Alexa-568-, Alexa-647 or Cy3- conjugated secondary antibodies (Jackson ImmunoResearch or ThermoFisher Scientific, 1:250) were used. Details of the secondary antibodies used for the staining are provided in Table S2. All confocal microscopy was carried out on a Leica TCS SP5 confocal microscope.

### Single-cell RNA-sequencing data analysis

Publicly available anonymized single-cell gene expression data (Zhang et al., 2021) were extracted from National Center for Biotechnology Information (NCBI) Gene Expression Omnibus (accession number GSE159115). Analysis was performed using the R package Seurat. All datasets except for classical chromophobe RCC (chRCC) were merged; the top variable genes were identified using the Seurat implementation of FindVariableGenes, and the merged datasets were then integrated using the RunFastMNN function (Haghverdi et al., 2018) from the SeuratWrappers R package. Using the deposited metadata, the endothelial cell population was subset from the integrated dataset. Subsequently, principal component analysis was performed for dimensional reduction. Uniform manifold approximation and projection (UMAP) was then applied (dims=1:30). UMAP plots and violin plots were visualized using Seurat.

### Statistical analysis

Statistical significance of samples was assessed using the nonparametric test as indicated in the figure legends. *P*<0.05 was considered as significant difference between samples. Values are presented as mean±s.d. All statistical analysis was performed in GraphPad Prism 10 software.

## Supplementary Material



10.1242/develop.204519_sup1 Supplementary information
