# Supplementary material for: Endothelial von Hippel-Lindau gene deletion causes abnormal blood and lymphatic vasculature through ectopic activation of the HIF-CXCR4 axis
Source: Development. 2026 May 18;153(10):dev204519. doi: 10.1242/dev.204519 (PMC13282565; doi:10.1242/dev.204519)
Supplement: Supplementary information [file develop-153-204519-s1.pdf]

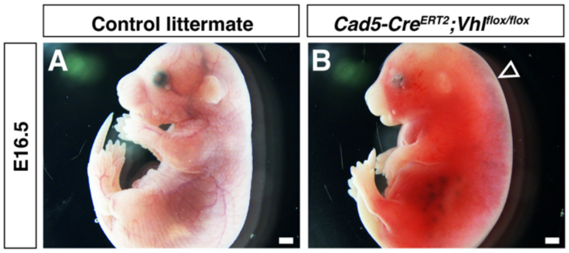

Fig. S1. Gross morphology of control littermate and EC-specific *Vhl* deletion embryos at E16.5.

Gross morphology of control littermates (A) and EC-specific *Vhl* deletion **e**mbryos (B) collected from the same litter and photographed at the same magnification at E16.5) are shown. Edema and hemorrhage-like phenotype was found in EC-specific *Vhl* deletion embryos at E16.5. Open arrowhead indicates edema phenotype in EC-specific *Vhl* deletion embryos. Scale bars represent 1mm.

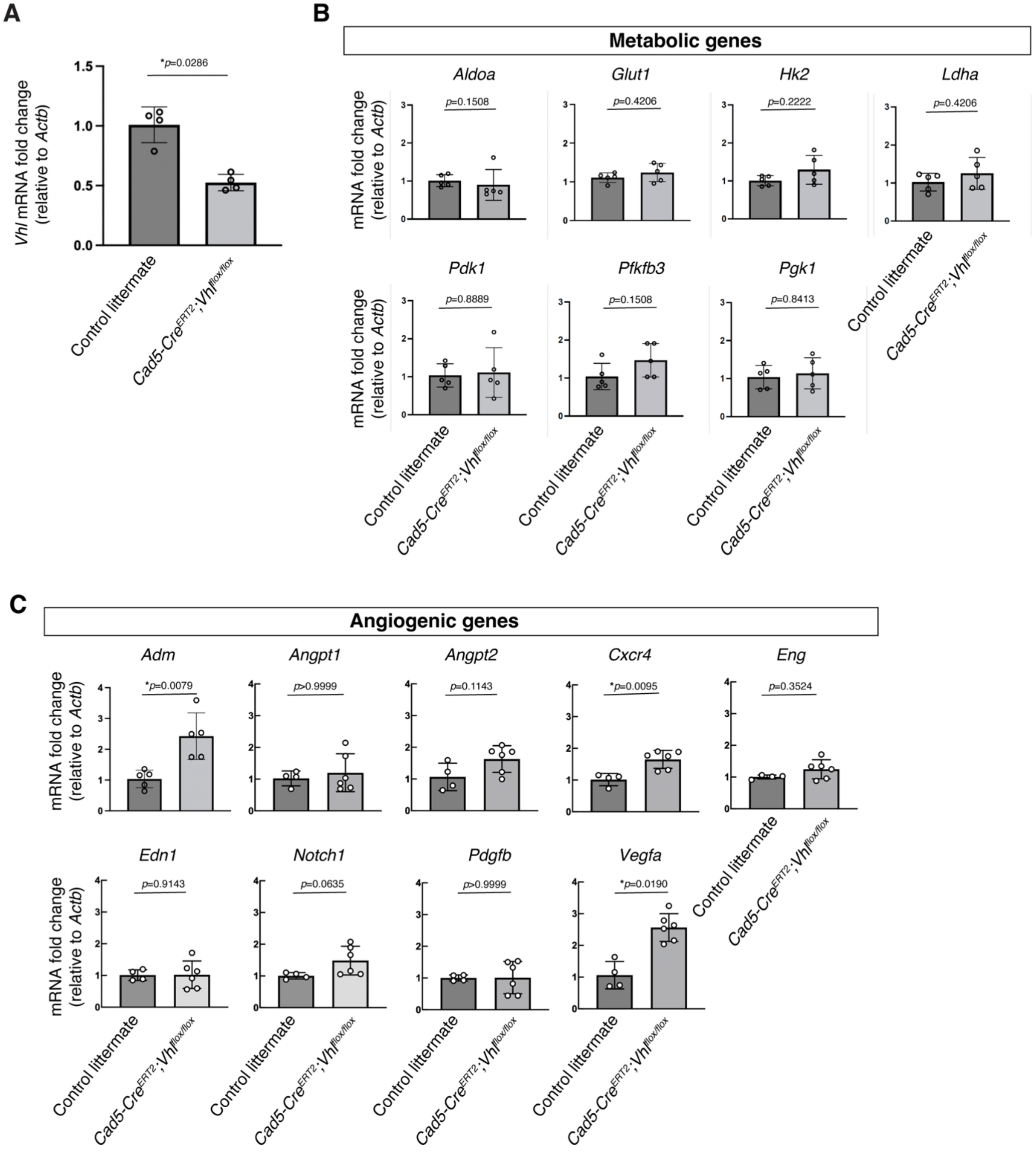

Fig. S2. *Vhl* deletion efficiency and the expression of HIF-targeted metabolic and angiogenic genes in ECs from EC-specific *Vhl* deletion mutants

(A-C) The fold changes in relative mRNA expression of *Vhl* gene (A), selective HIF-targeted metabolic genes (B) and angiogenic genes (C) were analyzed using RT-qPCR in fluorescence-activated cell sorting (FACS)-isolated endothelial cells (ECs) from the skin of control littermates (n=4) and *Cad5-BAC-Cre*^{ERT2};*Vhl*$^{flox/flox}$ (n=4) embryos at E13.5. Bars represent mean ± SD; Statistical *p* values were determined using the nonparametric

Development • Supplementary information

Mann-Whitney test and a significant threshold of $p<0.05$ was considered as statistically significant differences. Note that the mRNA expression level of *Vhl* ($p=0.0286$) was significantly decreased in ECs from *Cad5-BAC-Cre*^{ERT2}*;Vhl*$^{flox/flox}$ embryos compared to control littermates. This reduction is likely due to a mixture of ECs in which CreERT2 has removed both floxed alleles, only one allele, or none. The mRNA expression level of *Adm* (p=0.0079), *Cxcr4* ($p=0.0095$) and *Vegfa* ($p=0.0190$) were significantly increased in ECs from *Cad5-BAC-Cre*^{ERT2}*;Vhl*$^{flox/flox}$ embryos compared to control littermates. Asterisk indicates $p<0.05$.

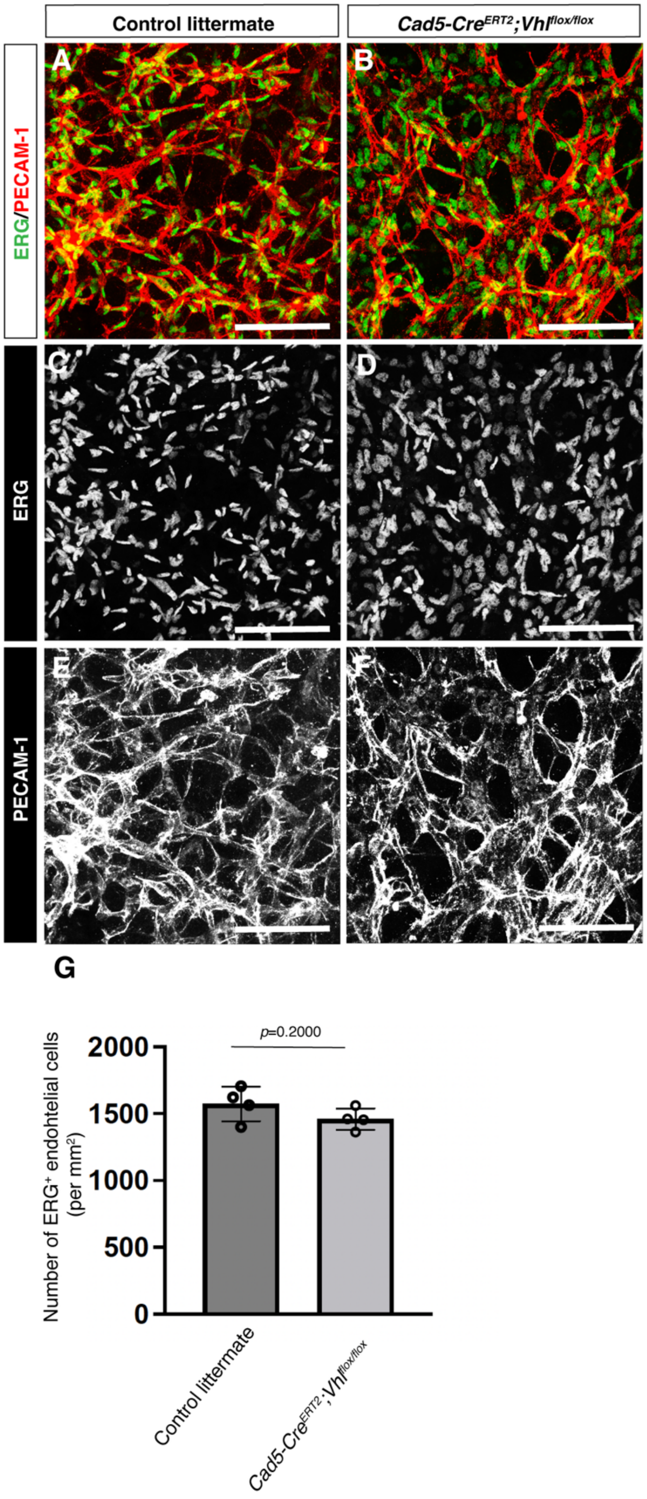

Control littermate
Cad5-CreERT2;Vhlflox/flox
ERG/PECAM-1
ERG
PECAM-1
A
B
C
D
E
F
G
p=0.2000
Number of ERG+ endohtelial cells (per mm2)
2000
1500
1000
500
0
Control littermate
Cad5-CreERT2;Vhlflox/flox

Fig. S3. No significant change in the number of ECs between EC-specific *Vhl* deletion mutants and control littermates

(A-F) Whole-mount immunohistochemical analysis of limb skin with antibodies to the EC nuclei marker ERG (A and B, green; C and D, white), together with PECAM-1 (A and B, red; E and F, white) in control littermate (A, C, and E) and *Cad5-BAC-CreER*^{T2}*;Vhl*$^{flox/flox}$ embryos (B, D, and F) embryos at E13.5. Scale bars represent 100 μm. (G) Quantification of the number of ERG^{+} ECs in control littermate and *Cad5-BAC-CreER*^{T2}*;Vhl*$^{flox/flox}$ embryos at E13.5 (n=4 per genotype) using Fiji software. Bars represent mean ± SD. Statistical *p* value was determined using the nonparametric Mann-Whitney test and a significant threshold of $p<0.05$ was considered as statistically significant differences.

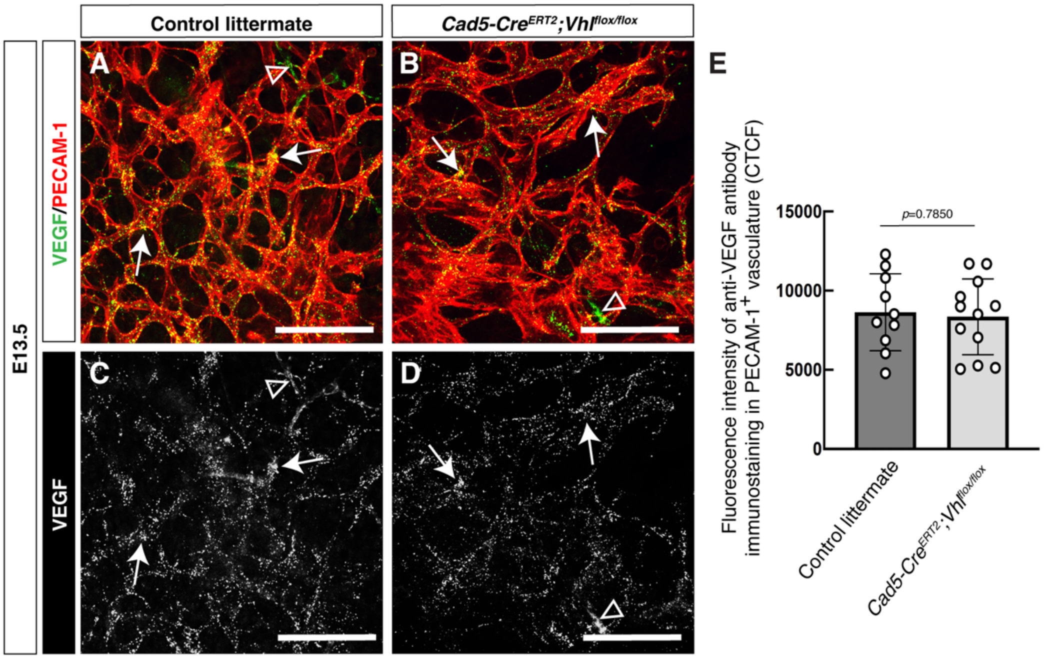

Fig. S4. Vascular VEGFA expression in the skin of EC-specific *Vhl* deletion mutants

(A-D) Whole-mount immunohistochemical analysis of limb skin with antibodies to VEGF (A-B, green; C-D, white), together with PECAM-1 (A-B, red) in control littermates (A and C) and *Cad5-BAC-Cre^{ERT2};Vhl$^{flox/flox}$* (B and D) embryos at E13.5 are shown. Arrows indicate representative VEGF-expressing ECs in the capillary network. Open arrowheads indicate VEGF-expressing peripheral nerves. Scale bars represent 100 μm. (E) Quantification of VEGF expression in the capillary network in control littermates and *Cad5-BAC-Cre^{ERT2};Vhl$^{flox/flox}$* embryos. The data were accomplished by measuring VEGF fluorescence intensity using Fiji software and the unit is calculated with corrected total cell fluorescence (CTCF) (n=10 for control littermates, n=12 for *Cad5-BAC-Cre^{ERT2};Vhl$^{flox/flox}$* embryos). Bars represent mean ± SD. Statistical *p* values were determined using the nonparametric Mann-Whitney test and a significant threshold of *p*<0.05 was considered as statistically significant differences. There are no statistical differences (p=0.7850) in VEGF expression between control littermates and *Cad5-BAC-Cre^{ERT2};Vhl$^{flox/flox}$* embryos.

Development • Supplementary information

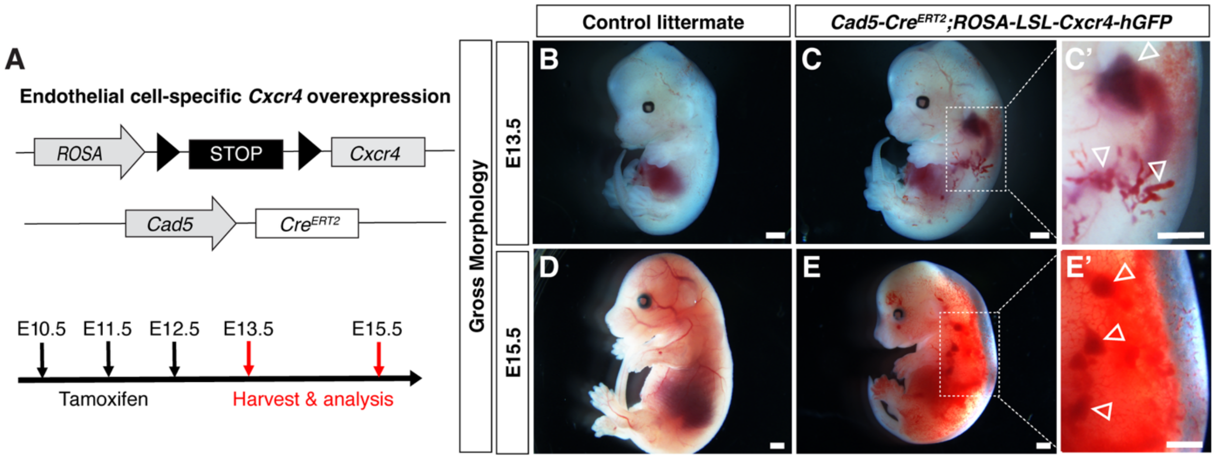

Fig. S5. Generation of EC-specific *Cxcr4* overexpression mutants

(A) Diagram illustrating the generation of the EC-specific *Cxcr4* overexpression mutants by crossing *ROSA-LSL-Cxcr4-hGFP* mice (Li et al., 2021) with tamoxifen-inducible EC specific *Cre* driver, *Cad5-BAC-Cre*^{ERT2} mice. The *Cre*-mediated excision activity was achieved by administering 3 mg tamoxifen by intraperitoneal injection (IP) at E10.5-E12.5, and embryos were harvested at E13.5 or E15.5 for analysis. (B-E) Gross morphology of control littermates (B, D) and *Cad5-BAC-Cre*^{ERT2}*;ROSA-LSL-Cxcr4-hGFP* embryos (C, E) collected from the same litter and photographed at the same magnification for each time point (E13.5 and E15.5) are shown. Hemorrhage-like phenotype (C', E', open arrowheads) was found in *Cad5-BAC-Cre*^{ERT2}*;ROSA-LSL-Cxcr4-hGFP* embryos. The dotted boxed region in C and E is magnified in C' and E', respectively. Scale bars represent 1mm.

Development • Supplementary information

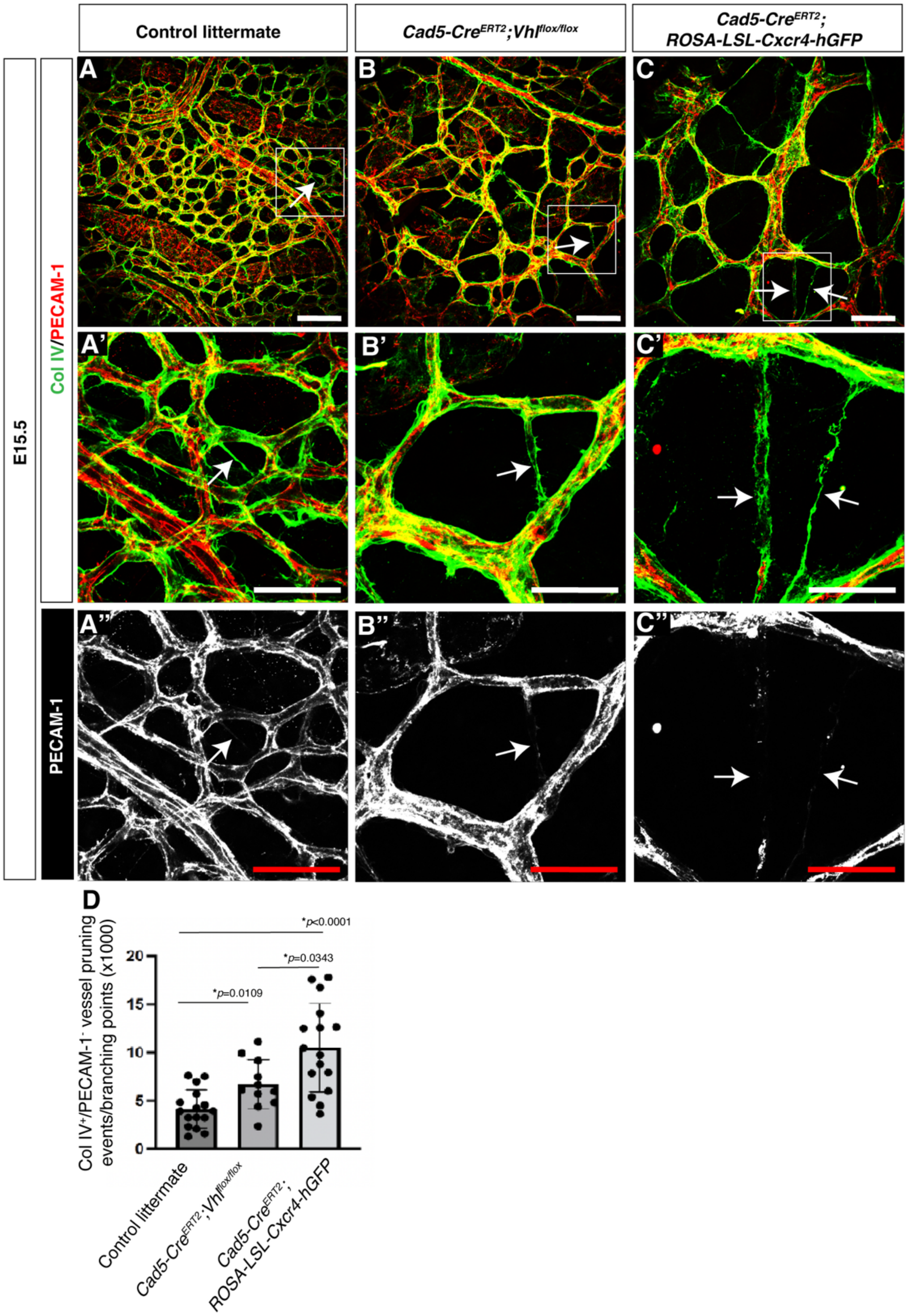

Control littermate
Cad5-CreERT2;Vhlflox/flox
Cad5-CreERT2; ROSA-LSL-Cxcr4-hGFP
E15.5
Col IV/PECAM-1
PECAM-1
A
B
C
A'
B'
C'
A''
B''
C''
D
Col IV+/PECAM-1- vessel pruning events/branching points (x1000)
*p<0.0001
*p=0.0343
*p=0.0109
20
15
10
5
0
Control littermate
Cad5-CreERT2;Vhlflox/flox
Cad5-CreERT2; ROSA-LSL-Cxcr4-hGFP

Fig. S6. Increased vascular pruning in the skin of EC-specific *Vhl* deletion and EC-specific *Cxcr4* overexpression mutants

(A-C”) Whole-mount immunohistochemical analysis with antibodies to collagen type IV (Col IV, A-C’, green), together with PECAM-1 (A-C’, red; A”-C”, white) in control littermate (A, A’, and A”), *Cad5-BAC-Cre*^{ERT2}*;Vhl*$^{flox/flox}$ (B, B’, and B”) and *Cad5-BAC-Cre*^{ERT2}*;ROSA-LSL-Cxcr4-hGFP* (C,C’ and C”) embryos at E15.5. A’-C” are the high magnification of boxed areas in A-C. Arrows indicate Col IV^{+}/PECAM-1^{-} pruning vessels. Scale bars represent 100 μm (A-C) and 50 μm (A’-C”). (D) Quantification of the number of Col IV^{+}/PECAM-1^{-} pruning events per 1000 branching points in control littermate, *Cad5-BAC-CreER*^{T2}*;Vhl*$^{flox/flox}$ and *Cad5-BAC-Cre*^{ERT2}*;ROSA-LSL-Cxcr4-hGFP* embryos at E15.5 (n=3 per genotype). Bars represent mean ± SD. Statistical *p* value was determined using the nonparametric Mann-Whitney test and a significant threshold of $p<0.05$ was considered as statistically significant differences. Asterisk indicates $p<0.05$.

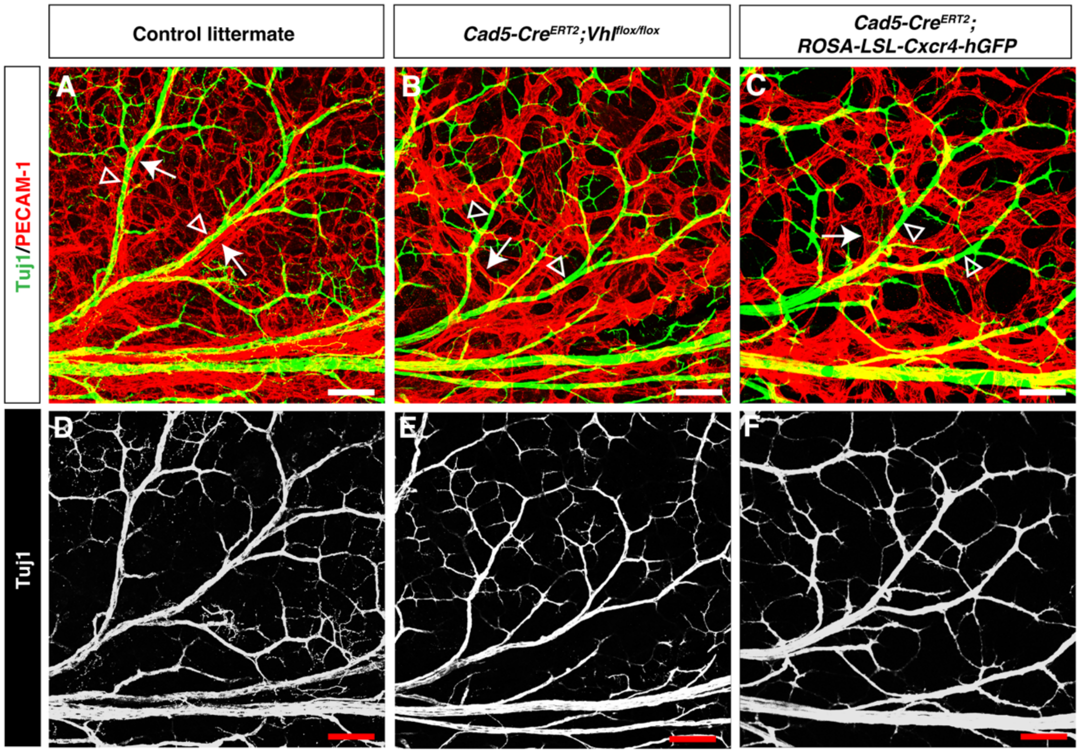

Fig. S7. Abnormal nerve-vessel alignment in the skin of EC-specific *Vhl* deletion and EC-specific *Cxcr4* overexpression mutants

(A-D) Whole-mount immunohistochemical analysis of limb skin with antibodies to neuronal βIII-tubulin (Tuj1; A-C, green; D-F, white), together with PECAM-1 (A-C, red) in control littermates (A and D), *Cad5-BAC-Cre^{ERT2};Vhl$^{flox/flox}$* (B and E) and *Cad5-BAC-Cre^{ERT2};ROSA-LSL-Cxcr4-hGFP* embryos (C and F) at E15.5. Note that remodeled blood vessels (arrows) fail to associate with nerves (open arrowheads) in the skin of *Cad5-BAC- Cre^{ERT2};Vhl$^{flox/flox}$* (B and E) and *Cad5-BAC-Cre^{ERT2};ROSA-LSL-Cxcr4-hGFP* embryos (C and F) compared to control littermates (A and D). Scale bars represent 100 µm.

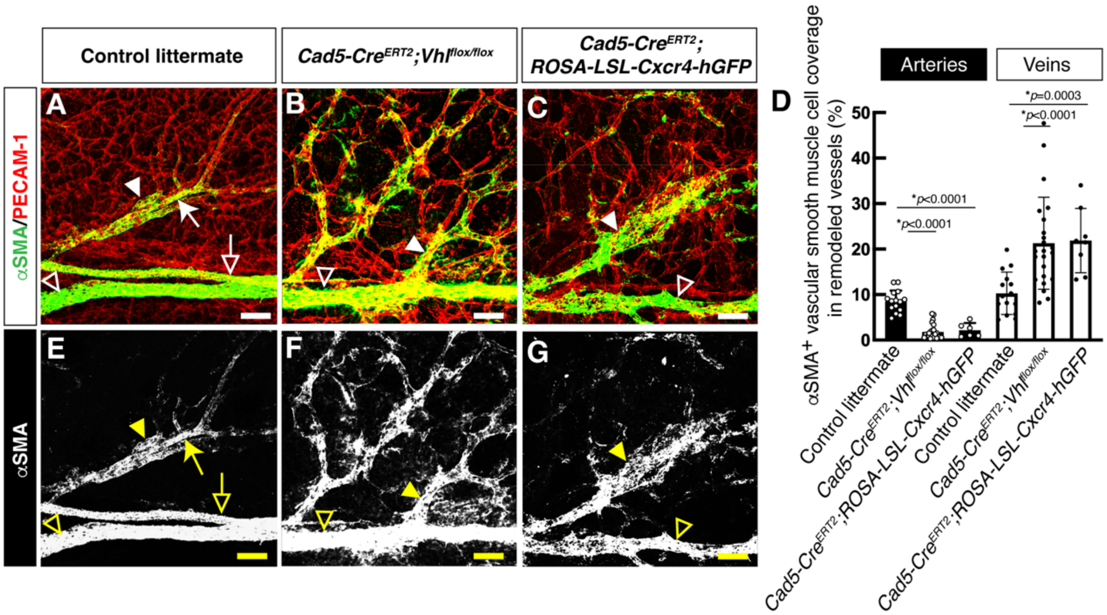

Fig. S8. Abnormal vascular smooth muscle coverage of remodeled blood vessels in the skin vasculature of EC-specific *Vhl* deletion and EC-specific *Cxcr4* overexpression mutants

(A-C and E-G) Whole-mount immunohistochemical analysis of limb skin with antibodies to the vascular smooth muscle cell (VSMC) marker αSMA (A-C, green; E-G, white), together with PECAM-1 (A-C, red) in control littermates (A and E), *Cad5-BAC-Cre^{ERT2};Vhl$^{flox/flox}$* (B and F) and *Cad5-BAC-Cre^{ERT2};ROSA-LSL-Cxcr4-hGFP* (C and G) embryos at E15.5 are shown. Arrows and open arrows indicate representative αSMA^{+} VSMC-covered arterial branches and arteries, respectively (A and E). Arrowheads and open arrowheads indicate representative αSMA^{+} VSMC-covered venous branches and veins, respectively (A-G). Scale bars represent 100 μm. (D) Quantification of αSMA^{+} VSMC coverage of remodeled blood vessels (n=15 for control littermates, n=23 for *Cad5-BAC-Cre^{ERT2};Vhl$^{flox/flox}$* embryos, n=8 for *Cad5-BAC-Cre^{ERT2};ROSA-LSL-Cxcr4-hGFP* embryos). Bars represent mean ± SEM. Statistical *p* values were determined using the nonparametric Mann-Whitney test and a significant threshold of $p<0.05$ was considered as statistically significant reduction in VSMC coverage of arteries (p<0.0001 for *Cad5-BAC-Cre^{ERT2};Vhl$^{flox/flox}$* and $p<0.0001$ for *Cad5-BAC-Cre^{ERT2};ROSA-LSL-Cxcr4-hGFP*) and increase in VSMC coverage of veins ($p<0.0001$ for *Cad5-BAC-Cre^{ERT2};Vhl$^{flox/flox}$* and $p=0.0003$ for *Cad5-BAC-Cre^{ERT2};ROSA-LSL-Cxcr4-hGFP*).

Development • Supplementary information

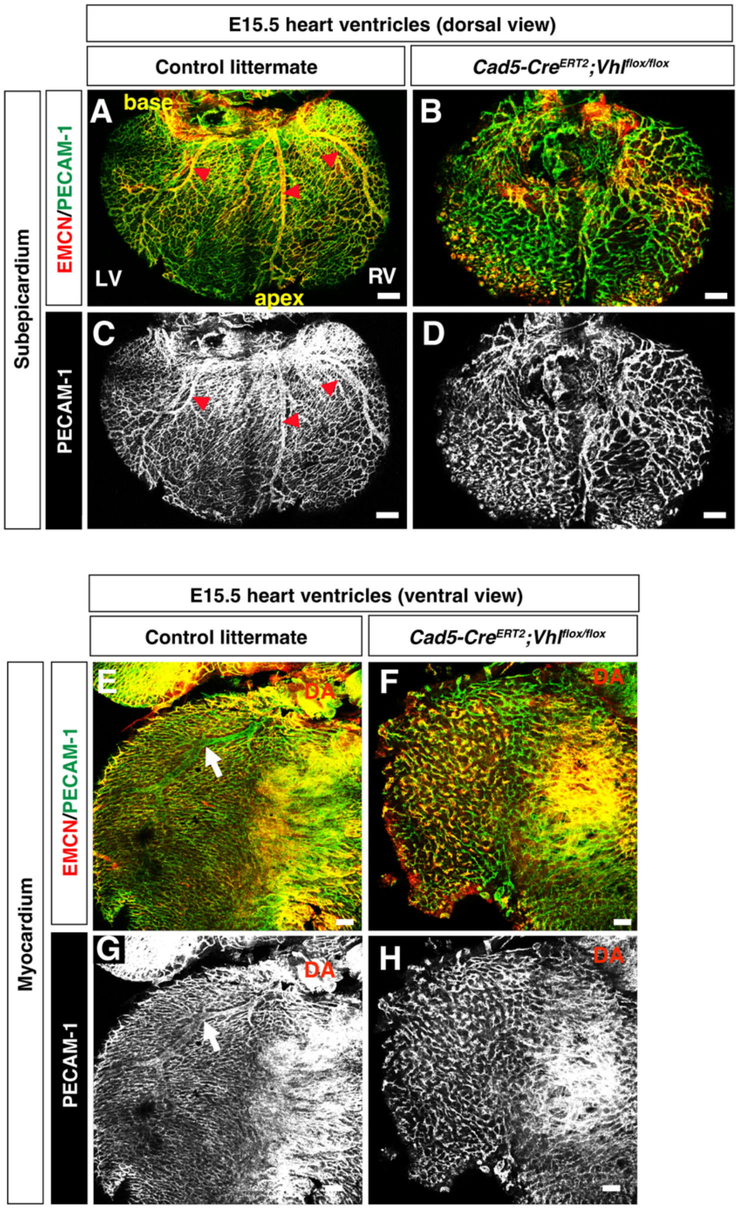

Fig. S9. Defective coronary vascular development in EC-specific *Vhl* deletion mutants

(A-D) Whole-mount immunohistochemical analysis of E15.5 hearts with antibodies to PECAM-1 (A and B, green; C and D, white) and endomucin (EMCN; A and B, red) to visualize coronary veins in the dorsal subepicardium in control littermates (A and C) and *Cad5-BAC-Cre*^{ERT2}*;Vhl*$^{flox/flox}$ embryos (B and D). EMCN^{+}/PECAM-1^{+} large-diameter venous branches were observed in the dorsal subepicardium of control littermates (A and

Development • Supplementary information

C, arrowheads) but not in *Cad5-BAC-Cre^{ERT2};Vhl$^{flox/flox}$* embryos (B and D). (E-H) Whole-mount immunohistochemical analysis of E15.5 hearts to visualize coronary arteries in the ventral myocardium of the ventricular wall in control littermates (E and G) and *Cad5-BAC-Cre^{ERT2};Vhl$^{flox/flox}$* embryos (F and H). EMCN^{-}/PECAM-1^{+} large-diameter arterial branches were observed in the ventral myocardium of the ventricular wall in control littermates (E and G, arrows) but were barely observed in *Cad5-BAC-Cre^{ERT2};Vhl$^{flox/flox}$* embryos (F and H). RV, right ventricle; LV, left ventricle; DA, dorsal aorta. Scale bars represent 100 µm.

Development • Supplementary information

Development • Supplementary information

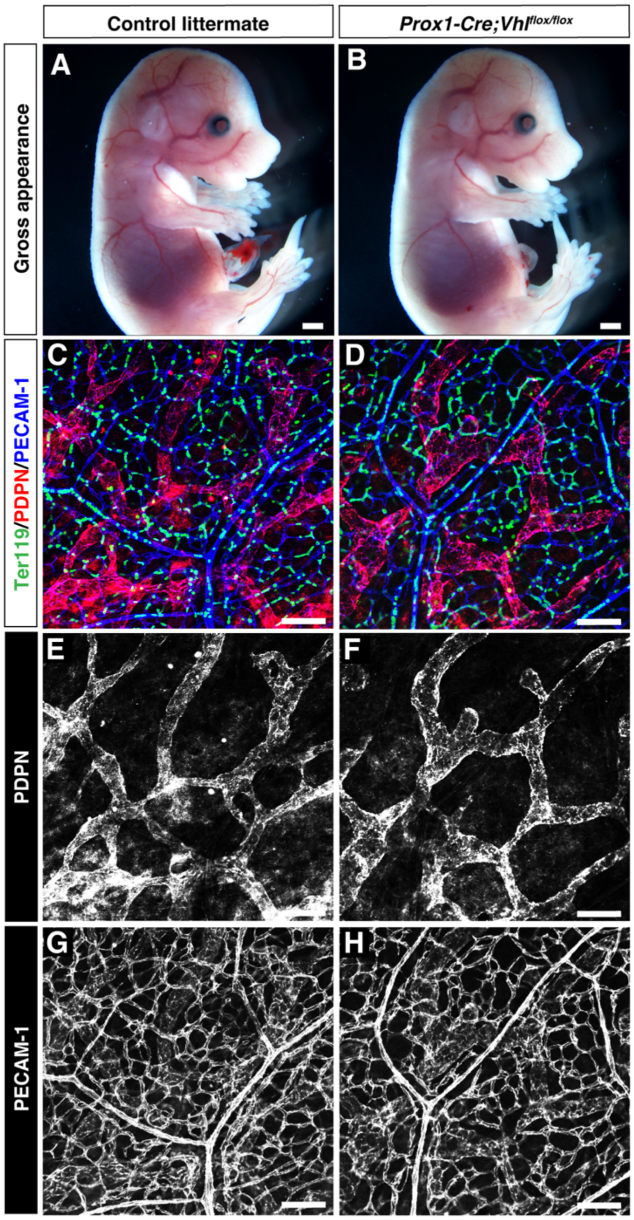

Fig. S10. Normal lymphatic vascular development in lymphatic EC-specific *Vhl* deletion mutants

(A-B) Gross appearance of control littermates (A) and *Prox1-Cre;Vhl*$^{flox/flox}$ embryos (B) at E15.5. No significant edema and blood-filled cutaneous lymphatic vessel phenotypes were observed. Scale bars represent 1mm. (C-H) Whole-mount immunohistochemical analysis of limb skin with antibodies to PDPN (C and D, red; E and F, white), Ter119 (C and D, green) and PECAM-1 (C and D, blue; G and H, white) in control littermate (C, E,

and G) and *Prox1-Cre;Vhl$^{flox/flox}$* embryos (D, F, and H) embryos at E15.5. Ter119^{+} erythrocytes were found in PECAM-1^{+}/PDPN^{-} blood vessels, but none were detected in PECAM-1^{weak}/PDPN^{+} lymphatic vessels. Additionally, no significant abnormalities were observed in the branching or size of PECAM-1^{+} blood vasculature and PDPN^{+} lymphatic vasculature in the skin of *Prox1-Cre;Vhl$^{flox/flox}$* embryos. Scale bars represent 100 μm.

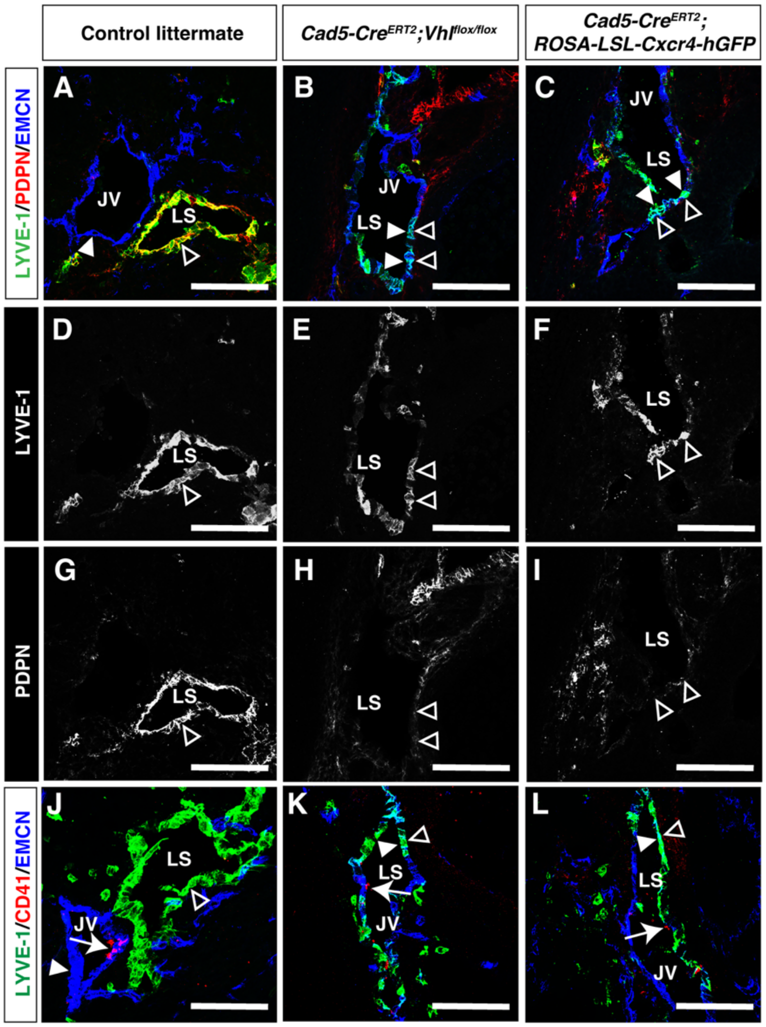

Fig. S11. Defective podoplanin expression and platelet accumulation in EC-specific *Vhl* deletion and EC-specific *Cxcr4* overexpression mutants

(A-I) Section immunohistochemical analysis of E13.5 trunk with antibodies to LYVE-1 (A-C, green; D-F, white), podoplanin (PDPN; A-C, red; G-I, white), and EMCN (A-C, blue) in control littermates (A, D and G), *Cad5-BAC-Cre^{ERT2};Vhl$^{flox/flox}$* (B, E and H) and *Cad5-BAC-Cre^{ERT2};ROSA-LSL-Cxcr4-hGFP* (C, F and I) embryos. Arrowheads indicate LYVE-1^{-}/PDPN^{-}/EMCN^{+} jugular veins, and open arrowheads indicate LYVE-1^{+}/PDPN^{+}/EMCN^{-} lymph sacs in control littermates (A, D and G). In contrast, LYVE-1^{+}/PDPN^{weak}/EMCN^{+} vessels were observed in the lymph sac of *Cad5-BAC-Cre^{ERT2};Vhl$^{flox/flox}$* (B, E and H) and *Cad5-BAC-Cre^{ERT2};ROSA-LSL-Cxcr4-hGFP* embryos (C, F and I). Additionally, in these

Development • Supplementary information

mutants, jugular veins and lymph sacs appeared to be fused together (B-C, E-F and H-I). (J-L) Section immunohistochemical analysis of E13.5 trunk with antibodies to LYVE-1 (green), the platelet marker CD41 (red), and EMCN (blue) in control littermates (J), *Cad5-BAC-Cre^{ERT2};Vhl$^{flox/flox}$* (K) and *Cad5-BAC-Cre^{ERT2};ROSA-LSL-Cxcr4-hGFP* (L) embryos. Arrows indicate CD41^{+} platelets, arrowheads indicate LYVE-1^{-}/EMCN^{+} jugular veins, and open arrowheads indicate LYVE-1^{+}/EMCN^{-} lymph sacs. Scale bars represent 100 µm. LS: lymph sac, JV: jugular vein.

Development • Supplementary information

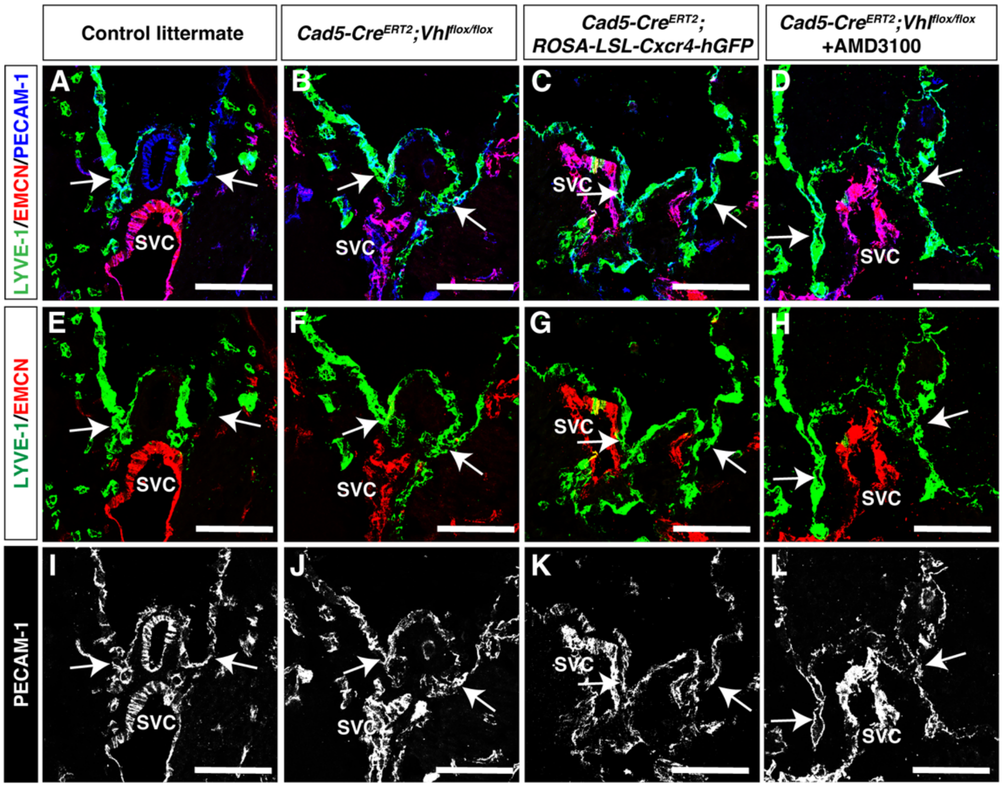

Fig. S12. No apparent differences in lymphovenous valves between control littermates, EC-specific *Vhl* deletion, EC-specific *Cxcr4* overexpression and AMD3100 treated EC-specific *Vhl* deletion mutants

(A-L) Section immunohistochemical analysis of E15.5 trunk of control littermates (A,E and I), *Cad5-BAC-Cre^{ERT2};Vhl$^{flox/flox}$* (B, F and J), *Cad5-BAC-Cre^{ERT2};ROSA-LSL-Cxcr4-hGFP* (C, G and K) and AMD3100 treated *Cad5-BAC-Cre^{ERT2};Vhl$^{flox/flox}$* (D, H and L) embryos with antibodies to LYVE-1 (green), endomucin (EMCN, red) and PECAM-1 (A-D, blue; I-L, white) are shown. Arrows indicate LYVE-1^{+}/EMCN^{-}/PECAM-1^{+} LVVs. LVVs were detected in control littermates (A,E and I), *Cad5-BAC-Cre^{ERT2};Vhl$^{flox/flox}$* (B, F and J), *Cad5-BAC-Cre^{ERT2};ROSA-LSL-Cxcr4-hGFP* (C, G and K) and *Cad5-BAC-Cre^{ERT2};Vhl$^{flox/flox}$* embryos from pregnant mice treated with AMD3100 (D, H and L). No significant changes in the structure and number of LVV were observed in these mutants, nor in EC-specific *Vhl* deletion mutants treated with AMD3100, compared to control littermates. SVC: superior vena cava. Scale bars represent 100 μm.

Development • Supplementary information

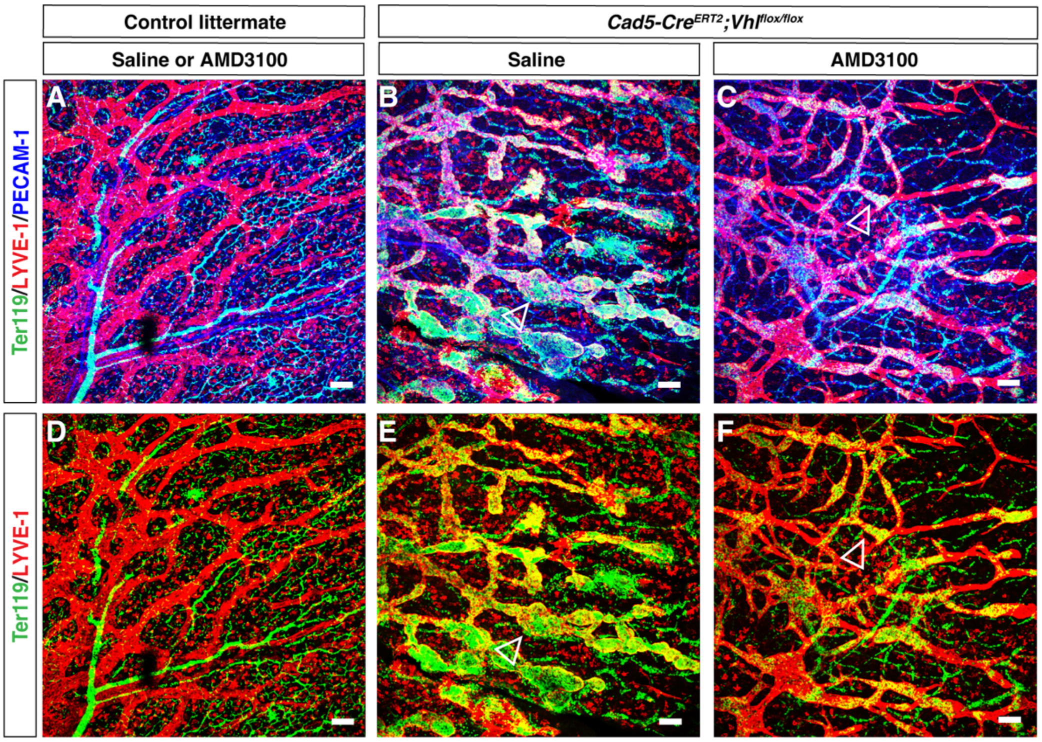

Fig. S13. The CXCR4 antagonist AMD3100 partially rescues blood-filled lymphatic vascular phenotypes in EC-specific *Vhl* deletion mutants

(A-F) Whole-mount immunohistochemical analysis of limb skin with antibodies to Ter119 and LYVE-1 (red) together with PECAM-1 (blue) in control littermate embryos from pregnant mice treated with saline or AMD3100 (A and D), *Cad5-BAC-Cre*^{ERT2};*Vhl*$^{flox/flox}$ embryos from pregnant mice treated with saline (B and E) or AMD3100 (C and F). Open arrowheads indicate representative blood filled LYVE-1^{+} (PECAM-1^{weak}) lymphatic vessels. The accumulation of Ter119^{+} erythrocytes in LYVE-1^{+} lymphatic vessels was significantly reduced in the skin of *Cad5-BAC-Cre*^{ERT2};*Vhl*$^{flox/flox}$ embryos from pregnant mice treated with AMD3100, compared to those treated with saline. Scale bars represent 100 μm.

Development • Supplementary information

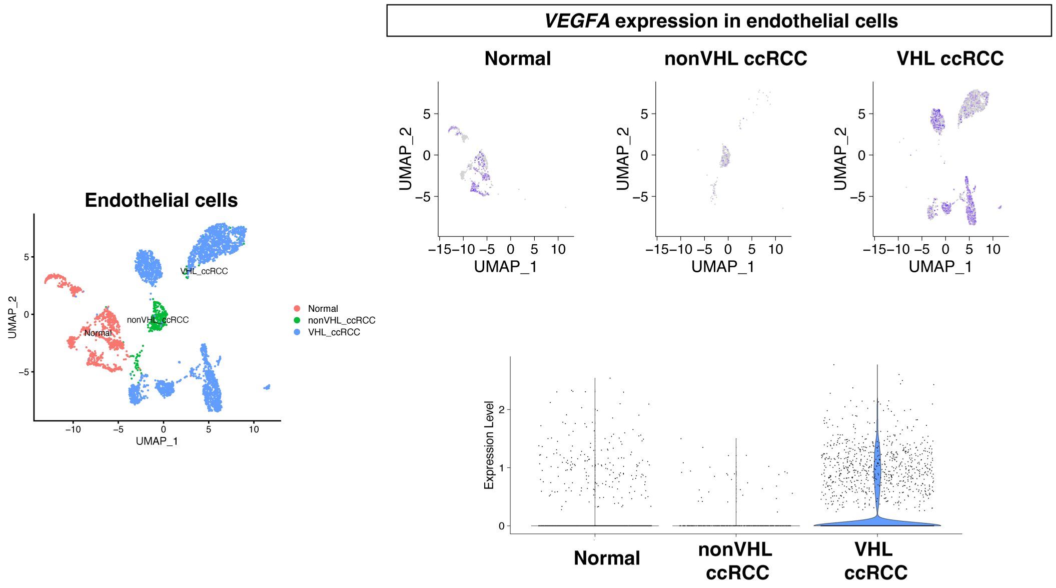

Fig. S14. Endothelial *VEGFA* expression in patients with VHL related tumors

Left: UMAP plot of scRNA-seq data representing EC clusters in individuals from normal cortex/Medulla, ccRCC tumors without *VHL* mutations, and ccRCC with *VHL* mutations. Right: UMAP plots and violin plots showing *VEGFA* expression in ECs of individuals from normal cortex/Medulla, ccRCC tumors without *VHL* mutations and ccRCC tumors with *VHL* mutations. Note that there is increased *VEGFA* expression in ECs of ccRCC with *VHL* mutations compared to ECs from other tumors or normal tissues.

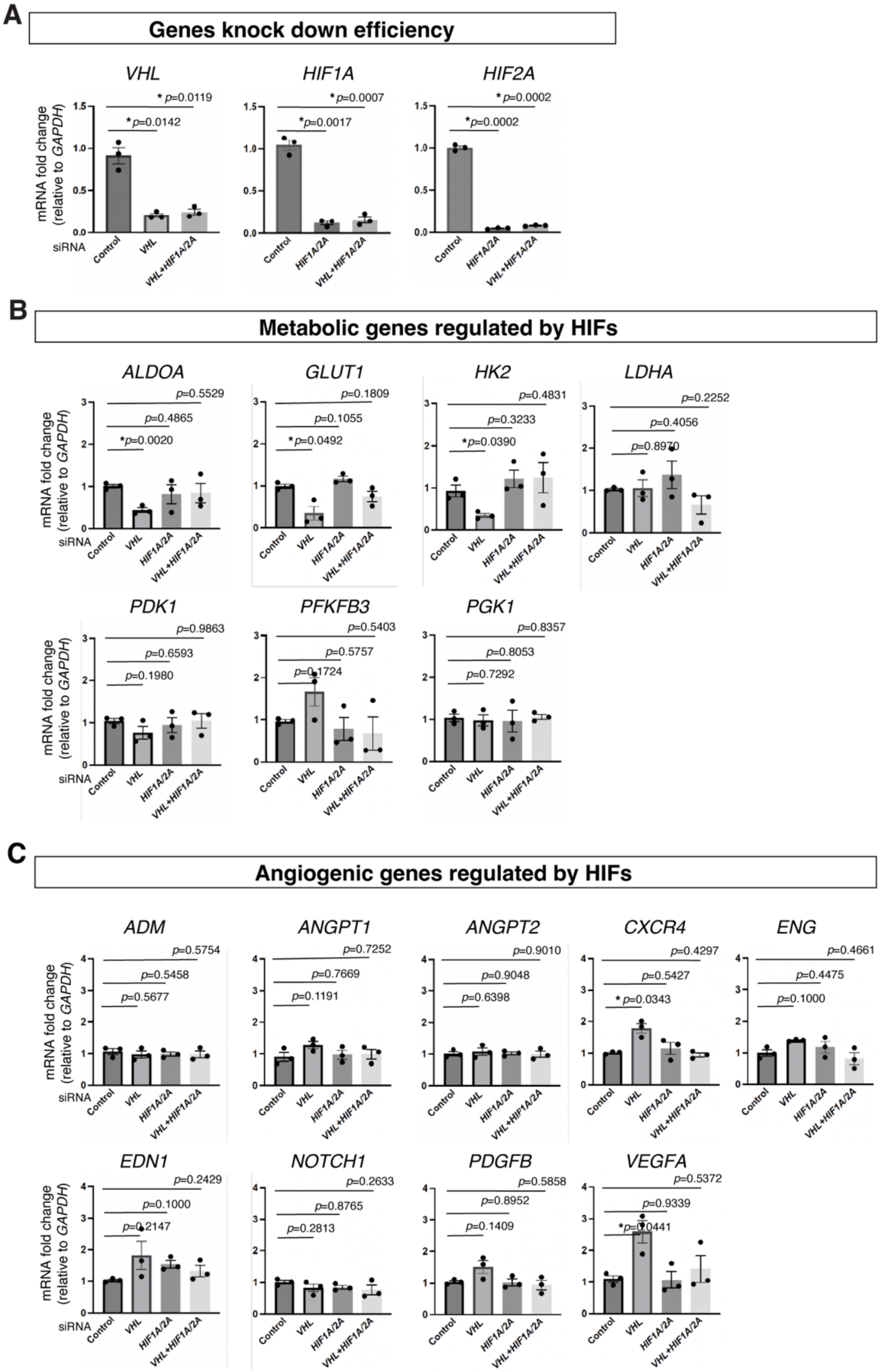

Development • Supplementary information

Fig. S15. The expression of HIF-targeted metabolic and angiogenic genes in *VHL*, *HIF1A/2A*, and *VHL*/*HIF1A/2A* knockdown HUVEC cells

(A) The fold changes in relative mRNA expression of *HIF1A*, *HIF2A*, and *VHL* in HUVECs treated with control siRNA, *VHL* siRNA, *HIF1A/HIF2A* siRNA and *VHL/HIF1A/HIF2A* siRNA was assessed by RT-qPCR (n=3). (B-C) The fold changes in relative mRNA expression of selective HIF-targeted metabolic (B) and angiogenic (C) genes in HUVECs treated with control siRNA, *VHL* siRNA, *HIF1A/HIF2A* siRNA and *VHL/HIF1A/HIF2A* siRNA was assessed by RT-qPCR (n=3). The expression of *ALDOA*, *GLUT1*, and *HK2* expression were significantly reduced in HUVECs treated with *VHL* siRNA compared to HUVECs treated with control siRNA (B). A statistically significant increase in relative mRNA expression levels with *CXCR4* and *VEGF-A* was observed in HUVECs treated with *VHL* siRNA compared to HUVECs treated with control siRNA (C). Bars represent mean ± SD. Statistical *p* values were determined using the nonparametric Mann-Whitney and a significant threshold of $p<0.05$ was considered as statistically significant differences. Asterisk indicates $p<0.05$.

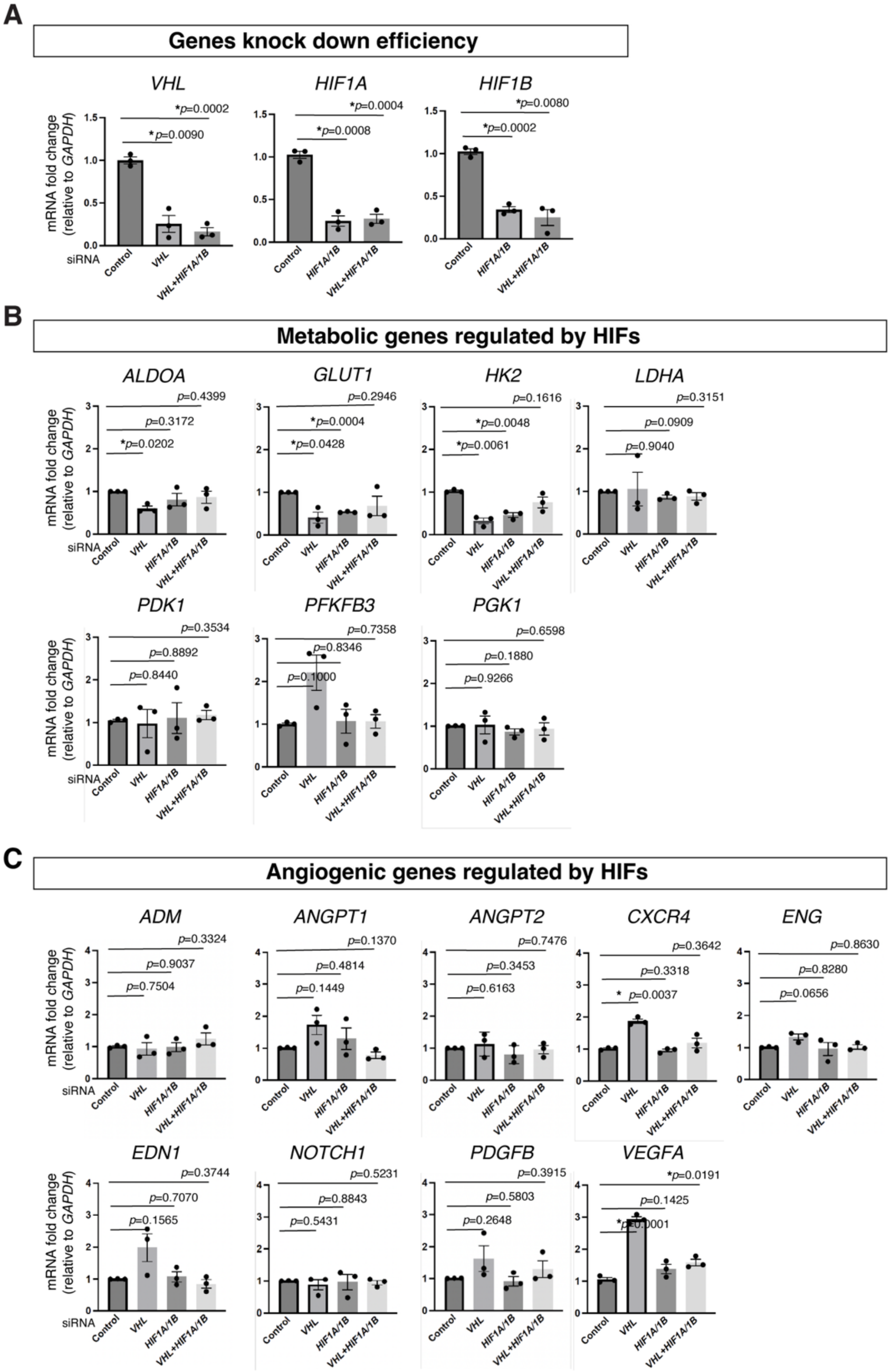
A
Genes knock down efficiency
VHL
HIF1A
HIF1B
*p=0.0002
*p=0.0090
*p=0.0004
*p=0.0008
*p=0.0080
*p=0.0002
mRNA fold change (relative to GAPDH)
siRNA
Control
VHL
HIF1A/1B
VHL+HIF1A/1B
B
Metabolic genes regulated by HIFs
ALDOA
GLUT1
HK2
LDHA
p=0.4399
p=0.3172
*p=0.0202
p=0.2946
*p=0.0004
*p=0.0428
p=0.1616
*p=0.0048
*p=0.0061
p=0.3151
p=0.0909
p=0.9040
PDK1
PFKFB3
PGK1
p=0.3534
p=0.8892
p=0.8440
p=0.7358
p=0.8346
p=0.1000
p=0.6598
p=0.1880
p=0.9266
C
Angiogenic genes regulated by HIFs
ADM
ANGPT1
ANGPT2
CXCR4
ENG
p=0.3324
p=0.9037
p=0.7504
p=0.1370
p=0.4814
p=0.1449
p=0.7476
p=0.3453
p=0.6163
p=0.3642
p=0.3318
* p=0.0037
p=0.8630
p=0.8280
p=0.0656
EDN1
NOTCH1
PDGFB
VEGFA
p=0.3744
p=0.7070
p=0.1565
p=0.5231
p=0.8843
p=0.5431
p=0.3915
p=0.5803
p=0.2648
*p=0.0191
p=0.1425
*p=0.0001

Fig. S16. The expression of HIF-targeted metabolic and angiogenic genes in *VHL*, *HIF1A/1B*, and *VHL*/*HIF1A/1B* knockdown HUVEC cells

(A) The fold changes in relative mRNA expression of *HIF1A*, *HIF1B*, and *VHL* in HUVECs treated with control siRNA, *VHL* siRNA, *HIF1A/HIF1B* siRNA and *VHL/HIF1A/HIF1B* siRNA was assessed by RT-qPCR (n=3). A statistically significant reduction in relative mRNA expression levels was observed compared to HUVECs treated with control siRNA. (B-C) The fold changes in relative mRNA expression of selective HIF-targeted metabolic (B) and angiogenic (C) genes in HUVECs treated with control siRNA, *VHL* siRNA, *HIF1A/HIF1B* siRNA and *VHL/HIF1A/HIF1B* siRNA was assessed by RT-qPCR (n=3). The expression of *ALDOA*, was significantly reduced in HUVECs treated with *VHL* siRNA, while *GLUT1*, and *HK2* expression were significantly reduced in HUVECs treated with *VHL* siRNA and *HIF1A/HIF1B* siRNA compared to HUVECs treated with control siRNA (B). A statistically significant increase in relative mRNA expression levels with *CXCR4* and *VEGF-A* was observed in HUVECs treated with *VHL* siRNA compared to HUVECs treated with control siRNA (C). Bars represent mean ± SD. Statistical *p* value was determined using the nonparametric Mann-Whitney test and a significant threshold of $p<0.05$ was considered as statistically significant differences. Asterisk indicates $p<0.05$.

Table S1. Primer sequences used for qRT-PCR

Knockdown and knockout genes

Gene Symbol	Forward (5'→3')	Reverse (5'→3')	GenBank accession number
human *VHL*	GCAGGCGTCGAAGAGTACG	CGGACTGCGATTGCAGAAGA	NM_198156
human *HIF1A*	CACCACAGGACAGTACAGGAT	CGTGCTGAATAATACCACTCACA	NM_001243084
human *HIF2A*	CGGAGGTGTTCTATGAGCTGG	AGCTTGTGTGTTCGCAGGAA	NM_001430
human *HIF1B*	CTGCCAACCCCGAAATGACAT	CGCCGCTTAATAGCCCTCTG	NM_001197325
human *ACTB*	CATGTACGTTGCTATCCAGGC	CTCCTTAATGTCACGCACGAT	NM_001101
human *GAPDH*	GTCTCCTCTGACTTCAACAGCG	ACCACCCTGTTGCTGTAGCCAA	NM_002046
mouse *Vhl*	TGTGCCATCCCTCAATGTCG	GCACCGCTCTTTCAGGGTA	NM_009507
mouse *Actb*	GGCTGTATTCCCCTCCATCG	CCAGTTGGTAACAATGCCATGT	NM_007393

Angiogenic genes regulated by HIFs

Gene Symbol	Forward (5'→3')	Reverse (5'→3')	GenBank accession number
mouse *Adm*	CACCCTGATGTTATTGGGTTCA	TTAGCGCCCACTTATTCCACT	NM_009627
mouse *Angpt1*	CACATAGGGTGCAGCAACCA	CGTCGTGTTCTGGAAGAATGA	NM_009640
mouse *Angpt2*	CCTCGACTACGACGACTCAGT	TCTGCACCACATTCTGTTGGA	NM_007426
mouse *Cxcr4*	GACTGGCATAGTCGGCAATG	AGAAGGGGAGTGTGATGACAA A	NM_001420067
mouse *Eng*	CCCTCTGCCCATTACCCTG	GTAAACGTCACCTCACCCCTT	NM_007932
mouse *Edn1*	GCACCGGAGCTGAGAATGG	GTGGCAGAAGTAGACACACTC	NM_010104
mouse *Notch1*	GATGGCCTCAATGGGTACAAG	TCGTTGTTGTTGATGTCACAGT	NM_008714
mouse *Pdgfb*	CATCCGCTCCTTTGATGATCTT	GTGCTCGGGTCATGTTCAAGT	NM_011057
mouse *Vegfa*	AAGGAGAGCAGAAGTCCCATGAAGT	TTCACATCTGCTGTGCTGTAGGAAG	NM_009505.4
human *ADM*	ATGAAGCTGGTTTCCGTCG	GACATCCGCAGTTCCCTCTT	NM_001124
human *ANGPT1*	AGCGCCGAAGTCCAGAAAAC	TACTCTCACGACAGTTGCCAT	NM_001146
human *ANGPT2*	AACTTTCGGAAGAGCATGGAC	CGAGTCATCGTATTCGAGCGG	NM_001147
human *CXCR4*	ACTACACCGAGGAAATGGGCT	CCCACAATGCCAGTTAAGAAGA	NM_003467
human *ENG*	TGCACTTGGCCTACAATTCCA	AGCTGCCCACTCAAGGATCT	NM_001114753
human *EDN1*	AGAGTGTGTCTACTTCTGCCA	CTTCCAAGTCCATACGGAACAA	NM_001168319
human *NOTCH1*	GAGGCGTGGCAGACTATGC	CTTGTACTCCGTCAGCGTGA	NM_017617
human *PDGFB*	CTCGATCCGCTCCTTTGATGA	CGTTGGTGCGGTCTATGAG	NM_033016
human *VEGFA*	AGAAGGAGGAGGGCAGAATCA	TCAGGGTACTCCTGGAAGATGTC	NM_001171623

Development • Supplementary information

Metabolic genes regulated by HIFs

Gene Symbol	Forward (5'→3')	Reverse (5'→3')	GenBank accession number
mouse *Aldoa*	CGTGTGAATCCCTGCATTGG	CAGCCCCTGGGTAGTTGTC	NM_001177308
mouse *Glut1(Slc2a1)*	CAGTTCGGCTATAACACTGGTG	GCCCCCGACAGAGAAGATG	NM_011400
mouse *Hk2*	AACCGCCTAGAAATCTCCAGA	TGATCGCCTGCTTATTCACGG	NM_013820
mouse *Ldha*	TGTCTCCAGCAAAGACTACTGT	GACTGTACTTGACAATGTTGGGA	NM_001136069
mouse *Pdk1*	GGACTTCGGGTCAGTGAATGC	TCCTGAGAAGATTGTCGGGGA	NM_172665
mouse *Pfkfb3*	GGGGAGTTGGTCAGCTTCG	CCCAGAGCCGGGTACAGAA	NM_001171227
mouse *Pgk1*	ATGTCGCTTTCCAACAAGCTG	GCTCCATTGTCCAAGCAGAAT	NM_008828
human *ALDOA*	CAGGGACAAATGGCGAGACTA	GGGGTGTGTTCCCCAATCTT	NM_000034
human *GLUT1(SLC2A1)*	ATTGGCTCCGGTATCGTCAAC	GCTCAGATAGGACATCCAGGGTA	NM_006516
human *HK2*	TGCCACCAGACTAAACTAGACG	CCCGTGCCCACAATGAGAC	NM_000189
human *LDHA*	TTGACCTACGTGGCTTGGAAG	GGTAACGGAATCGGGCTGAAT	NM_001165415
human *PDK1*	CTGTGATACGGATCAGAAACCG	TCCACCAAACAATAAAGAGTGCT	NM_002610
human *PFKFB3*	TTGGCGTCCCCACAAAAGT	AGTTGTAGGAGCTGTACTGCTT	NM_004566
human *PGK1*	GACCTAATGTCCAAAGCTGAGAA	CAGCAGGTATGCCAGAAGCC	NM_000291

Table S2. Secondary antibodies used for immunostaining

Antibodies	Source	Catalogue codes
Goat anti-Rat IgG (H+L) Cross-adsorbed, Alexa Fluor 488	Thermo Fisher Scientific	A11006
Goat anti-Rat IgG (H+L) Cross-adsorbed, Alexa Fluor 568	Thermo Fisher Scientific	A11077
Goat anti-Rabbit IgG (H+L) Highly Cross-Adsorbed, Alexa Fluor 488	Thermo Fisher Scientific	A11034
Goat anti-Rabbit IgG (H+L) Cross-Adsorbed, Alexa Fluor 568	Thermo Fisher Scientific	A11011
Goat anti-Mouse IgG2a Cross-Adsorbed, Alexa Fluor 488	Thermo Fisher Scientific	A21131
Goat anti-Mouse IgG2a Cross-Adsorbed, Alexa Fluor 568	Thermo Fisher Scientific	A21134
Goat anti-Mouse IgG2a Cross-Adsorbed, Alexa Fluor 633	Thermo Fisher Scientific	A21136
Goat anti-Armenian Hamster IgG (H+L), Alexa Fluor 488 AffiniPure	Jackson ImmunoResearch Labs	127-545-160
Goat anti-Armenian Hamster IgG (H+L), Cy3 AffiniPure	Jackson ImmunoResearch Labs	127-165-160
Goat anti-Armenian Hamster IgG (H+L), Alexa Fluor 647 AffiniPure	Jackson ImmunoResearch Labs	127-605-160
Goat anti-Syrian Hamster IgG (H+L), Alexa Fluor 647 AffiniPure	Jackson ImmunoResearch Labs	107-605-142
Goat anti-Rat IgG (H+L), Alexa Fluor 647 AffiniPure	Jackson ImmunoResearch Labs	112-605-003
Goat anti-Rabbit IgG (H+L), Alexa Fluor 647 AffiniPure	Jackson ImmunoResearch Labs	111-605-144
Fab Fragment Donkey anti-Goat IgG (H+L), Alexa Fluor 488 AffiniPure	Jackson ImmunoResearch Labs	705-547-003
