# Supplementary material for: Endothelial von Hippel-Lindau gene deletion causes abnormal blood and lymphatic vasculature through ectopic activation of the HIF-CXCR4 axis
Source: Development. 2026 May 18;153(10):dev204519. doi: 10.1242/dev.204519 (PMC13282565; doi:10.1242/dev.204519)
Supplement: Supplementary file 1 [file develop-153-204519_review_history.pdf]

Development

# Endothelial von Hippel-Lindau deletion causes abnormal blood and lymphatic vasculature through ectopic activation of the HIF-CXCR4 axis

Wenling Li, Koh Nakayama, Ryo Sato, Rina Shimada, Yoshiaki Kubota and Yoh-suke Mukouyama
DOI: 10.1242/dev.204519

**Editor**: Haruhiko Koseki

**Review timeline**

| | |
|---|---|
| Original submission: | 11 November 2024 |
| Editorial decision: | 15 December 2024 |
| First revision received: | 8 August 2025 |
| Editorial decision: | 7 September 2025 |
| Second revision received: | 17 December 2025 |
| Accepted: | 24 December 2025 |


---

**Original submission**

First decision letter

MS ID#: dev.204519

MS Title: Endothelial Von Hippel-Lindau Deletion Causes Abnormal Vascular Network Formation Through Ectopic Activation of Hif-Cxcr4 Axis

Authors: Wenling Li; Koh Nakayama; Ryo Sato; Rina Shimada; Yoshiaki Kubota; Yoh-suke Mukouyama
Article Type: Research Article

Dear Dr Mukouyama,

I have now received all the referees' reports on the above manuscript, and have reached a decision. The referees' comments are appended below, or you can access them online: please go to:

As you will see, the referees express considerable interest in your work, but have some significant criticisms and recommend a substantial revision of your manuscript before we can consider publication. If you are able to revise the manuscript along the lines suggested, which may involve further experiments, I will be happy receive a revised version of the manuscript. Your revised paper will be re-reviewed by one or more of the original referees, and acceptance of your manuscript will depend on your addressing satisfactorily the reviewers' major concerns. Please also note that Development will normally permit only one round of major revision. If it would be helpful, you are welcome to contact us to discuss your revision in greater detail. Please send us a point-by-point response indicating your plans for addressing the referees' comments, and we will look over this and provide further guidance.

Please attend to all of the reviewers' comments and ensure that you clearly highlight all changes made in the revised manuscript. Please avoid using 'Tracked changes' in Word files as these are lost in PDF conversion. I should be grateful if you would also provide a point-by-point response detailing how you have dealt with the points raised by the reviewers in the 'Response to Reviewers' box. If you do not agree with any of their criticisms or suggestions please explain clearly why this is so.

© 2026. Published by The Company of Biologists under the terms of the Creative Commons Attribution License (https://creativecommons.org/licenses/by/4.0/).

Reviewer 1

SUMMARY OF THE ADVANCE MADE IN THIS PAPER AND ITS POTENTIAL SIGNIFICANCE TO THE FIELD

The manuscript of Li et al. describes the effects of VHL deletion in endothelial cells during skin vascularization in mouse embryos. Using genetic models, the authors show this mutation leads to the activation of the HIF-CXCR4 axis that induces arterial-venous malformations and disturbance of vessel patterning in the skin. Targeting CXCR4 overexpression with AMD3100 partially restores the arterial-venous patterning. Analyzing publicly available single-cell RNA-sequencing data of endothelial cells from patients with VHL syndrome, Li et al. found that the vascular abnormalities observed in the disease are linked to an ectopic activation of the HIF-CXCR4 axis.

This article describes a very elegant work that pursues previous findings published by the Mukouyama's group. Find below some points of discussion that would permit me to clarify some misunderstood results.

SUGGESTIONS TO AUTHORS

Mukouyama's group previously shows that nerves guide vessels during the maturation of the vascular tree. Is the distribution of nerves in skin of mutants normal or anarchic, and why are the nerves not able to partly guide mutant vessels?

Watching to embryos in Fig 1B-E', the vascular plexus is more abundant in mutants than in WTs: does it mean that pruning is also inhibited?

Concerning the expression of CXCR4 (Fig 1F-I), the authors wrote (line 143) that a larger number of endothelial cells express the receptor: watching to Fig 1H and I, this observation is due to an increase of endothelial cell population between WTs and mutants, but the expression of CXCR4 per se is not increased.

VHL inhibition stabilizes HIF that stimulates VEGF A. Nevertheless, the VEGF protein is unchanged between WT and mutants (FigS1 B-E): the authors discuss about a post-translational regulation that would restore VEGF A protein levels and autocrine signaling in the mutants (lines 366-367). However, authors also precise (line 365) that VEGF A is important for maintaining the vascular integrity. Thus, the recovery of VEGF A protein levels would have to maintain a vessel integrity in mutants. The point has to be clarified for me. Furthermore, concerning patients with VHL syndrome that induces VEGF mRNA overexpression, what is known about the variation of VEGF at the protein level?

Line 96: I do not understand what means "ectopic CXCR4 expression"?

Concerning the expression of HIF-targeted angiogenic genes in ECs from EC-specific VHL deletion mutants (FigS1 A), I note that Notch expression is increased in deleted mice, while the difference is not significant ($p=0.635$). Maybe that one or two more cases would permit to reach $p<0.05$ and it would be interesting to study Notch as it is known to play a role during vessel identity and patterning.

Watching to Fig 1L, only more or less 10% of endothelial cells express HIF in their nuclei: thus, how can this weak percentage explain the vascular abnormalities observed in the mutants?

Concerning in vitro experiments, why do the authors use HUVECs rather than HUAECs or both cell lines? Furthermore, in Fig 2C, I do not understand why VHL siRNAs do not induce an increase of HIF 1A and HIF 2A expression?

Fig 3H, how do the authors explain the (significant?) decrease in branch points at E15.5 between VHL mutants and CXCR4 overexpression mice?

In the discussion, the authors wrote (line 357) that "in the absence of arterial branches, venous branching is impaired". However, I observed in Fig 6H and I that, while arterial branching is

© 2026. Published by The Company of Biologists under the terms of the Creative Commons Attribution License (https://creativecommons.org/licenses/by/4.0/). 

partially restored after AMD3100 treatment, it does not induce the venous alignment along arteries. Is there any explanation about this observation?

Minor points

Line 62 add "the" between "is" and "vascular"
Line 238 correct "(FigS3B)"
Line 476 M&M How many-time lasts the fixation?

Legends

Figure 1J and K The lumen of vessels has to be indicated to clarify what the reader watches.

Reviewer 2

SUMMARY OF THE ADVANCE MADE IN THIS PAPER AND ITS POTENTIAL SIGNIFICANCE TO THE FIELD

The current study by Li et. al. investigates the endothelial-specific role of VHL during developmental angiogenesis and lymphangiogenesis. The primary significance of this study is that it addresses the role of VHL/HIF in vascular endothelial cells. Whereas most prior studies ascribed important roles to VHL in regulating HIF during normoxia / hypoxia and tumor angiogenesis in many cell types, its specific functions in endothelial cells remained less understood. Employing a conditional floxed Vhl allele, the authors elegantly demonstrate an essential role for VHL in atrial and venous morphogenesis and differentiation, as well as lymphangiogenesis. The authors expand upon their prior study (Li et. al., 2021), where a gain-of-function model for CXCR4 over-expression (OE) in endothelial cells phenocopies the Vhl LOF model presented here. This study clarifies a regulatory and functional connection between VHL-HIF1 and CXCR4 signaling, providing evidence that VHL regulation of HIF target gene expression is endothelial cell-autonomously required for normal vascular development. The characterization of arterial, venous, and lymphatic vessel phenotypes is well interrogated, and the data is clearly presented and convincing. The immunofluorescent images are noteworthy and of exceptional quality.

I have several comments and questions that if addressed would further enhance the impact of this study.

SUGGESTIONS TO AUTHORS

1. The authors employed a floxed Vhl allele inactivated by a Cad5-driven CreERT2. Was the extent of VHL depletion in endothelial cells examined? It would be informative to know if the Vhl gene was ablated completely or partially.
2. In figure S1 the authors show that of the HIF1α target genes assessed, Vegfa was the most transcriptionally upregulated in Vhl cKO embryos (~3 fold). Surprisingly, however, endothelial levels of VEGFA protein are apparently unaltered. I find this to be rather unexpected and difficult to interpret based on the immunohistochemical methods used. The comparison to CXCR4, which shows a clear increase on endothelial cells despite a modest mRNA upregulation (~1.5 fold), cannot be made as it is a plasma membrane protein whereas VEGFA is secreted. A more appropriate method would be to perform an ELISA on tissue homogenates or on conditioned media from isolated WT versus VHL-null endothelial cells.
3. The authors chose to narrow their investigation on a relatively small subset of HIF1α target genes, namely relevant to angiogenesis (e.g. Ang1, Ang2). Were other well-recognized HIF1α targets interrogated? For example, regulators of glycolysis (e.g. Pgk1, Pdk1, Hk2) would inform on metabolic alteration in ECs. Also, changes in adrenomedulin (Adm), a specific HIF1α target and a regulator of lymphangiogenesis (Fritz-Six, JCI, 2008), may be relevant to the lymphatic defects and edema exhibited by VHL mutants. Perhaps molecular profiling (RNAseq) could provide a broader perspective on pathway perturbations resulting from endothelial VHL loss of function.
4. In figure 6, the authors performed studies to nicely demonstrate the rescue of VHL LOF phenotypes by CXCR4 antagonism. Have they considered performing similar experiments in the CXCR4 OE model?

© 2026. Published by The Company of Biologists under the terms of the Creative Commons Attribution License (https://creativecommons.org/licenses/by/4.0/).

5. The authors focused on dermal vessel phenotypes, but I wonder if they observed defects in specific organs as well (heart, lung, kidney)?
6. Suggestion for future study: the alleles characterized here perfectly lend themselves for performing studies in the retinal angiogenesis model. This well-established model offers several advantages including well-defined and manipulable developmental stages, stereotypical venous and arterial specification, and clearly delineated angiogenic domains, all amenable for quantification. I wonder if the authors have performed confirmatory experiments in this model or are planning to in future studies.

Reviewer 3

SUMMARY OF THE ADVANCE MADE IN THIS PAPER AND ITS POTENTIAL SIGNIFICANCE TO THE FIELD

In this manuscript Li et al report that the deletion of VHL from endothelial cells (ECs) results in the overexpression of HIF1A and its target molecule CXCR4. A loss of arterial identity, reduced vascular branching and blood-filled lymphatics were observed in mice lacking VHL in ECs (VHL-ECKO). They previously showed that the overexpression (OE) of CXCR4 in blood ECs, but not lymphatic endothelial cells (LECs) result in a similar phenotype. Therefore, they hypothesized that the CXCR4 OE in ECs is the primary cause of the vascular phenotype in VHL-ECKO mice. They tested this hypothesis by treating control and VHL-ECKO embryos with the CXCR4 antagonist AMD3100 and demonstrate a partial rescue of the phenotypes.
Overall, the experiments are well done, data clear to appreciate and manuscript is well-written. Although the phenotypes of VHL-ECKO and CXCR4-OE models are not new the mechanistic connection between the two seems to be novel. Nevertheless, I do have a few additional experiments to make the manuscript stronger.

SUGGESTIONS TO AUTHORS

Major suggestions

1. Blood-lymph separation is regulated by the glycoprotein podoplanin in LECs and C-type lectin CLEC2 in platelets. Check whether the expression of podoplanin is normal in LECs. Determine whether platelets are normally present in the VHL-ECKO and CXCR4 OE embryos.
2. The authors are suggesting a connection between lymph sacs and jugular vein in VHL KO and CXCR4 OE embryos. These images are very convincing. Better images can be provided. Additionally, blood and lymphatic vasculatures interact through lymphovenous valves. Whether these valves are normal in VHL-ECKO and CXCR4 OE embryos must be determined. Whether these defects are ameliorated by AMD3100 must be tested.
3. The authors previously showed that the OE of CXCR4 in LECs does not result in blood-filled lymphatics. They must test whether deletion of VHL from LECs results in any phenotype.

Minor suggestions

1. Bulk RNA-seq experiments are reasonably inexpensive to do. It will be interesting to generate this data for siVHL, siHIF1A+siHIF1B and siVHL+siHIF1A+siHIF1B samples.

---

**First revision**

Author response to reviewers' comments

We would like to thank the reviewers for their positive and insightful comments and suggestions, which led to a great improvement in our study and manuscript. We addressed the reviewers' suggestions as follows.

© 2026. Published by The Company of Biologists under the terms of the Creative Commons Attribution License (https://creativecommons.org/licenses/by/4.0/). 

**REVIEWER 1**

***Comments:*** *…..This article describes a very elegant work that pursues previous findings published by the Mukouyama's group. Find below some points of discussion that would permit me to clarify some misunderstood results.*

**Response**: We greatly appreciate the Reviewer's encouraging comments. We believe that the additional data and information we present in response to the Reviewer's comments clarifies our results and conclusions.

***Major suggestion****: Mukouyama's group previously shows that nerves guide vessels during the maturation of the vascular tree. Is the distribution of nerves in skin of mutants normal or anarchic, and why are the nerves not able to partly guide mutant vessels?*

**Response:** We thank the Reviewer's insightful questions.

- To address this issue, we performed whole-mount immunohistochemical staining on embryonic limb skin from endothelial cell (EC)-specific *VHL* deletion mutants and EC-specific *Cxcr4* overexpression mutants, as well as their control littermates, using antibodies to neuronal ßIII-tubulin (Tuj1) as a pan-axonal marker together with the pan-EC marker PECAM-1. Our findings show that there are no apparent changes in the pattern of Tuj1+ nerve branching in EC-specific *VHL* deletion mutants and EC-specific *Cxcr4* overexpression mutants when compared to control littermates at E15.5.
  - We observed that small- and mid-diameter remodeled blood vessels were associated with nerves in the control littermates. However, in both EC-specific *VHL* deletion mutants and EC-specific *Cxcr4* overexpression mutants, the nerve-vessel alignment was severely disrupted. We have now included these new data in **Figure S6** and have mentioned the corresponding findings in the RESULTS section in the revised manuscript (lines 225-233).
  - These findings are consistent with our previous data published (Li et al. *Dev Biol* 2021 PMID:34015362) in which we demonstrated that EC-specific *Cxcr4* overexpression leads to an abnormal vascular branching network and perturbs nerve-vessel alignment in the embryonic skin vasculature.
- The question of why the nerves are unable to partially guide mutant blood vessels is an important one. Our previous findings (Li et al. *Dev Cell* 2013 PMID:23395391, Li et al. *Dev Biol* 2021 PMID:34015362) suggest that, before establishing nerve-vessel alignment at E15.5, a specific subset of ECs expressing CXCR4 in the capillary network at E13.5 appears to be pre-specified as arterial ECs. Nerve-derived CXCL12 recruits these CXCR4+ ECs, while nerve-derived VEGF-A promotes arterial differentiation. Since both EC-specific *VHL* deletion mutants and EC-specific *Cxcr4* overexpression mutants show CXCR4 expression in most capillary ECs, this ectopic expression of CXCR4 may disrupt the selective recruitment of arterial ECs, resulting in impaired nerve-vessel alignment and arterial morphogenesis. We have discussed this issue in the DISCUSSION section in the revised manuscript (lines 457-470).

***Major suggestion****: Watching to embryos in Fig 1B-E', the vascular plexus is more abundant in mutants than in WTs: does it mean that pruning is also inhibited?*

**Response:** We thank the Reviewer's insightful comments regarding the abundance of vascular plexus in the mutants.

- We acknowledge that establishing clear differences in vascular morphology based solely on the gross images presented in Figures 1B-E' in the original version of the manuscript can be challenging. However, our findings suggest that the observed difference in vascular plexus between the mutants and their control littermates may reflect a hemorrhage-like vascular phenotype, characterized by the presence of blood-filled lymphatic vasculature in the mutants. Supporting this observation, our whole-mount immunohistochemical staining showed blood-filled LYVE-1+ lymphatic vessels containing Ter119+ erythrocytes in the skin of EC-specific *VHL* deletion mutants, as presented in **Figure 5** in the revised manuscript.

© 2026. Published by The Company of Biologists under the terms of the Creative Commons Attribution License (https://creativecommons.org/licenses/by/4.0/).

- To further investigate the vascular plexus, we conducted whole-mount immunohistochemical staining using the pan-EC marker PECAM-1, as presented in **Figure 3** in the revised manuscript. This analysis revealed two significant phenotypes in the vascular plexus of the mutants. Nonetheless, these changes do not indicate a more abundance of vascular plexus in the mutants compared to their control littermates.
    - At E13.5, the control littermates exhibited a well-defined, honeycomb-shaped capillary network, whereas EC-specific *VHL* deletion mutants exhibited enlarged capillaries with reduced branching complexity. This observation suggests that vascular plexus may be less abundant in the mutants compared to their control littermates.
    - By E15.5, the capillary network in control littermates had remodeled into a branched, hierarchical structure, a critical aspect of proper vascular development. However, EC-specific *VHL* deletion mutants showed a disorganized network with enlarged capillaries and reduced branching complexity. This observation also suggests that vascular plexus may be less abundant in the mutants compared to their control littermates.
    - We should note that similar vascular abnormalities were also observed in EC-specific *Cxcr4* overexpression mutants.
- Vessel pruning, the regression of selected vascular branches, significantly influences vascular plexus expansion in the developing mouse skin vasculature (Kam et al. *Cell* 2023 PMID:37167971) and the developing mouse retinal vasculature (Franco et al. *PLoS Biol* 2015 PMID:25884288). Following previously reported regression profiles (Franco et al. *PLoS Biol* 2015 PMID:25884288), which identify vessel segments that are positive for the EC matrix collagen IV and negative for the EC marker PECAM-1, we conducted whole-mount immunohistochemical staining using collagen IV and PECAM-1, as presented in **Figure S5** in the revised manuscript. Our results indicate a significant increase in the number of regression events per branching point in EC-specific *VHL* deletion mutants compared to the control littermates. These findings, combined with the aforementioned analysis of vascular branching complexity, suggest that both mutants enhance vessel pruning, while the mutants have enlarged capillaries with reduced branching complexity. We have now included these new data in **Figure S5** and have mentioned the corresponding findings in the RESULTS section in the revised manuscript (lines 209-223).
    - Conducting longitudinal imaging of skin vasculature in live embryos over two days, despite its current challenges, could provide valuable insights into the changes in vessel pruning between EC-specific *VHL* deletion mutants and the control littermates.

***Major suggestion***: *Concerning the expression of CXCR4 (Fig 1F-I), the authors wrote (line 143) that a larger number of endothelial cells express the receptor: watching to Fig 1H and I, this observation is due to an increase of endothelial cell population between WTs and mutants, but the expression of CXCR4 per se is not increased.*

**Response:** We thank the Reviewer's insightful comments. To clarify whether the increase in CXCR4+ cells is related to a rise in the overall number of ECs in EC-specific *VHL* deletion mutants compared to the control littermates, we conducted whole-mount immunohistochemical staining using the EC nuclei marker ERG, and examined the total number of ERG+ ECs in both EC-specific *VHL* deletion mutants and the control littermates at E13.5. Our results indicate no significant difference in the number of ERG+ ECs between EC-specific *VHL* deletion mutants and the control littermates. This finding suggests that the increase in the number of CXCR4+ ECs in the mutants is not due to a rise in the total number of ECs, but rather due to ectopic expression of CXCR4 in these cells. We have now included these new data in **Figure S2** and have mentioned the corresponding findings in the RESULTS section in the revised manuscript (lines 149-154).

***Major suggestion***: *VHL inhibition stabilizes HIF that stimulates VEGF A. Nevertheless, the VEGF protein is unchanged between WT and mutants (FigS1 B-E): the authors discuss about a post-translational regulation that would restore VEGF A protein levels and autocrine signaling in the mutants (lines 366-367). However, authors also precise (line 365) that VEGF A is important for maintaining the vascular integrity. Thus, the recovery of VEGF A protein levels would have to*

© 2026. Published by The Company of Biologists under the terms of the Creative Commons Attribution License (https://creativecommons.org/licenses/by/4.0/).

*maintain a vessel integrity in mutants. The point has to be clarified for me. Furthermore, concerning patients with VHL syndrome that induces VEGF mRNA overexpression, what is known about the variation of VEGF at the protein level?*

**Response:** We thank the Reviewer's insightful comments.

- We apologize for the confusion caused by the descriptions in the original version of the manuscript. Currently, we do not have answers to explain the observations that, while *Vegfa* mRNA expression is upregulated, the level of VEGF-A protein does not change in EC-specific *VHL* deletion mutants. Therefore, in the Discussion section, we discuss a potential post-translational regulation that could restore VEGF-A levels in the mutants.
- Previous studies demonstrated that EC-specific *Vegfa* deletion leads to EC degeneration, indicating that autocrine VEGF-A is crucial for maintaining vascular integrity and cellular viability (Domigan et al. *J Cell Sci* 2015 PMID:25956888, Lee et al. *Cell* 2007 PMID:17719546). However, these studies did not identify the mechanisms responsible for maintaining VEGF-A protein levels in ECs.
- To clarify the text and avoid any confusion, we have revised the sentences in the DISCUSSION section in the revised manuscript (lines 480-489) as described below:

  "VEGF-A is expressed by capillary ECs in the developing skin vasculature. Interestingly, in EC-specific *VHL* deletion mutants, there is a significant increase in *Vegfa* mRNA expression, but no corresponding change in VEGF-A protein levels in capillary ECs. This observation may suggest a potential post-translational regulation of VEGF-A restores protein levels and autocrine signaling in these mutants. Previous studies in EC-specific deletion of *Vegfa* demonstrated that autocrine VEGF-A is important for maintaining vascular integrity and cellular viability (Domigan et al., 2015, Lee et al., 2007). However, the mechanisms that control VEGF-A expression in ECs remain unclear. Additionally, it is still uncertain whether the upregulation of VEGF-A by ECs affects their properties in VHL-related tumor vasculature, indicating a need for further investigation."

- VEGF-A protein levels in patients with VHL syndrome are notably elevated and remain consistently high. Therefore, anti-VEGF therapies have proven to be effective in treating clear cell renal cell carcinoma (ccRCC) with VHL mutations by reducing VEGF levels (reviewed in Chappell et al. *J Clin Invest* 2019 PMID:30614813). However, there are no published studies examining the variation of VEGF-A protein levels in ECs from patients with VHL syndrome. Conducting cell-type specific proteomics analysis could provide valuable insights into the protein levels of VEGF-A (and CXCR4) in ECs.

***Major suggestion**: Line 96: I do not understand what means "ectopic CXCR4 expression"?*

**Response:** We apologize for the confusion regarding the term "ectopic CXCR4 expression." As previously mentioned, our previous findings indicate that, before establishing nerve-vessel alignment at E15.5, a specific subset of ECs expresses CXCR4 in the capillary network at E13.5 (Li et al. *Dev Cell* 2013 PMID:23395391, Li et al. *Dev Biol* 2021 PMID:34015362). In contrast, both EC-specific *VHL* deletion mutants and EC-specific *Cxcr4* overexpression mutants show CXCR4 expression in most capillary ECs, as presented in **Figure 1F-I** in the revised manuscript. Therefore, we described the CXCR4 expression observed in these mutants as "ectopic CXCR4 expression."

***Major suggestion**: Concerning the expression of HIF-targeted angiogenic genes in ECs from EC-specific VHL deletion mutants (FigS1 A), I note that Notch expression is increased in deleted mice, while the difference is not significant (p=0.0635). Maybe that one or two more cases would permit to reach p<0.05 and it would be interesting to study Notch as it is known to play a role during vessel identity and patterning.*

**Response:** We thank the Reviewer for pointing out this.

- Based on the Reviewer's suggestion, we carefully re-evaluate *Notch1* expression using RT-qPCR analysis with previously prepared cDNA samples from FACS-isolated ECs from the skin of control littermates (n=4) and EC-specific *VHL* deletion mutant (n=4) embryos at E13.5.

© 2026. Published by The Company of Biologists under the terms of the Creative Commons Attribution License (https://creativecommons.org/licenses/by/4.0/). 

Our re-analysis shows that there is no significant alteration of *Notch1* expression in ECs between control littermates and EC-specific *VHL* deletion mutants.

- Unfortunately, due to unexpected challenges in our breeding colony of EC-specific *VHL* deletion mutants, we were unable to conduct an additional FACS-mediated isolation of ECs from the skin of control littermates and EC-specific *VHL* deletion mutants for further RT-qPCR analysis of *Notch1* expression.

- We also conducted RT-qPCR to assess *Notch1* expression in *VHL* knockdown HUVECs, and found no significant differences in *Notch1* expression between the control and *VHL* knockdown HUVECs. We have now included these new data in **Figures S14 and S15** and have mentioned the corresponding findings in the DISCUSSION section in the revised manuscript (lines 429-433).
- Previous studies (Arreola et al. *JCI Insight* 2018 PMID:29467323) have demonstrated that 1) the expression of certain components of Notch signaling pathway is upregulated in *VHL* knockout embryonic stem (ES) cell-derived ECs compared to wild-type ES cell-derived ECs, and 2) Notch inhibition can restore defective vessel identity and maturation in the developing retinal vasculature of *VHL* mutants during postnatal stages. Considering these findings, it is important to investigate the role of Notch signaling pathway in the embryonic skin vasculature model, and we fully intend to pursue this research in the future.

***Major suggestion***: *Watching to Fig 1L, only more or less 10% of endothelial cells express HIF in their nuclei: thus, how can this weak percentage explain the vascular abnormalities observed in the mutants?*

**Response:** We thank the Reviewer for the candid feedback and the opportunity to clarify the challenges we faced with immunohistochemical staining of embryonic limb skin with anti-HIFa antibody. Visualizing a significant fluorescent signal, particularly in the nuclei of ECs, in the HIF1a antibody staining was particularly difficult in these samples. Due to these technical issues, we were only able to capture ECs that displayed a strong and significant signal in the HIF1a antibody staining of EC-specific *VHL* deletion mutants. Therefore, we acknowledge that we may not have captured all ECs expressing nuclear HIF1a in these mutants. Nevertheless, the increase in the number of ECs expressing nuclear HIF1a in these mutants is still an important finding to report. To enhance the clarity and avoid any confusion, we have revised the sentences in the RESULTS section in the revised manuscript (lines 169-173) as described below:

"We should note that due to technical issues, we were only able to capture ECs that displayed a strong and significant signal in the HIF1a antibody staining of EC-specific *VHL* deletion mutants. Therefore, we acknowledge that we may not have captured all ECs expressing nuclear HIF1a in these mutants."

***Major suggestion***: *Concerning in vitro experiments, why do the authors use HUVECs rather than HUAECs or both cell lines?*

**Response:** To determine whether *VHL* knockdown induces CXCR4 expression, it is preferable to use ECs that have low CXCR4 expression. We decided to use HUVECs as our in vitro model for the following reasons: 1) HUVECs express VHL, but have low levels of CXCR4 expression, and 2) HUVECs have been used in vitro for studies on hypoxia and *VHL* knockdown (Wang et al. *Sci Rep* 2017 PMID:28710479, Lei et al. *Cells* 2020 PMID:32325871).

- In EC-specific *VHL* deletion mutants, ectopic CXCR4 expression is observed in capillary ECs, likely including venous capillaries. *VHL* knockdown in HUVECs may partially replicate these phenomena.

***Major suggestion***: *Furthermore, in Fig 2C, I do not understand why VHL siRNAs do not induce an increase of HIF 1A and HIF 2A expression?*

**Response:** As the Reviewer pointed out, we did not observe any alterations in the expression of *HIF1A* and *HIF2A* at the mRNA level in *VHL* knockdown HUVECs, presented in **Figure 2** in the revised manuscript. This finding can be explained by the role of the VHL ubiquitin ligase, which is

© 2026. Published by The Company of Biologists under the terms of the Creative Commons Attribution License (https://creativecommons.org/licenses/by/4.0/). 

responsible for the oxygen-dependent ubiquitylation and degradation of both HIF-1α and HIF-2α proteins. Therefore, VHL regulates the levels of these HIF-1α and HIF-2α at the protein level, rather than at the mRNA level (reviewed in Kaelin. *J Clin Invest* 2022 PMID:36106637, Kaelin and Ratcliffe. *Mol Cell* 2008 PMID:18498744).

***Major suggestion***: *Fig 3H, how do the authors explain the (significant?) decrease in branch points at E15.5 between VHL mutants and CXCR4 overexpression mice?*

**Response:** We thank the Review for this insightful question.

- As requested by the Reviewer, we compared the branching points at E13.5 and E15.5 between EC-specific *VHL* deletion mutants and EC-specific *Cxcr4* overexpression mutants. Our statistical analysis reveals that at E15.5, EC-specific *Cxcr4* overexpression mutants have significantly fewer branching points than EC-specific *VHL* deletion mutants. We have now included this new data in **Figure 3** and have mentioned the corresponding findings in the FIGURE LEGENDS section in the revised manuscript (lines 753-755).
- The difference in the branching points between these two mutants is likely attributed to two factors: 1) the number of ECs expressing CXCR4 and 2) the expression level of CXCR4 in ECs. EC-specific *Cxcr4* overexpression mutants appear to have a greater number of CXCR4+ ECs and higher levels of CXCR4 expression compared to EC-specific *VHL* deletion mutants.
- The difference in the number of ECs expressing CXCR4 and the expression level of CXCR4 in ECs between these two mutants is likely due to two distinct mechanisms that induce CXCR4 in ECs.
    - EC-specific *Cxcr4* overexpression mutants are double transgenic models (*Cad5-BAC-CreERT2; ROSA-loxP-STOP-loxP-Cxcr4-hGFP)* generated by crossing heterozygous *ROSA-loxP-STOP-loxP-Cxcr4-hGFP* mice with *Cad5-BAC-CreERT2* mice. In this case, CXCR4 is forcibly expressed in nearly all ECs and its expression level in ECs is regulated by the pan-*Rosa26* promoter (Li et al. *Dev Biol* 2021 PMID:34015362). In contrast, EC-specific *VHL* deletion mutants are also double mutant models (*Cad5-BAC-CreERT2; VHL-flox/flox)* generated by crossing homozygous *VHL-flox/flox* mice with *Cad5-BAC-CreERT2; VHL-flox/+* mice. In EC-specific *VHL* deletion mutants, HIF stabilization occurs due to VHL deletion, where CreERT2 needs to remove both floxed alleles, and the HIF-mediated activation of the endogenous *Cxcr4* promoter, are necessary for CXCR4 expression in ECs. This multistep process may enhance a mosaic expression pattern of CXCR4 in ECs.
    - In EC-specific *VHL* deletion mutants, HIF-targeted angiogenic genes, such as VEGF-A, may partially restore the reduction in branching points.

***Major suggestion***: *In the discussion, the authors wrote (line 357) that "in the absence of arterial branches, venous branching is impaired". However, I observed in Fig 6H and I that, while arterial branching is partially restored after AMD3100 treatment, it does not induce the venous alignment along arteries. Is there any explanation about this observation?*

**Response:** We thank the Review for this insightful question. Previous studies have demonstrated that remodeled venous branches form along arterial branches through endothelial apelin-APJ signaling pathway: arterial ECs express apelin, while its receptor APJ is expressed by venous ECs (Kidoya et al. *Dev Cell* 2015 PMID:25920569). Based on this, we hypothesized that EC-specific *VHL* deletion mutants after administration of AMD3100 displayed partially restored arterial branches that may not fully function to recruit venous branches. To address this further, we examined the expression of apelin and APJ in the developing skin vasculature. Unfortunately, none of the methods using commercially available antibodies to apelin and APJ provided adequate specificity for whole-mount immunohistochemical staining. At this time, we do not have answers about this matter. However, we fully intend to pursue this research in the future.

***Minor suggestion:*** *Line 62 add "the" between "is" and "vascular"*

**Response:** We thank the Reviewer's thorough review. We have corrected this in the revised manuscript.

© 2026. Published by The Company of Biologists under the terms of the Creative Commons Attribution License (https://creativecommons.org/licenses/by/4.0/).

***Minor suggestion:*** *Line 238 correct "(FigS3B)"*

**Response:** We apologize for this error and have corrected this in the revised manuscript (lines 270-273).

***Minor suggestion****: Line 476 M&M How many-time lasts the fixation?*

**Response:** We fixed the embryos in 10% neutral buffered formalin for at least 24 hours at room temperature. We have now included the information in the MATERIALS AND METHODS section in the revised manuscript (lines 604-605).

***Minor suggestion****: Figure 1J and K The lumen of vessels has to be indicated to clarify what the reader watches.*

**Response:** As requested by the Reviewer, we have now indicated the lumen of vessels with white dotted lines in **Figure 1** and have mentioned it in the FIGURE LEGENDS section in the revised manuscript (lines 705-706).

**REVIEWER 2**

***Comments****: …..This study clarifies a regulatory and functional connection between VHL-HIF1 and CXCR4 signaling, providing evidence that VHL regulation of HIF target gene expression is endothelial cell-autonomously required for normal vascular development. The characterization of arterial, venous, and lymphatic vessel phenotypes is well interrogated, and the data is clearly presented and convincing. The immunofluorescent images are noteworthy and of exceptional quality.*

*I have several comments and questions that if addressed would further enhance the impact of this study.*

**Response**: We greatly appreciate the Reviewer's encouraging feedback. We agree that the additional data and information addressing the Reviewers' comments and questions significantly greatly enhance the quality of our manuscript.

***Major suggestion #1:*** *The authors employed a floxed Vhl allele inactivated by a Cad5-driven CreERT2. Was the extent of VHL depletion in endothelial cells examined? It would be informative to know if the Vhl gene was ablated completely or partially.*

**Response:** We thank the Reviewer's question. To evaluate the efficiency of *VHL* gene deletion in ECs, we first isolated ECs from the limb skins of both control littermates and EC-specific *VHL* deletion mutants using FACS. Since the *VHL-flox/flox* mice contain *loxP* sites flanking exon 1 of the *VHL* gene (Haase et al. *Proc Natl Acd Sci USA* 2001 PMID:11171994), we designed primers within exon1 of the *VHL* gene for RT-qPCR analysis. Our results indicate that *VHL* expression was partially reduced in EC-specific *VHL* deletion mutants compared to control littermates ($p$=0.0286). This reduction is likely due to a mixture of ECs in which CreERT2 has removed both floxed alleles, only one allele, or none. We have now included this new data in **Figure S1** and have mentioned the corresponding findings in the RESULTS section in the revised manuscript (lines 129-131).

***Major suggestion #2:*** *In figure S1 the authors show that of the HIF1$\alpha$ target genes assessed, Vegfa was the most transcriptionally upregulated in Vhl cKO embryos (~3 fold). Surprisingly, however, endothelial levels of VEGFA protein are apparently unaltered. I find this to be rather unexpected and difficult to interpret based on the immunohistochemical methods used. The comparison to CXCR4, which shows a clear increase on endothelial cells despite a modest mRNA upregulation*

© 2026. Published by The Company of Biologists under the terms of the Creative Commons Attribution License (https://creativecommons.org/licenses/by/4.0/). 

*(~1.5 fold), cannot be made as it is a plasma membrane protein whereas VEGFA is secreted. A more appropriate method would be to perform an ELISA on tissue homogenates or on conditioned media from isolated WT versus VHL-null endothelial cells.*

**Response:** We thank the Reviewer for these insightful comments.

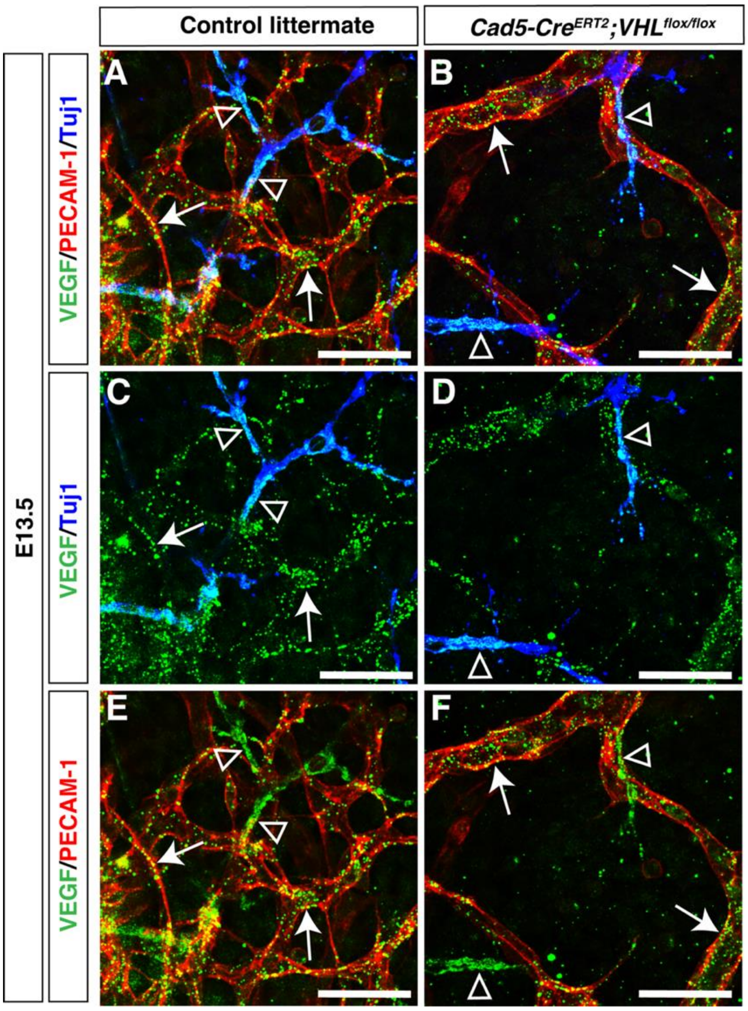


**Figure for the Reviewer 2. VEGF-A expression in peripheral nerves and capillary endothelial cells.** Whole-mount immunohistochemical analysis of limb skin with antibodies to VEGF (A-F, green), PECAM-1 (A-B, E-F, red), and Tuj1 (A-D, blue) in control littermates (A, C, E) and *Cad5-BAC-Cre*$^{ERT2}$;*VHL*$^{flox/flox}$ (B, D, F) embryos at E13.5 are shown. Arrows indicate representative VEGF-expressing ECs in the capillary network. Open arrowheads indicate VEGF-expressing peripheral nerves. Scale bars represent 50 μm.

- As noted, detecting secreted factors, such as VEGF-A, in tissues is challenging. We have experienced conducting primary cultures of FACS-isolated ECs from E13.5~E15.5 embryos; however, it is difficult to maintain a long-term primary culture that yields enough supernatant for an ELISA assay. Additionally, primary cultures of these FACS-isolated ECs require VEGF-A in the culture medium to support their growth and maintenance. Due to these technical challenges, we are unable to evaluate secreted VEGF-A at the protein level as the Reviewer requested.
- It is important to note that we thoroughly evaluated the anti-VEGF-A antibody (R&D) and ensured it had adequate specificity for whole-mount immunohistochemical staining. Indeed, we clearly detected VEGF expression in sensory nerves, presented in **Figure S3** in the revised manuscript and **Figure for the Reviewer 2** in this letter, consistent with our previously report (Mukouyama et al. *Cell* 2002 PMID:12086669, Li et al. *Dev Cell* 2013 PMID:23395391).

© 2026. Published by The Company of Biologists under the terms of the Creative Commons Attribution License (https://creativecommons.org/licenses/by/4.0/). 

- However, we cannot fully explain the observation that, while *Vegfa* mRNA expression is upregulated, the level of VEGF-A protein does not change in EC-specific *VHL* deletion mutants. Therefore, in the Discussion section, we discuss a potential post-translational regulation that could restore VEGF-A levels in the mutants (lines 480-489).

***Major suggestion #3:*** *The authors chose to narrow their investigation on a relatively small subset of HIF1$\alpha$ target genes, namely relevant to angiogenesis (e.g. Ang1, Ang2). Were other well-recognized HIF1$\alpha$ targets interrogated? For example, regulators of glycolysis (e.g. Pgk1, Pdk1, Hk2) would inform on metabolic alteration in ECs. Also, changes in adrenomedulin (Adm), a specific HIF1$\alpha$ target and a regulator of lymphangiogenesis (Fritz-Six, JCI, 2008), may be relevant to the lymphatic defects and edema exhibited by VHL mutants. Perhaps molecular profiling (RNAseq) could provide a broader perspective on pathway perturbations resulting from endothelial VHL loss of function.*

**Response:** We thank the Reviewer's suggestion.

- In response to the Reviewer's suggestion, we expanded our RT-qPCR analysis to include HIF1$\alpha$ target genes that are involved in endothelial metabolism. Our results indicate that the expression of metabolic regulators in ECs did not show significant changes in EC-specific *VHL* deletion mutants compared to control littermates. We have now included these new data in **Figure S1** and have mentioned the corresponding findings in the RESULTS section in the revised manuscript (lines 131-139).
- We also conducted RT-qPCR analysis to examine *Adm* expression in FACS-isolated ECs. Our results indicate that *Adm* expression was significantly increased at the mRNA level in EC-specific *VHL* deletion mutants compared to control littermates. We have now included this new data in **Figure S1** and have mentioned the corresponding findings in the RESULTS section in the revised manuscript (lines 139-146).
  - Given that 1) overexpression of *Adm* promotes lymphangiogenesis (Trincot et al. *Circ Res* 2019 PMID:30582443) and 2) lymphatic EC-specific *VHL* deletion mutants or *Cxcr4* overexpression mutants do not exhibit any lymphatic abnormalities, it is possible that the excess Adm expression derived from ECs may contribute to the lymphatic abnormalities observed in EC-specific *VHL* deletion mutants.
- Due to unexpected budgetary issues within the NIH intramural program arising from recent Presidential Executive Orders, we opted to conduct RT-qPCR analysis to examine the expression of HIF-targeted angiogenic genes and endothelial metabolic genes, instead of conducting bulk RNA sequencing. We appreciate the Reviewer's understanding.

***Major suggestion #4:*** *In figure 6, the authors performed studies to nicely demonstrate the rescue of VHL LOF phenotypes by CXCR4 antagonism. Have they considered performing similar experiments I n the CXCR4 OE model?*

**Response:** We thank the Reviewer's suggestion regarding the rescue experiments on EC-specific *Cxcr4* overexpression mutants using AMD3100. Although we acknowledge the potential value of such experiments, however, we do not plan to include this approach. Our primary focus remains on conducting the rescue experiments on EC-specific *VHL* deletion mutants using AMD3100. This focus is most aligned with our goal of addressing whether the VHL-Cxcr4 axis contributes to the vascular abnormalities in these mutants.

***Major suggestion #5:*** *The authors focused on dermal vessel phenotypes, but I wonder if they observed defects in specific organs as well (heart, lung, kidney)?*

**Response:** Previous studies, including our own, have demonstrated that in embryonic heart, vascular remodeling of capillary plexus leads to the formation of a branched, hierarchical vascular structure and CXCL12-CXCR4 signaling plays essential roles in coronary vascular development (reviewed in Bautch and Mukouyama. *Cold Spring Harb Perspect Med* 2022 (PMID:35379659). Based on these findings, we decided to investigate coronary vessel phenotypes in EC-specific *VHL* deletion mutants. Consistent with our previous findings that coronary vascular development is impaired in

© 2026. Published by The Company of Biologists under the terms of the Creative Commons Attribution License (https://creativecommons.org/licenses/by/4.0/). 

EC-specific *Cxcr4* overexpression mutants (Li et al. *Dev Biol* 2021 PMID:34015362), our results indicate disorganized coronary vascular development in EC-specific *VHL* deletion mutant hearts, with large-diameter venous and arterial branches being undetectable in these mutants compared to control littermates. We have now included this new data in **Figure S8** and have mentioned the corresponding findings in the RESULTS section in the revised manuscript (lines 277-290).

***<u>Major suggestion #6</u>:*** *Suggestion for future study: the alleles characterized here perfectly lend themselves for performing studies in the retinal angiogenesis model. This well-established model offers several advantages including well-defined and manipulable developmental stages, stereotypical venous and arterial specification, and clearly delineated angiogenic domains, all amenable for quantification. I wonder if the authors have performed confirmatory experiments in this model or are planning to in future studies.*

**Response:** We thank the Reviewer for this insightful suggestion. Since the retinal vascular malformation associated with the *VHL* mutations has been characterized (reviewed in Chappell et al. *J Clin Invest* 2019 PMID:30614813), we plan to investigate vessel phenotypes in the retinal vasculature of EC-specific *VHL* deletion mutants and EC-specific *Cxcr4* overexpression mutants in future studies.

**<u>REVIEWER 3</u>**

***<u>Comments</u>***: *…..Overall, the experiments are well done, data clear to appreciate, and manuscript is well-written. Although the phenotypes of VHL-ECKO and CXCR4-OE models are not new the mechanistic connection between the two seems to be novel. Nevertheless, I do have a few additional experiments to make the manuscript stronger.*

**Response**: We greatly appreciate the Reviewer's encouraging comments and recognition of our mechanistic findings regarding the ectopic activation of HIF-CXCR4 signaling axis in EC-specific *VHL* deletion. We agree that the additional data and information—particularly concerning lymphatic vessel phenotypes—address the Reviewer's comments, clarifying our results and conclusions and significantly enhancing our manuscript.

***<u>Major suggestion #1:</u>*** *Blood-lymph separation is regulated by the glycoprotein podoplanin in LECs and C-type lectin CLEC2 in platelets. Check whether the expression of podoplanin is normal in LECs. Determine whether platelets are normally present in the VHL-ECKO and CXCR4 OE embryos.*

**Response:** We thank the Reviewer for this suggestion.

- To assess podoplanin (PDPN) expression in lymphatic ECs (LECs) within the lymph sac, we conducted immunohistochemical staining with an anti-PDPN antibody on trunk sections of EC-specific *VHL* deletion and EC-specific *Cxcr4* overexpression mutants at E13.5. Our results indicate a decrease in PDPN expression in LYVE-1+ LECs in the lymph sac of these mutants compared to control littermates.
- To examine platelet distribution at the blood-lymph separation, we conducted immunohistochemical staining with the platelet marker CD41 on E13.5 trunk sections of EC-specific *VHL* deletion and EC-specific *Cxcr4* overexpression mutants. Our results indicate that while CD41+ platelets accumulate at the boundary between the jugular vein and the lymph sac in control littermates, fewer CD41+ pericytes are observed in the fused lymph sac-jugular vein vessels of these mutants.
- These findings suggest that the impaired blood-lymph separation in these mutants can be attributed to disrupted CLEC2-PDPN signaling between platelets and LECs. However, the mechanisms by which the upregulation of endothelial CXCR4 expression leads to dysregulation of CLEC2-PDPN signaling at the blood-lymph separation requires further investigation. We have now included these new data in **Figure S10** and have mentioned the corresponding findings in the RESULTS section (lines 322-333) in the revised manuscript.

© 2026. Published by The Company of Biologists under the terms of the Creative Commons Attribution License (https://creativecommons.org/licenses/by/4.0/).

***Major suggestion #2-1***: *The authors are suggesting a connection between lymph sacs and jugular vein in VHL KO and CXCR4 OE embryos. These images are very convincing. Better images can be provided?*

**Response:** As requested by the Reviewer, we have now included new images of control littermates, which clearly show the separation between the jugular vein and the lymph sac in **Figure 5J and M** of the revised manuscript. However, the revised **Figure 5K and N**, which depict EC-specific *VHL* deletion, as well as **Figure 5L and O** for EC-specific *Cxcr4* overexpression mutants, remain the same as those in the original version of the manuscript. We believe that these figures present the fused lymph sac-jugular vein vessels in these mutants.

***Major suggestion #2-2***: *Additionally, blood and lymphatic vasculatures interact through lymphovenous valves. Whether these valves are normal in VHL-ECKO and CXCR4 OE embryos must be determined. Whether these defects are ameliorated by AMD3100 must be tested.*

**Response:** We thank the Reviewer for this suggestion.

- To examine lymphovenous valves (LVVs), we conducted immunohistochemical staining with the venous marker EMCN and the LEC marker LYVE-1 together with the pan-EC marker PECAM-1 on E15.5 trunk sections of EC-specific *VHL* deletion and EC-specific *Cxcr4* overexpression mutants. Our results indicate no significant difference in the number of LVVs between control littermates and these mutants.
- We further analyzed LVVs in EC-specific *VHL* deletion mutants after administration of AMD3100. Similarly, we did not detect any significant changes in LVVs in these mutants with AMD3100.
- We have now included these new data in **Figure S11** and have mentioned the corresponding findings in the RESULTS section (lines 335-344) and the FIGURE LEGEND for **Figure S11** in the revised manuscript.

***Major suggestion #3***: *The authors previously showed that the OE of CXCR4 in LECs does not result in blood-filled lymphatics. They must test whether deletion of VHL from LECs results in any phenotype.*

**Response:** As requested by the Reviewer, we generated LEC-specific *VHL* deletion mutants using LEC-specific *Prox-1-Cre* driver mice. Our results indicate no apparent abnormalities in gross morphology and embryonic size in LEC-specific *VHL* deletion mutants compared to control littermates. Whole-mount immunohistochemical staining with the erythrocyte maker Ter119 and the LEC marker PDPN revealed no blood-filled lymphatic vascular phenotype. These findings suggest that the defects in blood-filled lymphatic vasculature observed in EC-specific *VHL* deletion mutants are not the primary defects. We have now included these new data in **Figure S9** and have mentioned the corresponding findings in the RESULTS section in the revised manuscript (lines 303-310).

- As the Reviewer noted, we generated LEC-specific *Cxcr4* overexpression mutants (Li et al. *Dev Biol* 2021 PMID:34015362). Our results also indicate no blood-filled lymphatic vascular phenotype in these mutants. Similarly, these findings suggest that the defects in blood-filled lymphatic vasculature observed in EC-specific *Cxcr4* overexpression mutants are not the primary defects.

***Minor suggestion #1***: *Bulk RNA-seq experiments are reasonably inexpensive to do. It will be interesting to generate this data for siVHL, siHIF1A+siHIF1B and siVHL+siHIF1A+siHIF1B samples.*

**Response:** We thank the Reviewer for this insightful suggestion.

- HIF1A and HIF2A are the two main alpha subunits of HIF, and hydroxylated forms HIF1A and HIF2A are recognized by the VHL ubiquitin ligase, which is responsible for the oxygen-dependent ubiquitylation and degradation of both HIF1A and HIF2A proteins. To assess whether *VHL* loss leads to the upregulation of CXCR4 expression through HIF1A and HIF2A, we compared CXCR4 expression between control, *VHL* knockdown, and *VHL/HIF1A/HIF2A*

© 2026. Published by The Company of Biologists under the terms of the Creative Commons Attribution License (https://creativecommons.org/licenses/by/4.0/). 

triple-knockdown HUVECs. We further conducted RT-qPCR analysis to examine the expression of HIF-targeted angiogenic genes and endothelial metabolic genes in these cells. We have now included these new RT-qPCR data in the revised **Figure S14** and have mentioned the corresponding findings in the DISCUSSION section in the revised manuscript (lines 439-444).

- HIF1B is the beta subunit that is constitutively expressed and not regulated by the VHL ubiquitin ligase. HIF1B forms heterodimers with either HIF1A or HIF2A. As requested by the Reviewer, we examined the expression of CXCR4, as well as HIF-targeted angiogenic genes and endothelial metabolic genes between control, *VHL* knockdown, and *VHL*/*HIF1A*/*HIF1B* triple-knockdown HUVECs. We have now included these new RT-qPCR data in the revised **Figure S15** and have mentioned the corresponding findings in the DISCUSSION section in the revised manuscript (lines 444-451).
- Due to unexpected budgetary issues within the NIH intramural program arising from recent Presidential Executive Orders, we opted to conduct qRT-PCR analysis to examine the expression of HIF-targeted angiogenic genes and endothelial metabolic genes, instead of conducting bulk RNA sequencing. We appreciate the Reviewer’s understanding.

---

Second decision letter

MS ID#: dev.204519R1

MS Title: Endothelial Von Hippel-Lindau Deletion Causes Abnormal Vascular Network Formation Through Ectopic Activation of Hif-Cxcr4 Axis

Authors: Wenling Li; Koh Nakayama; Ryo Sato; Rina Shimada; Yoshiaki Kubota; Yoh-suke Mukouyama
Article Type: Research Article

Dear Dr Mukouyama,

I have now received all the referees reports on the above manuscript, and have reached a decision. The referees' comments are appended below, or you can access them online: please go to View Reviewer Comments.

The overall evaluation is positive and we would like to publish a revised manuscript in Development, provided that the referees' comments can be satisfactorily addressed. Please attend to all of the reviewers' comments in your revised manuscript and detail them in your point-by-point response. If you do not agree with any of their criticisms or suggestions explain clearly why this is so. If it would be helpful, you are welcome to contact us to discuss your revision in greater detail. Please send us a point-by-point response indicating your plans for addressing the referees’ comments, and we will look over this and provide further guidance.

Reviewer 1

SUMMARY OF THE ADVANCE MADE IN THIS PAPER AND ITS POTENTIAL SIGNIFICANCE TO THE FIELD

I thank the authors for their detailed answers that clarify the different points I mentioned previously and recommend the publication of this impressive study.

Luc Pardanaud Ph.D
PARCC Inserm u970
Université Paris Cité

Reviewer 2

© 2026. Published by The Company of Biologists under the terms of the Creative Commons Attribution License (https://creativecommons.org/licenses/by/4.0/). 

SUMMARY OF THE ADVANCE MADE IN THIS PAPER AND ITS POTENTIAL SIGNIFICANCE TO THE FIELD

The role of VHL in regulating HIF1a during hypoxia has been well studied. Your study highlights the endothelial cell-autonomous role of VHL and reveals its regulation of CXCR4, an important for angiogenic vessel development.

SUGGESTIONS TO AUTHORS

I appreciate your efforts in addressing my suggestions. I particularly realize that assessing VEGFA protein in tissues remains a challenge, and that the field still does not know the relative levels of its isoforms (121/165/189) in normal tissues or tumors.
I hereby support the acceptance of your nice study for publication in Development.

Reviewer 3

SUMMARY OF THE ADVANCE MADE IN THIS PAPER AND ITS POTENTIAL SIGNIFICANCE TO THE FIELD

In this manuscript Li et al report that the deletion of VHL from endothelial cells (ECs) results in the overexpression of HIF1A and its target molecule CXCR4. A loss of arterial identity, reduced vascular branching and blood-filled lymphatics were observed in mice lacking VHL in ECs (VHL-ECKO). They previously showed that the overexpression (OE) of CXCR4 in blood ECs, but not lymphatic endothelial cells (LECs) result in a similar phenotype. Therefore, they hypothesized that the CXCR4 OE in ECs is the primary cause of the vascular phenotype in VHL-ECKO mice. They tested this hypothesis by treating control and VHL-ECKO embryos with the CXCR4 antagonist AMD3100 and demonstrate a partial rescue of the phenotypes.
Overall, the experiments are well done, data clear to appreciate and manuscript is well-written. Although the phenotypes of VHL-ECKO and CXCR4-OE models are not new the mechanistic connection between the two seems to be novel.

I have a couple of minor comments/suggestions for the authors.

Lymphovenous valves are the structures that regulate the interaction between lymph sacs and jugular vein. Responding to my comment the authors have shown that these valves are normal in mice lacking VHL or over expressing CXCR4 (Fig S11). Therefore, presumably the abnormal connection between lymph sacs and jugular vein must be located more anterior to the valves. The authors can probably elaborate about the location of these abnormal connections.

The title can be modified to better reflect the blood and the lymphatic phenotypes of the models. Something along the lines of "ENDOTHELIAL VON HIPPEL-LINDAU DELETION CAUSES ABNORMAL blood and lymphatic VASCULAR NETWORK FORMATION THROUGH ECTOPIC ACTIVATION OF HIF-CXCR4 AXIS".

---

**Second revision**

Author response to reviewers' comments

We are pleased to hear that Reviewers 1 and 2 are satisfied with our revised manuscript and support it for publication in *Development*. We appreciate Reviewer 3’s insightful suggestions so we here address them as follows.

***Reviewer 3:***

***Comments**:….Overall, the experiments are well done, data clear to appreciate and manuscript is well-written. Although the phenotypes of VHL-ECKO and CXCR4-OE models are not new the mechanistic connection between the two seems to be novel.*

© 2026. Published by The Company of Biologists under the terms of the Creative Commons Attribution License (https://creativecommons.org/licenses/by/4.0/). 

**Response:** We greatly appreciate the reviewer for the positive feedback on our study and for acknowledging that our work establishes a novel mechanistic connection between the *VHL-ECKO* and *CXCR4-OE* models. We are also grateful for the reviewer's insightful comments and suggestions throughout the review process, which have significantly improved our manuscript.

***Comments***: *Lymphovenous valves are the structures that regulate the interaction between lymph sacs and jugular vein. Responding to my comment the authors have shown that these valves are normal in mice lacking VHL or over expressing CXCR4 (Fig S11). Therefore, presumably the abnormal connection between lymph sacs and jugular vein must be located more anterior to the valves. The authors can probably elaborate about the location of these abnormal connections.*

**Response:** We appreciate the reviewer's insightful suggestion. To address this issue, we reanalyzed the E15.5 trunk sections of control littermates, *VHL-ECKO* and *CXCR4-OE* mutant embryos. Immunostaining with antibodies to the venous marker EMCN and the LEC marker LYVE-1 together with the pan-EC marker PECAM-1 revealed that the impaired blood-lymph separation was more pronounced anterior to the LVV in these mutants. However, we did not observe a significant difference in the number of LVVs between control littermates and these mutants, as presented in Figure for the Reviewer 3 in this letter. These findings support the reviewer's suggestion. We have mentioned this observation in the Discussion section in the revised manuscript (lines 494-503).

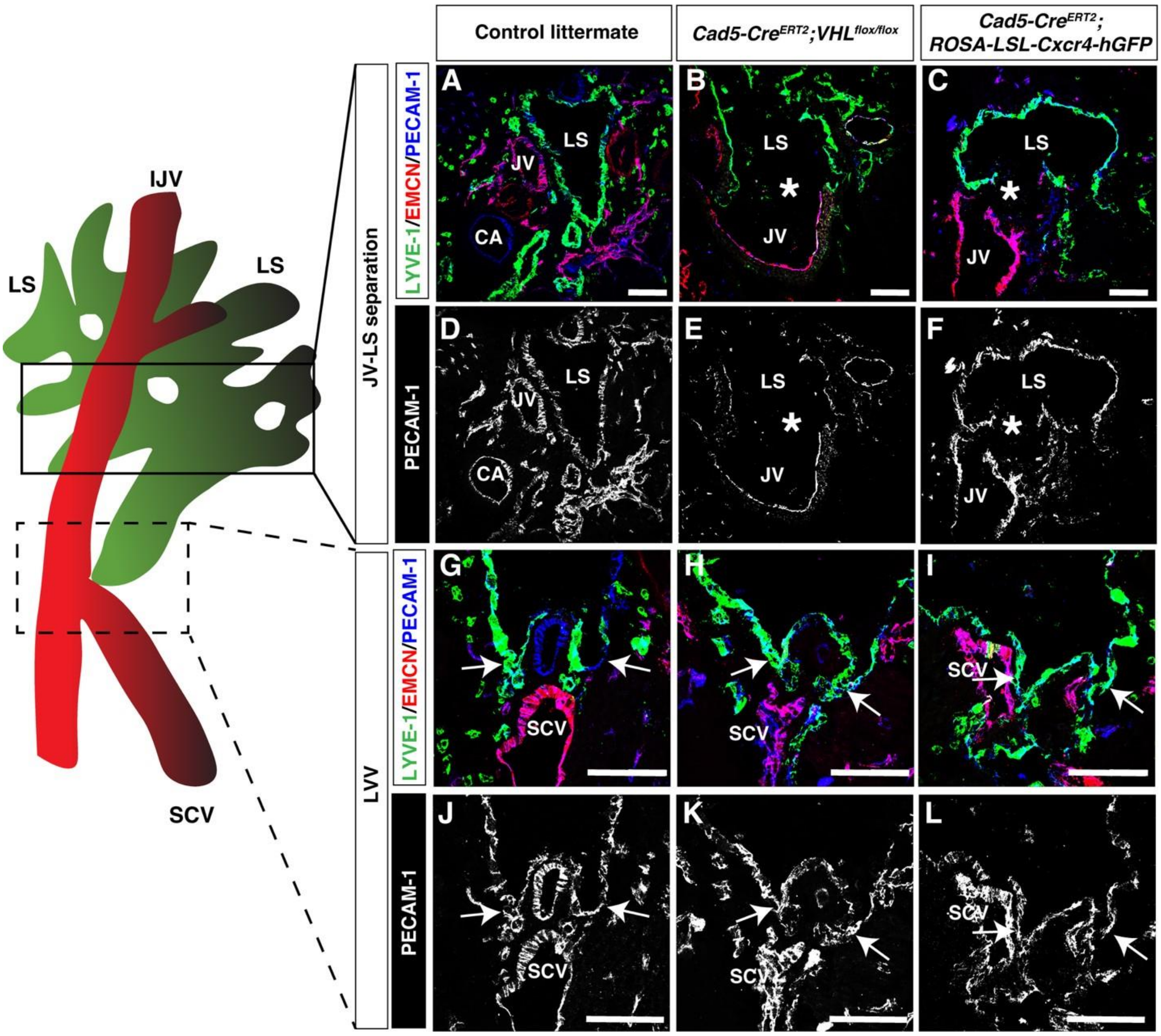


© 2026. Published by The Company of Biologists under the terms of the Creative Commons Attribution License (https://creativecommons.org/licenses/by/4.0/). 

**Figure for the Reviewer 3. Incomplete separation of lymph sac from jugular veins, but no apparent defects in lymphovenous valves, in EC-specific *VHL* deletion and EC-specific *Cxcr4* overexpression mutants.** Section immunohistochemical analysis of E15.5 trunk of control littermates (A, D, G, J), *Cad5-BAC-Cre$^{ERT2}$;VHL$^{flox/flox}$* (B, E, H, K), and *Cad5-BAC-Cre$^{ERT2}$;ROSA- LSL-Cxcr4-hGFP* (C, F, I, L) embryos with antibodies to LYVE-1 (green), endomucin (EMCN, red) and PECAM-1 (A-C, G-I, blue; D-F, J-L, white) are shown. Asterisk indicates the incomplete separation of lymph sac from jugular veins, while arrows indicate the lymphovenous valves. Note that the panels (G-L) are the same as those presented in Fig. S11. IJV: Internal jugular vein. LS: Lymph sac. SCV: Subclavian. Scale bars represent 100 μm. Schematic model was modified from Young and Oliver, *JCI* (2014)

***<u>Comments</u>**: The title can be modified to better reflect the blood and the lymphatic phenotypes of the models. Something along the lines of "ENDOTHELIAL VON HIPPEL- LINDAU DELETION CAUSES ABNORMAL blood and lymphatic VASCULAR NETWORK FORMATION THROUGH ECTOPIC ACTIVATION OF HIF-CXCR4 AXIS".*

**Response:** As suggested by the reviewer, we have modified the title to "*ENDOTHELIAL VON HIPPEL-LINDAU DELETION CAUSES ABNORMAL BLOOD AND LYMPHATIC VASCULATURE THROUGH ECTOPIC ACTIVATION OF HIF-CXCR4 AXIS*". This title has 116 characters, which is under 120 characters required by the journal's guidelines.

---

<u>Third decision letter</u>

MS ID#: dev.204519R2

MS Title: Endothelial Von Hippel-Lindau Deletion Causes Abnormal Blood and Lymphatic Vasculature Through Ectopic Activation of Hif-Cxcr4 Axis

Authors: Wenling Li; Koh Nakayama; Ryo Sato; Rina Shimada; Yoshiaki Kubota; Yoh-suke Mukouyama
Article Type: Research Article

Dear Dr Mukouyama,

I am happy to tell you that your manuscript has been accepted for publication in Development, pending our standard publication integrity checks.

© 2026. Published by The Company of Biologists under the terms of the Creative Commons Attribution License (https://creativecommons.org/licenses/by/4.0/).
